# Spectrum of Neurological Symptoms in Glycosylphosphatidylinositol Biosynthesis Defects: Systematic Review

**DOI:** 10.3389/fneur.2021.758899

**Published:** 2022-01-04

**Authors:** Justyna Paprocka, Michał Hutny, Jagoda Hofman, Agnieszka Tokarska, Magdalena Kłaniewska, Krzysztof Szczałuba, Agnieszka Stembalska, Aleksandra Jezela-Stanek, Robert Śmigiel

**Affiliations:** ^1^Department of Pediatric Neurology, Faculty of Medical Sciences in Katowice, Medical University of Silesia, Katowice, Poland; ^2^Students' Scientific Society, Department of Pediatric Neurology, Faculty of Medical Sciences in Katowice, Medical University of Silesia, Katowice, Poland; ^3^Department of Pediatrics and Developmental Age Neurology, Upper Silesian Child Health Centre, Katowice, Poland; ^4^Department of Pediatrics, Medical University of Wroclaw, Wroclaw, Poland; ^5^Department of Medical Genetics, Medical University of Warsaw, Warsaw, Poland; ^6^Department of Genetics, Medical University of Wroclaw, Wroclaw, Poland; ^7^Department of Genetics and Clinical Immunology, National Institute of Tuberculosis and Lung Diseases, Warsaw, Poland

**Keywords:** glycosylphosphatidylinositol biosynthesis defects, seizures, development delay, intellectual disability, hypotonia

## Abstract

**Background:** Mutations of genes involved in the synthesis of glycosylphosphatidylinositol and glycosylphosphatidylinositol-anchored proteins lead to rare syndromes called glycosylphosphatidylinositol-anchored proteins biosynthesis defects. Alterations of their structure and function in these disorders impair often fundamental processes in cells, resulting in severe clinical image. This study aimed to provide a systematic review of GPIBD cases reports published in English-language literature.

**Methods:** The browsing of open-access databases (PubMed, PubMed Central. and Medline) was conducted, followed by statistical analysis of gathered information concerning neurological symptomatology. The inclusion criteria were: studies on humans, age at onset (<18 y.o.), and report of GPIBD cases with adequate data on the genetic background and symptomatology. Exclusion criteria were: publication type (manuscripts, personal communication, review articles); reports of cases of GPI biosynthesis genes mutations in terms of other disorders; reports of GPIBD cases concentrating on non-neurological symptoms; or articles concentrating solely on the genetic issues of GPI biosynthesis. Risk of bias was assessed using Joanna Brigs Institute Critical Appraisal Checklists. Data synthesis was conducted using STATISTICA 13.3.721.1 (StatSoft Polska Sp. z.o.o.). Used tests were chi-square, Fisher's exact test (for differences in phenotype), and Mann-Whitney U test (for differences in onset of developmental delay).

**Results:** Browsing returned a total of 973 articles which, after ruling out the repetitions and assessing the inclusion and exclusion criteria, led to final inclusion of 77 articles (337 GPIBD cases) in the analysis. The main outcomes were prevalence of neurological symptoms, onset and semiology of seizures and their response to treatment, and onset of developmental delay. Based on this data a synthesis of phenotypical differences between the groups of GPIBD cases and the general GPIBD cases population was made.

**Discussion:** A synthetical analysis of neurological components in clinical image of GPIBD patients was presented. It highlights the main features of these disorders, which might be useful in clinical practice for consideration in differential diagnosis with children presenting with early-onset seizures and developmental delay. The limitation of this review is the scarcity of the specific data in some reports, concerning the semiology and onset of two main features of GPIBD.

## Introduction

Glycosylphosphatidylinositol (GPI) is a phosphoglycerate, playing the central axis role in GPI-anchored protein (GPI-AP) multi-stage synthesis pathway, engaging enzymes coded by a variety of genes. As GPI-APs are involved in numerous processes in the human organism, defects of genes, which lead to impairment of their creation, cause a wide range of pathologies known as GPI biosynthesis defects (GPIBD). Even though the incidence of such disorders is relatively low, their symptoms are usually severe, including neurological features like encephalopathy, delayed psychomotor development, hypotonia, seizures, and many more. Despite the numerous articles presenting patients suffering from GPIBD or analyzing genome-phenotype relations in them, only a few studies summarize these diseases, and no articles present the synthesis of their neurological aspects. The objective of this systematic review is to establish a concise summary of neurological symptomatology in disorders caused by mutations in genes involved in GPI biosynthesis.

## Materials and Methods

### Eligibility Criteria

In Our Systematic Review We Included Only Original Research Articles Reporting the Cases of Patients With GPIBD With Adequate Data on the Genetic Background and Symptomatology, Including Case Reports, Case Series, and Observational (Prevalence) Studies, Published in English. Author Manuscripts, Personal Communication, and Review Articles Were Excluded, as Were the Studies Solely on Genetic Issues of GPI Biosynthesis, Reports of Cases of GPI Biosynthesis Genes Mutations in Terms of Disorders Other Than GPIBD, and Reports of GPIBD Cases Concentrating on non-Neurological Symptoms.

### PECO Framework

According to the Population, Exposure, Comparator, Outcome (PECO) framework the following question was formulated: “What are the differences in prevalence of neurological symptoms (O) between the general population of children (P) with GPI biosynthesis defect (E) in comparison to the groups of children with each individual genetic defect of GPI (C)?”. The participants of the included studies were children (age at onset <18 y.o.) diagnosed with GPIBD. The comparison was conducted between the general population of GPIBD patients, and each specific group of GPIBD patients with mutation in specific gene associated with GPI biosynthesis. The domains of comparison include quantitative and qualitative clinical features.

### Information Sources

Over the span of November 2020–September 2021 the PubMed, PubMed Central, and Medline databases were browsed for records presenting cases of GPIBDs, describing the phenotypes and genotypes of these patients.

### Search Strategy

Entry terms were created using the following scheme:

- “ARV1” OR “DPM1” OR “DPM2” OR “DPM3” OR “MPDU1” OR “GPAA1” OR “PGAP1” OR “PGAP2” OR “PGAP3” OR “PGAP6” OR “PIGA” OR “PIGB” OR “PIGC” OR “PIGF” OR “PIGG” OR “PIGH” OR “PIGK” OR “PIGL” OR “PIGM” OR “PIGN” OR “PIGO” OR “PIGP” OR “PIGQ” OR “PHOSPHATIDYLINOSITOL GLYCAN ANCHOR BIOSYNTHESIS CLASS S PROTEIN” OR “PIGT” OR “PIGU” OR “PIGV” OR “PIGW” OR “PIGY” OR “PIGX” OR “PIGZ”

AND

- “GPI” OR “biosynthesis” OR “GPIBD” OR “CDG”

This strategy was applied for browsing of all databases. The records were filtered in terms of language–no time filter was applied, as the review aimed at including all cases available in open-source literature.

### Selection Process

After exclusion of repetitions, retrieved records were then screened on the basis of titles and abstracts, by two independent reviewers (MH and JH).

**Figure 1:**
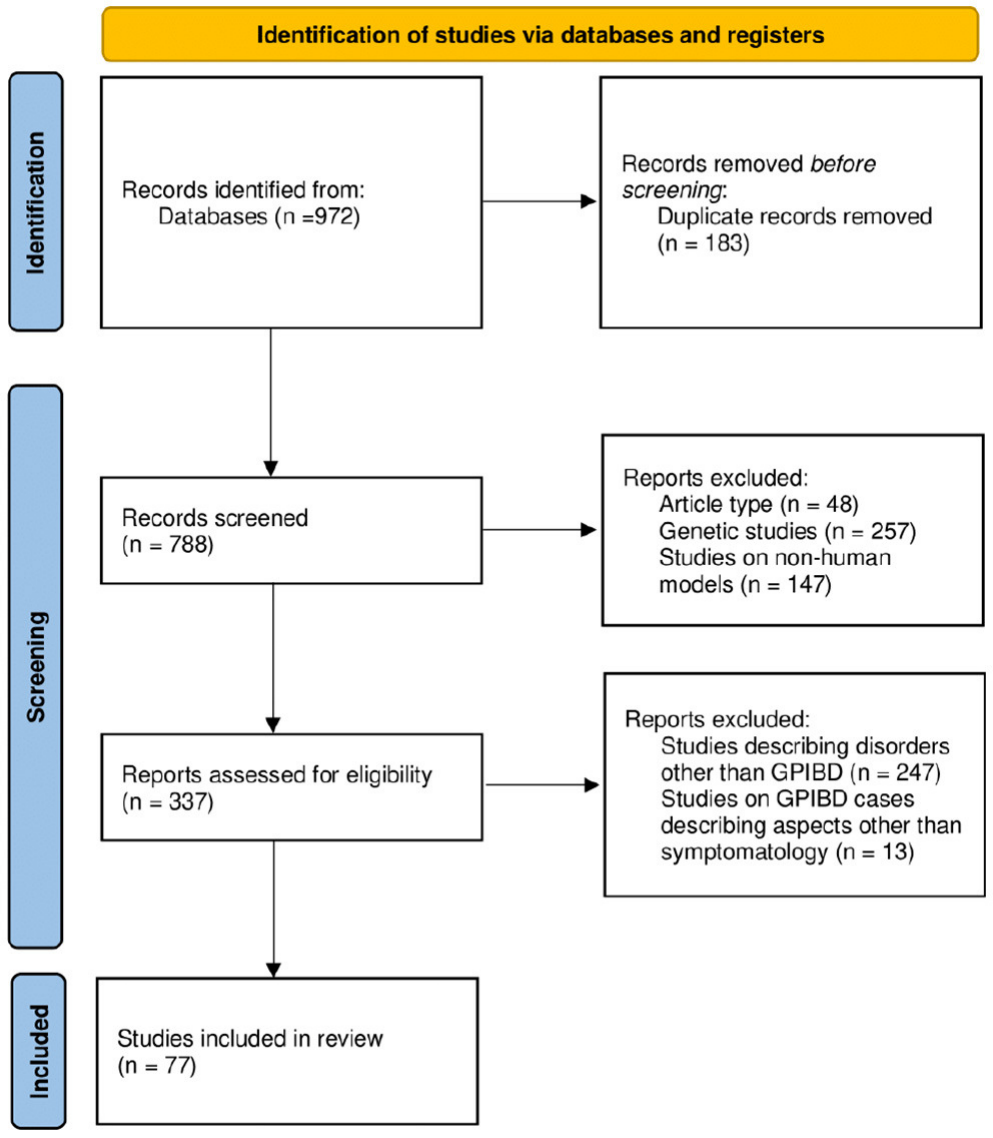
Flow diagram of the data collection.

After exclusion of review articles, genetic examinations, and studies on animals, the reports were assessed for eligibility, ruling out studies describing disorders other than GPIBD or issues other than neurological symptoms of GPIBD by two independent reviewers (MH and JH). The process of database browsing was presented using PRISMA flow diagram in Figure 1.

### Data Collection Process

From the included reports the data was collected by seven reviewers (JP, AT, MK, KS, AS, AJ-S, and RŚ), working independently on the assigned reports, results of which were then collectively discussed. Raw data concerning symptomatology was summarized in a table, presenting a quantity of patients presenting each neurological symptom in defect of each gene, as well as in the whole analyzed population.

Genes and syndromes terminology were collected from Online Mendelian Inheritance in Man (OMIM) database.

### Data Items

The pre-defined outcomes of the study—prevalence and onset of neurological symptoms—were developed to the full outcome measures based on the findings in the literature, and then divided to the primary and secondary outcome measures, based on their significance to the issue as a whole.

The main primary outcomes were the data of patients:

- prevalence of neurological symptoms (expressed as quantity of patients presenting with a given symptom)
- onset (age in time of first seizure, in months)
- response to antiepileptic treatment (quantity of patients with refractory seizures)
- semiology of epileptic seizures (quantity of seizure types in patients)
- onset of developmental delay (age in time of first observed developmental delay)

The secondary outcome of the review was the profile of the antiepileptic medicaments used in controlling the epilepsy in pediatric patients with GPIBD (quantity of each AED used in treatment).

Reports missing some of the outcome measures were not included in the analysis of the outcomes which were missing, although they were not excluded from the review as a whole.

### Study Risk of Bias Assessment

Risk of bias for each study was assessed using The Joanna Brigs Institute (JBI) Critical Appraisal Checklist for Case Reports (1), Case Series (1), or Prevalence Studies (2) by two independent reviewers (JP and MH). Calculation of risk of bias using JBI Critical Appraisal Checklist for Case Reports assessed: clarity of demographic characteristics, description of patient's history, current clinical condition, diagnostic tests, interventions, post-intervention clinical condition, identification of adversary affects, and inclusion of concluding remarks on the case. JBI Critical Appraisal Checklist for Case Series assessed: inclusion criteria in terms of criteria clarity, consecutiveness, and completeness of inclusion; reliability of condition identification and measurement in cases; reporting the demographics and clinic demographics; clinical and follow up information; and statistical analysis. JBI Critical Appraisal Checklist for Prevalence Studies assessed: response rate, sample frame, sample size, sampling efficacy, subject and setting description quality, sufficiency of data analysis, identification of condition, condition measures, and statistical analysis quality. As the used tools are dedicated for appraisal in terms of inclusion/exclusion of studies, they were modified by the reviewers to also assess the reports qualitatively to low, medium, and high risk of bias, based on the number of unmet criteria.

### Effect Measures

For the qualitative data of patients—prevalence of specific neurological symptoms and seizures of the specific semiology, drug-resistance of epilepsy, profile of antiepileptic medication—the measure was the quantity of patients presenting with the given symptom/condition or being subject to given treatment. For the onset developmental delay (both motor and intellectual) the measure was age in months at which the first sign of developmental delay was observed—if time of observation was not specified, an adequate time of first not achieved developmental milestone would be adapted.

### Synthesis Methods

The report selection and data collection processes were conducted as described in sections 2.5 and 2.6. Every study eligible for the synthesis is listed in Supplementary Table 1.

The data conversion from reports of cases to the quantitative data was conducted by seven reviewers (JP, AT, MK, KS, AS, AJ-S, and RŚ), working independently on the assigned reports.

Obtained data were then summarized for the purpose of synthesis into Table 1, presenting the quantity of each symptom in the groups of specific GPIBD-causative gene, as well as in the general population of GPIBD patients. The mean time of seizure onset was also visually displayed as a Figure 3. using boxplot tool of STATISTICA 13.3.721.1 (StatSoft Polska Sp. z.o.o.).

Table 1A summary of affected genes and symptoms present in patients of reviewed articles.

**Table:** 

**Gene**	***ARV1***	***DPM1***	***MPDU1***	***GPAA1***	***PGAP1***	***PGAP2***	***PGAP3***	***PIGA***	***PIGB***	***PIGG***
**(A)**										
Number of patients	11	9	9	10	6	16	30	81	14	7
Encephalopathy	11	0	0	0	2	0	0	3	0	0
Seizures	11	8	8	7	1	6	17	76	14	6
Delayed motor development	5	7	7	0	3	8	28	61	0	3
DD/ID	8	7	9	10	3	13	28	70	13	5
Hypotonia	11	7	8	10	3	5	25	55	0	4
Hypertonia/ spasticity	8	0	1	4	1	0	0	4	1	0
Dystonia	6	0	0	0	0	0	0	7	0	0
Dyskinesia	2	0	0	0	1	0	0	8	0	0
Dysphagia	7	2	2	0	0	0	1	0	0	0
Dysarthria	5	0	0	5	0	0	0	0	0	0
Cerebellar atrophy	3	3	4	9	0	0	3	19	0	0
Cerebellar dysfunction	3	1	1	8	0	0	0	1	0	4
Nystagmus	3	3	1	8	2	1	1	1	0	0
Strabismus	0	1	4	0	1	2	0	5	0	0
Visual impairment	8	5	2	0	2	0	0	17	8	0

**Table:** 

***PIGH***	***PIGK***	***PIGL***	***PIGN***	***PIGO***	***PIGP***	***PIGQ***	***PIGS***	***PIGT***	***PIGU***	***PIGV***	***PIGW***	***PIGY***	**Total**
**(B)**													
6	12	5	27	11	5	8	13	38	5	9	1	4	337
0	0	0	0	0	4	0	0	0	0	0	0	0	20
5	8	5	23	6	5	7	11	33	5	8	1	2	273
0	0	4	7	9	0	6	4	31	0	7	0	0	190
6	12	5	23	6	5	7	11	34	5	7	1	4	292
4	12	0	22	7	4	6	8	17	5	4	0	0	217
0	0	0	2	1	2	0	1	2	2	0	0	0	29
0	0	0	0	0	0	0	0	0	0	0	0	0	13
0	0	0	1	0	4	0	0	0	0	0	0	0	16
0	0	0	0	1	1	0	0	1	0	0	0	0	15
1	0	0	0	0	0	0	0	4	0	0	0	0	15
0	10	0	1	2	0	0	4	15	4	0	0	0	77
0	6	3	7	0	0	0	4	3	0	0	0	0	41
0	1	1	12	0	0	0	3	9	1	0	0	0	47
2	0	1	0	0	0	0	0	8	5	0	0	1	30
0	0	0	0	2	1	0	6	11	3	0	0	2	67

***(A)** Part I **(B)** Part II. DD/ID, developmental delay/intellectual disability*.

Statistical analysis was conducted using STATISTICA 13.3.721.1 (StatSoft Polska Sp. z.o.o.) by M.H. Depending on group size criterium, chi-square and Fisher's exact tests were used for assessing the statistical significance of differences in phenotype (symptoms and semiology of seizures) of patients. Mann-Whitney U test was used for comparing onset of intellectual and motor development delay. A significance threshold of *p* < 0.05 was applied for each test.

## Results

### Study Selection

Multiple browsing of databases was conducted over the span of November 2020–September 2021, returning a sum of 972 records, which, after removal of repetitions, was reduced to 788. Adequate filters and the inclusion and exclusion criteria were applied. A total of 337 cases reported in 77 articles was included for the purpose of the review, as shown below in Figure 1. A list of excluded studies can be found in Supplementary Table 2, divided according to the exclusion criteria (article type, genetic studies, studies on non-human subjects, studies describing disorders other than GPIBD, and studies on GPIBD cases describing aspects other than symptomatology).

### Study Characteristics

Included studies are listed in Supplementary Table 1, with descriptions of examined genes, number of examined patients (with their nationality), and the type of discovered mutations. Of 77 included studies, 29 were case reports (37.66%), 46 were case series studies (59.74%), and 2 were prevalence studies (2.60%). The oldest reported cases are from the year 2000, 51/77 (66.23%) studies were published in the last 5 years and 71/77 (92.20%) in the last 10 years. The data on the gender of patients was available for 289/337 cases (85.76%), of which 120 were females and 169 were males.

### Risk of Bias in Studies

Included studies were then assessed for risk of bias using the JBI Critical Appraisal Checklist for Case Reports, for Case Series, and for Prevalence Studies by two independent reviewers (JP and MH). To increase the precision of the tool, the analysis was quantified—each study was assessed in terms of tool items according to the included instructions (“yes,” “no,” “unclear,” or “not applicable”), each answer to the tool item was translated according to the pattern: “yes” = 2, “no” = 0, “unclear” = 1, and “not applicable”—tool item not used for calculation. The general score of the study was then calculated as follows:


$$\begin{array}{l} \frac{Sum\;of\;points\;awarded\;in\;each\;tool\;item}{Number\;of\;assessed\;item^{*}2}*100\% \end{array}$$

**Figure 2:**
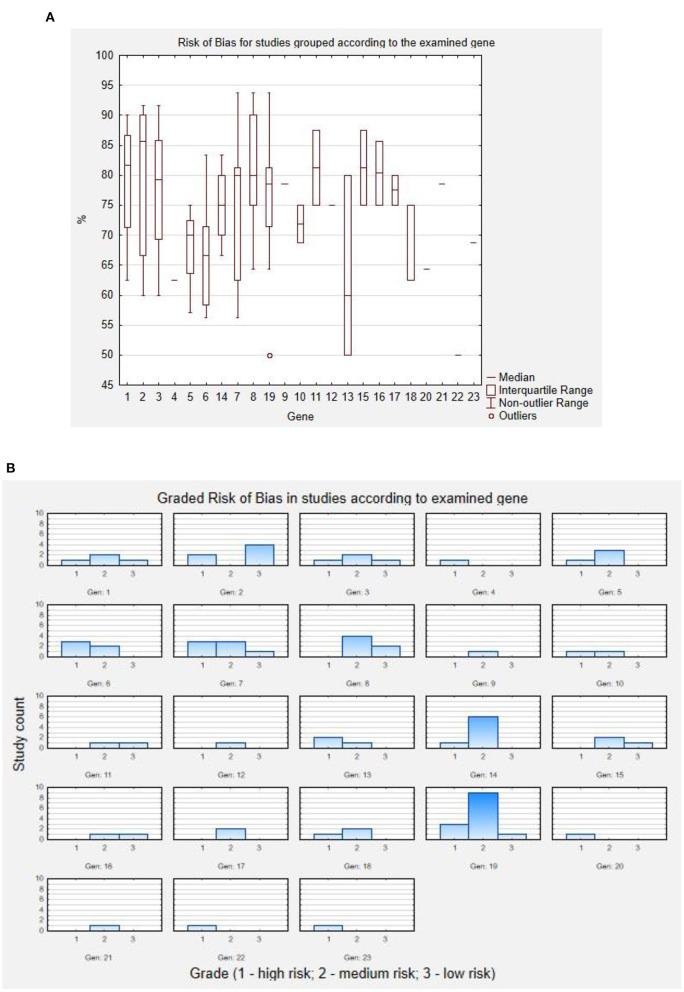
Results of risk of bias assessment. **(A)** Risk of bias for studies grouped according to the examined gene. **(B)** Graded risk of bias in studies according to examined gene. Codes for genes: 1 - *ARV1*; 2 - *DPM1*; 3 - *MPDU1*; 4 - *GPAA1*; 5 - *PGAP1*; 6 - *PGAP2*; 7 - *PGAP3*; 8 - *PIGA*; 9 - *PIGB*; 10 - *PIGG*; 11 - *PIGH*; 12 - *PIGK*; 13 - *PIGL*; 14 - *PIGN*; 15 - *PIGO*; 16 - *PIGP*; 17 - *PIGQ*; 18 - *PIGS*; 19 - *PIGT*; 20 - *PIGU*; 21 - *PIGV*; 22 - *PIGW*; 23 - *PIGY*.

The obtained result is the percentage of acquired point from the maximal possible score in each assessment. These results were then translated to “high,” “medium,” and “low” risk of bias, using the following rule: studies scoring <70%—“high” risk of bias; studies scoring 70–85%—“medium” risk of bias; and studies scoring >85%—“low” risk of bias. The results of this assessment are presented below in Figure 2. As a boxplot of studies' assessment scores in percent according to the studied gene (Figure 2A), or as the graded risk of bias score according to the examined gene (Figure 2B).

### Results of Individual Studies

The spectrum of symptoms involved encephalopathy, delayed motor development, developmental delay/intellectual disability (DD/ID), visual impairment, hypotonia, hypertonia/spasticity, dystonia, dyskinesia, seizures, cerebellar atrophy, nystagmus, cerebellar dysfunction, strabismus, dysphagia, and dysarthria. A summary of affected GPI-AP synthesis genes and caused symptoms is presented below in Table 1.

### Results of Syntheses

Presented data give an insight into the general spectrum of symptoms in GPIBDs. The most prevalent is developmental delay, both motor (195/337) and intellectual (295/337), often presenting simultaneously, followed by a huge variety of seizure types (273/337) and sporadic encephalopathy (20/337). Another group of symptoms revolve around the muscle tone abnormalities, of which more -frequently present is hypotonia (217/337), with a few cases of hypertonia or spasticity (29/337), dyskinesia (16/337), and dystonia (13/337). The remaining symptoms can be ordered into cerebellar: atrophy (77/337), dysfunction (41/337), nystagmus (47/337); and ophthalmological: strabismus (30/337) and visual impairment (67/337). The latter of these symptoms often stems from the optic nerve atrophy. The significant differences in frequency of symptoms between groups of patients with mutations in different genes arepresented below in Table 2.

**Table 2:** Differences in frequency of symptoms in patients with GPI biosynthesis gene mutations.

**Symptom**	**Gene**	**Change**	***p-*value**
**(A)**			
Encephalopathy	*ARV1*	UP	<0.0001
Encephalopathy	*PIGP*	UP	0.0001
Seizures	*PGAP1*	DOWN	<0.002
Seizures	*PGAP2*	DOWN	0.0001^*^
Seizures	*PGAP3*	DOWN	<0.002^**^
Seizures	*PIGA*	UP	<0.005
Seizures	*PIGO*	DOWN	<0.05
DMD	*GPAA1*	DOWN	<0.0005
DMD	*PGAP3*	UP	<0.0001
DMD	*PIGA*	UP	<0.002^**^
DMD	*PIGB*	DOWN	<0.0001
DMD	*PIGH*	DOWN	<0.01
DMD	*PIGK*	DOWN	0.0001
DMD	*PIGN*	DOWN	<0.005^*^
DMD	*PIGP*	DOWN	<0.02
DMD	*PIGT*	UP	<0.005^*^
DMD	*PIGU*	DOWN	<0.02
DMD	*PIGY*	DOWN	<0.05
DD/ID	*PGAP1*	DOWN	<0.05
DD/ID	*PIGO*	DOWN	<0.02
Hypotonia	*ARV1*	UP	<0.02
Hypotonia	*GPAA1*	UP	<0.02
Hypotonia	*PGAP2*	DOWN	<0.02
Hypotonia	*PGAP3*	UP	<0.05
Hypotonia	*PIGB*	DOWN	<0.0001
Hypotonia	*PIGK*	UP	0.01
Hypotonia	*PIGL*	DOWN	<0.01
Hypotonia	*PIGT*	DOWN	<0.02^**^
Hypotonia	*PIGY*	DOWN	<0.02
Hypertonia	*ARV!*	UP	<0.0001
Hypertonia	*GPAA1*	UP	<0.01
**(B)**			
Dystonia	*ARV1*	UP	<0.0001
Dyskinesia	*PIGP*	UP	<0.0001
Dysphagia	*ARV1*	UP	<0.0001
Dysarthria	*ARV1*	UP	0.0001
Dysarthria	*GPAA1*	UP	0.0001
Cerebellar atrophy	*GPAA1*	UP	<0.0001
Cerebellar atrophy	*PGAP2*	DOWN	<0.05
Cerebellar atrophy	*PIGB*	DOWN	<0.05
Cerebellar atrophy	*PIGK*	UP	<0.0001
Cerebellar atrophy	*PIGN*	DOWN	<0.02
Cerebellar atrophy	*PIGT*	UP	<0.05^**^
Cerebellar atrophy	*PIGU*	UP	<0.02
Cerebellar dysfunction	*GPAA1*	UP	<0.0001
Cerebellar dysfunction	*PGAP3*	DOWN	<0.05
Cerebellar dysfunction	*PIGA*	DOWN	<0.002
Cerebellar dysfunction	*PIGG*	UP	<0.01
Cerebellar dysfunction	*PIGK*	UP	<0.001^*^
Cerebellar dysfunction	*PIGL*	UP	<0.02
Nystagmus	*GPAA1*	UP	<0.0001
Nystagmus	*PIGA*	DOWN	<0.0005
Nystagmus	*PIGN*	UP	<0.0001^**^
Strabismus	*MPDU1*	UP	<0.01
Strabismus	*PIGT*	UP	<0.05^*^
Strabismus	*PIGU*	UP	<0.0001
Visual impairment	*ARV1*	UP	<0.001
Visual impairment	*DPM1*	UP	<0.05
Visual impairment	*PGAP2*	DOWN	<0.05
Visual impairment	*PGAP3*	DOWN	<0.005
Visual impairment	*PIGB*	UP	<0.005^*^
Visual impairment	*PIGN*	DOWN	<0.01

***(A)** Part I **(B)** Part II. P-value for Fisher's exact test, unless marked with*

“*” *(chi-square test with Yates's correction) or*

“**” *(chi-square test). UP–frequency higher than in general population of study. DOWN–frequency lower than in general population of study. DMD, delayed motor development; DD/ID, development delay/intellectual disability*.

Seizures were present in 273 patients, with significant difference in frequency between generalized (93/273) and focal (123/273) (*p* < 0.01) or unknown (119/273) (*p* < 0.05) onset types of seizures, with lack of such a difference between the latter. A huge (*p* < 0.0001) dominance of motor over non-motor semiology of seizures was observed in epileptic events of focal and generalized onset. The two most frequent types of focal motor seizures were myoclonic (41/77) and epileptic spasms (17/77), with a noticeable number of patients experiencing evolution of seizures from focal or multifocal to bilateral tonic-clonic seizures. One hundred and six patients suffered from intractable focal (57/126), generalized (36/93), and unknown (35/110) onset seizures, however they were significantly (*p* < 0.0001) less frequent than non-intractable seizures. The most commonly applied anti-epileptic drugs (AEDs) were valproic acid (*N* = 60), levetiracetam (*N* = 50), and topiramate (*N* = 30). Less frequently used were phenobarbital, clobazam, carbamazepine, clonazepam, and pyridoxine. Symptomatic treatment of epilepsy is the only approach, and there are no effective antiepileptic drugs. Supplementation in vitamin B6 (20–30 mg/kg) in HPMRS could improve electroencephalography or reduce seizure frequency as ALP plays a role in the synthesis of vitamin B6. Based on some case reports, ketone diet is effective in children with a PIGA gene mutation (3).

**Figure 3:**
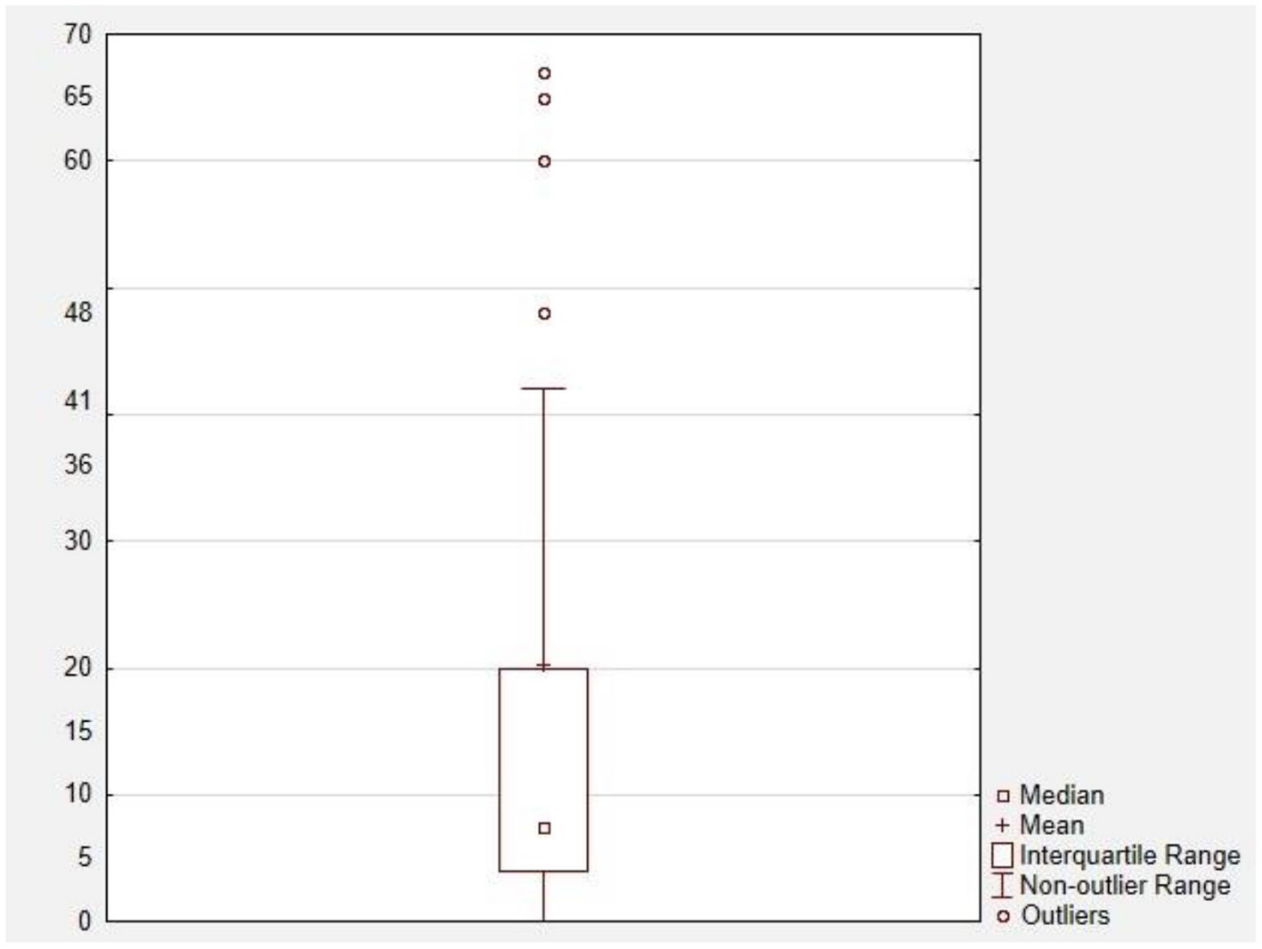
Boxplot of time of first seizure in patients of this study [months].

Although data concerning time of first epileptic seizure was not available for every studied patient, collected information shows variety (Min.: 0.03 months; Max.: 276 months; Median: 7 months) in this matter, with mean time of c.a. 18.32 months. Adequate boxplot is presented below in Figure 3. Due to the very high values of extreme outliers, they were not marked in this graph in order to avoid confusion.

Looking at the EEG description in GPIBDs, the most frequently seen abnormality was multifocal or focal epileptic activity with or without very disorganized background.

Data concerning the onset of developmental delay, both motor and intellectual, was not available for all of the patients (132/195 and 51/295, respectively), which is an important limitation of the study. Mean time of first observed sign of developmental delay for ID was 6.85 months and 6.82 for DMD and did not differ significantly (*p* = 0.67; Mann-Whitney test). Intellectual delay varied in severity, presenting as mild (16 patients), moderate/severe (113 patients), or profound (109 patients). Reported children achieved different milestones of motor development, although it frequently did not progress further than the ability to control head movements, roll both ways, or attempt to walk.

An in-depth description of studies included for each gene group is presented in the following subunits.

#### ARV1 (Fatty Acid Homeostasis Modulator, MIM 611647)

*ARV1* is a well-established gene among eukaryotic species, whose product is a transmembrane protein in endoplasmic reticulum (ER), serving as a transporter for lipid molecules. Its exact mechanism is not yet evident, although the prevalence of this gene in different organisms, as well as its common expression in most tissues, highly suggest its importance. It is suggested that it might also take part in GPI anchor biosynthesis at the very early stages of the process, as it was proven in yeast.

This gene is not widely linked to GPIBD, but two patients affected by *ARV1* gene mutations presented a clinical spectrum very similar to the typical GPIBD phenotype. In 2016 a case of homozygous in-frame truncating mutation of *ARV1* in a 12-month-old deceased girl was presented. Over the 12 months of her life, she was diagnosed with encephalopathy movement disorders like central hypotonia, peripheral hypertonia, extensor posturing, and dystonia. She also experienced status epilepticus, which turned into multifocal seizure disorder. Mutation of *ARV1* gene caused symptomatic disorder resembling early infantile epileptic encephalopathy, as further proved in cases of two sisters with biallelic mutation of *ARV1*, who also presented with encephalopathy and migrating focal seizures (4, 5). Another known case of *ARV1* was a 5-year-old (at the time of the study) boy, who presented with a very similar phenotype of DD, epilepsy of unknown onset, and motor and speech developmental delay (6). Seven patients who shared a consistent phenotype of encephalopathy, seizures, global developmental delay, central hypotonia with peripheral hypertonia, and different degrees of cerebellar and ophthalmological disorders were analyzed in terms of GPI biosynthesis. All of them harbored the *ARV1* mutations, which were found to result in loss of GPI-anchored proteins on neutrophils and fibroblasts. These results suggest that *ARV1* mutations lead to GPIBD (7).

#### DPM1 (Dolichyl-Phosphate Mannosyltransferase 1, MIM 603503)

The product of this gene is engaged, along with other *DPM*- genes (-*2*,−*3*), in dolichol-P-mannose (DPM) synthase. DPM is in turn a substrate of numerous glycosylation reactions, including GPI-anchor synthesis, and as such its mutations impairing the DPM synthase function lead to diseases jointly called congenital disorders of glycosylation (CDGs).

A novel phenotype of CDG1E (MIM 608799; 20q13.13) has been recently proposed, based on a boy primarily diagnosed with congenital muscular dystrophy, due to his clinical features—developmental delay and severe hypotonia, which led to the prescription of leg braces. He developed later epileptiform activity, amounting to grand-mal seizures. Although the GPI-anchor synthesis was not evaluated, it is also suggested to be influenced by mutation of *DPM1*, based on the alteration of CD59 expression in fibroblasts (8). CDG1E was first described in 2000 in two children, who both showed severe developmental delay, hypotonia, and seizures (generalized; unknown) (9). A similar phenotype of seizures and hypotonia was identified in the same year in two other cases with DPM1 mutations (10). On the contrary, two subjects of the 2004 study (11) and one of 2006 (12) study displayed milder symptoms, with a larger share of cerebellar dysfunctions (ataxia), which were not present in other described cases. Seizures were both focal (atonic, tonic, clonic, and myoclonic) as well as generalized (including absence). Non-neurological symptoms were more prominent in some patients, specifically gastrointestinal (with focal myoclonic and tonic seizures) (13). A detailed summary of symptoms in the above-mentioned articles can be found below in Table 3.

**Table 3:** Articles presenting patients with *DPM1* mutations.

	**Yang et al. (8)**	**Kim et al. (9)**	**Imbach et al. (10)**	**Garcia-Silva et al. (11)**	**Dancourt et al. (12)**	**Bursle et al. (13)**
Number of patients	1	2	2	2	1	2
Seizures	1	2	2	0	1	1
Delayed motor development	1	0	2	1	2	1
DD/ID	1	0	2	1	2	1
Hypotonia	1	2	2	0	2	0
Dysphagia	0	0	0	1	1	0
Cerebellar atrophy	0	1	0	0	2	0
Cerebellar dysfunction	0	0	0	1	0	0
Nystagmus	0	0	0	1	2	0
Strabismus	0	0	0	0	1	0
Visual impairment	0	2	0	1	2	0

*DD/ID, developmental delay/intellectual disability*.

#### MPDU1 (Mannose-P-Dolichol Utilization Defect 1, MIM 604041)

In 2001 a total of three unrelated patients were examined through primary fibroblasts cultivation and lipid-linked oligosaccharide (LLO) analysis, with further genomic polymerase chain reaction (PCR) assessing the structure and expression of *Lec35/MPDU1* gene. LLO profile found in these patients was distinctive from profiles reported in the literature beforehand. Despite the limited number of patients, they differed in type of mutation and severity of symptoms, which seemed to be conjugated with each other. Patients presented with varied intensity and extensity of seizure activity and DD/ID. Two of them were hypotonic, whereas the other one was hypertonic. The severely hypotonic subject also suffered from optic atrophy. Due to similar clinical image and common location of mutation, studied children were the first ones diagnosed with CDG1F (MIM 609180; 17p13.1) (14), which was followed in the same year with a diagnosis of another CDG1F case with more pronounced cerebellar symptoms (15). Recently five more CDG1F patients of Arabic origin were reported, presenting a uniform set of symptoms, reminiscent of ones shown in both previous studies (16, 17). Most of the CDG1F patients presented generalized onset motor seizures (14–17), however one of them experienced only focal tonic and clonic seizures (16).

#### GPAA1 (Glycosylphosphatidylinositol Anchor Attachment Protein 1, MIM 603048)

In 2017 a total of 10 patients with biallelic frameshift, intronic splicing, and missense mutations in *GPAA1* gene were described. The product of this gene plays an important role in GPI-AP biosynthesis, as part of the transamidase complex. The main clinical components included DD/ID, hypotonia, cerebellar atrophy, nystagmus, and seizures. Dysarthria was found in half of the patients. All of them showed generalized onset motor seizures, of which 90% were tonic-clonic, whereas focal motor seizures were present in 4 out of 10 patients, with a predominantly myoclonic component. On the grounds of genetic alterations and presented symptoms, each patient was diagnosed with GPIBD15 (MIM 617810; 8q24.3) (18).

#### PGAP Gene Family

##### PGAP1 (Post-GPI Attachment to Proteins 1, MIM 611655)

Despite the small number of patients with identified mutations in the *PGAP1* gene, the spectrum of symptoms presented was quite non-uniform. In the earliest described cases of *PGAP1*-linked GPIBD9 (MIM 615802; 2q33.1), intellectual and motor development was delayed in both related subjects, with the additional presence of the absence seizures in one of them (19). Another GPIBD9 patient, unrelated to those previously described, also presented with general development delay, but with simultaneous visual impairment and occurrence of dyskinesias (20). In either situation, a mutation of the *PGAP1* gene was a loss-of-function one. Interestingly, a 2015 study identified truncating splice mutations in two cases of GPIBD9, which differed in symptoms: encephalopathy and hypotonia (in one case preceded by hypertonia) (21). Milder symptoms included visual impairment, nystagmus, strabismus, and hypotonia which were present in a patient with deleterious mutations of *PGAP1*, which resulted in abnormal structure of GPI-APs (22).

##### PGAP2 (Post-GPI Attachment to Proteins 2; MIM 615187)

Three missense mutations were found in two unrelated patients in the *PGAP2* gene, which led to a diagnosis of GPIBD8 (MIM 614207; 11p15.4). Both cases presented with delayed motor development and grand mal epilepsy. In one of them hypotonia and generalized myoclonic seizures were also observed (23). These symptoms were common in other cases of GPIBD8, additionally complemented with DD, nystagmus and epileptic (24), as well as with poor hearing (25). A very rare single nucleotide polymorphism (SNP) variant in *PGAP2* caused a surprisingly distinct phenotype, consisting of isolated DD (26). Similarly in seven patients with homozygous mutations in *PGAP2* gene, the main findings were intellectual disability, present in 6/7 patients, with less than half of them presenting with hypotonia and DMD. Interestingly, another distinct feature was elevated ALP activity (5× times) (27).

##### PGAP3 (Post-GPI Attachment to Proteins 3; MIM 611801)

Five patients, of which three are related, were examined in terms of the genetic background of presented disorders, consisting of delayed intellectual and motor development, hypotonia, and in 4/5 patients, seizures (2 grand mal, 1 unknown onset tonic-clonic, and 1 focal myoclonic). Results showed four different mutations of the *PGAP3* gene, which are linked to GPIBD10 (MIM 615716; 17q12) (28). Over the years more patients were diagnosed with this disorder, accounting for 30 total cases as of today. Seizures, DD, delayed motor development, and hypotonia remained the main symptoms, with singular cases presenting dysphagia, cerebellar atrophy, and nystagmus. Focal seizures were always myoclonic, whereas generalized onset seizures were in 67% tonic-clonic, in 22% myoclonic, and 3 of the patients presented 3 unknown onset epilepsy (one tonic-clonic). The summary of symptoms is given below in Table 4 (28–34).

**Table 4:** Articles presenting patients with *PGAP3* mutations.

	**Howard et al. (28)**	**Knaus et al. (29)**	**Nampoothiri et al. (30)**	**Abdel-Hamid et al. (31)**	**Sakaguchi et al. (32)**	**Da'as et al. (33)**	**Bezuidenhout et al. (34)**
Number of patients	5	8	2	10	1	1	3
Seizures	4	6	0	5	1	0	1
Delayed motor development	5	8	1	10	1	1	2
DD/ID	5	8	1	10	1	1	2
Hypotonia	5	4	1	10	1	1	3
Dysphagia	0	0	0	0	0	1	0
Cerebellar atrophy	0	0	0	3	0	0	0
Nystagmus	0	0	0	1	0	0	0

*DD/ID, developmental delay/intellectual disability*.

#### PIG Gene Family

This gene family encodes a series of proteins involved in the synthesis of GPI anchor biosynthesis. As such it is natural that patients with identified mutations in *PIG* genes are likely to present symptoms associated with disorders of glycosylation. Patients presenting with the aforementioned mutations are described in the section below.

##### PIGA (Phosphatidylinositol Glycan Anchor Biosynthesis Class A Protein; MIM 311770)

Association of *PIGA* gene with GPIBD, namely GPIBD4 (MIM 300868; Xp22.2), is a discovery of the last 10 years, as it was mainly connected with paroxysmal nocturnal hemoglobinuria (MIM 300818; Xp22.2). A total of 81 GPIBD4 patients presented with varied symptomatology, which consisted of intellectual (70/81) and motor (61/81) development delay and hypotonia (55/81). In individual cases encephalopathy, dystonia, dyskinesia nystagmus, strabismus, and visual impairment were also observed (35–38). Among these patients, 76 were diagnosed with epilepsy, of which 52 were drug-resistant. Less than a half of subjects experienced focal seizures (36/76), specifically epileptic spasms (10/36), atonic (3/36), and myoclonic (2/36) seizures. Moreover, 11 patients presented evolution from focal to bilateral tonic-clonic seizures. Generalized onset seizures were observed in 30 patients–29 of them experienced tonic-clonic seizure, 11 experienced generalized myoclonic seizure, and eight of them experienced absence seizure (3, 35, 38, 39). Further analysis of patients with *PIGA* mutations confirmed the above clinical image, with predominant prevalence of development delay, hypotonia, and seizures (3). In some patients *PIGA* mutations also resulted in symptoms other than neurological—skin lesions and renal and hepatic iron storage were also observed (39).

##### PIGB (Phosphatidylinositol Glycan Anchor Biosynthesis Class B Protein; MIM 604122)

A recent study described 14 patients from 10 unrelated families examined in terms of genetic alterations causing their condition, with results showing that the causative gene mutations are linked with the *PIGB* gene. Some patients shared the symptoms with deafness, onychodystrophy, osteodystrophy, intellectual delay, and seizure syndrome (DOORS; MIM 220500; 16p13.3), which stems from alterations in the *TBC1D24* gene (MIM 613577). Due to different genetical backgrounds, all the patients were diagnosed with GPIBD20 (MIM 618580). All patients experienced seizures, although their precise details were not reported. Moreover, all but one subject presented with ID, in 57% of patients visual impairment was observed, and one patient also showed spastic hypertonia (40).

##### PIGG (Phosphatidylinositol Glycan Anchor Biosynthesis Class G Protein; MIM 616918)

Although a study from 2016 did not explain the etiology of symptoms in patients with dysfunctional *PIGG* gene, due to the lack of alterations in GPI-APs structure and surface levels in B lymphoblasts (41), a more recent examination showed that these changes could be observed in fibroblasts of these patients, providing an insight into the molecular background of a condition known as GPIBD13 (MIM 616917; 4p16.3) (42). The spectrum of presented disorders is comparable between both studies, with differences in frequency: seizures (100 vs. 80%), ID (100 vs. 60%, hypotonia (100 vs. 40%), and cerebellar dysfunction (100 vs. 40%). There are also differences in the presence of delayed motor development in 3/5 patients of the more recent study. On the other hand, classification of seizures was more diverse, as one patient presented a wide range of focal seizures: tonic and autonomic with impaired awareness. The same patient experienced grand mal seizures. The other 3 patients also had generalized onset seizures, of which one is absence type. They and two other patients also showed unknown onset seizures, of which two were tonic-clonic. First epileptic episode appeared at a mean of 5 months, and was followed by drug-resistant epilepsy in two patients with generalized onset (41, 42).

##### PIGH (Phosphatidylinositol Glycan Anchor Biosynthesis Class H Protein; MIM 600154)

A pair of siblings affected by the same homozygous truncating mutation of the *PIGH* gene was recently described. *PIGH* is a member of a *PIG* gene family, associated with GPIBD17 (MIM 618010; 14q24.1). Children presented the exact phenotype of DD/ID and seizures, with the only difference in the semiology of epileptic seizures—one experienced tonic-clonic, whereas the other myoclonic episodes. Both children also showed febrile seizures. Recently four more cases of *PIGH* mutations were reported, which differed in location of mutation (Arg163 instead of Ser130). It was reflected by different expression levels of CD16 on the surface of granulocytes. Newly described children had similar symptoms to the previous patients, but also presented with hypotonia (4/4), strabismus (2/4), and dysarthria (1/4). The average time of onset was c.a. 18 months (43, 44).

##### PIGK (Phosphatidylinositol Glycan Anchor Biosynthesis Class K Protein; MIM 605087)

The presence of biallelic mutations in the *PIGK* gene proved to directly result in alterations of GPI-Aps levels, which lead to the development of GPIBD22 (MIM 618879; 1p31.1). Twelve described patients presented a varied symptomatology of this condition, consisting in all cases (12/12) of DD/ID, hypotonia, and less frequently of cerebellar atrophy (10/12), cerebellar dysfunction (6/12), and nystagmus (1/12). Epilepsy was observed in 8 patients, of which 3 presented with focal seizures, both motor (automatisms, atonia, clonic movements, myoclonia) and non-motor (behavior arrest, awareness impairment); 4 presented with generalized onset, of which 3 experienced grand mal seizures and one atonic episode. In 2 patients the unknown onset seizures were present, including one febrile (45).

##### PIGL (Phosphatidylinositol Glycan Anchor Biosynthesis Class L Protein; MIM 605947)

GPIBD5 (MIM 605947; 17p11.2), also known as CHIME syndrome due to its most recurrent symptoms, is a disorder caused by the *PIGL* gene. Every patient presented with epileptic seizures, of which the most frequent were myoclonic (4/5), followed by tonic (2/5) and epileptic automatisms (1/5). In one of those patients the seizures evolved from focal to bilateral tonic-clonic. Apart from patients with focal onset there was also one with unknown onset of seizures. Other symptoms identified in presented GPIBD5 included intellectual (5/5) and motor (4/5) development delay, cerebellar atrophy (3/5), nystagmus, and strabismus (1/5; in the same subject) (46–48).

##### PIGN (Phosphatidylinositol Glycan Anchor Biosynthesis Class N Protein; MIM 606097)

Patients with mutations in the *PIGN* gene described in the available literature presented a set of symptoms that each appeared in over 80% of GPIBD3 (MIM 606097; 18q21.33) patients–DD/ID, hypotonia, and seizures. Less frequent were nystagmus (44%), cerebellar atrophy, delayed motor development (each 26%), and least frequently hypertonia with spasticity (7%). Thirteen out of 23 epileptic patients lacked details concerning seizure type. Of these patients, 8 presented with focal seizures (3 myoclonic, of which one evolved into a bilateral tonic-clonic; 1 clonic) and 4 generalized (1 tonic-clonic; 3 atypical absence). Interestingly many patients (7/23) who experienced febrile seizures and epilepsy in most cases (19/23) were drug-resistant. The mean time of the first seizure was 6.9 months. Mutation in *PIGN* gene were found in two fetuses, who developed cystic hygroma and other malformations, such as tetralogy of Fallot (24, 38, 49–53).

##### PIGO (Phosphatidylinositol Glycan Anchor Biosynthesis Class O Protein; MIM 614730)

A small number of patients were diagnosed with GPIBD6 (MIM 614749; 9p13.3), which results from alterations in *PIGO* gene. A total of 11 patients presented features of a typical GPIBD phenotype, but with a visible variety in severity of symptoms. The most prevalent among children of this group were global development delay (motor 9/11; intellectual 6/11), hypotonia (7/11), and seizures (6/11) with individual cases of cerebellar atrophy, visual impairment, spasticity, and dysphagia. These patients also presented a wide spectrum of seizures, both focal, including motor (spasms) and nonmotor (cognitive, emotional), and also tonic-clonic seizures of generalized and unknown onset. Each patient's first episode appeared in the first 2 years of life, with an average onset time of 13.6 months (54–56).

##### PIGP (Phosphatidylinositol Glycan Anchor Biosynthesis Class P Protein; MIM 605938)

Recent studies presented a total of 5 patients in whom mutations of the *PIGP* gene were present, resulting in GPIBD14 (MIM 605938; 21q22.13). The phenotype in these subjects was uniform, consisting of typical GPIBD symptoms, such as DD/ID (5/5), seizures (5/5), hypotonia (4/5), and encephalopathy (4/5), but also more distinctive symptoms, like dyskinesia (4/5), dysphagia (1/5), and visual impairment (1/7). It is worth noting that dyskinesia cases in this population make up 25% of dyskinesia in the general population of this study. Four of the patients presented focal seizures, of which 3 were epileptic spasms and 1 tonic episode. Two patients also experienced generalized seizures. The average time of onset was 3 months (57, 58).

##### PIGQ (Phosphatidylinositol Glycan Anchor Biosynthesis Class Q Protein; MIM 605754)

Clinical image of patients diagnosed with GPIBD19 (MIM 618548, 16p13.3), a disorder associated with alterations of PIGQ function, did not show any unusual symptoms. Seizures and DD/ID were present in 87.5% of patients, whereas delayed motor development and hypotonia were present in 75%. Seizures were mostly (5/7) focal motor, of which 2 were myoclonic, one was automatism episode, and one epileptic spasms. One non-motor focal seizure was observed (behavior arrest). Two patients experienced generalized petit mal seizures (59, 60). Patients presented with a first epileptic episode in a mean time of 4.78 months.

##### PIGS (Phosphatidylinositol Glycan Anchor Biosynthesis Class S Protein; MIM 610271)

Unlike in cases of *PIGQ*, patients with identified mutations in the *PIGS* gene showed not only typical symptoms of GPIBD, such as seizures, intellectual development delay (11/13), and hypotonia (8/13), but also a significant number of less frequent disorders—visual impairment (6/13), cerebellar dysfunction or atrophy (4/13), and nystagmus (3/13), hinting at a more complex image of GPIBD18 (MIM 618143; 17q11.2). Patients presented very early onset of seizures, which had either unknown onset (5/11) or focal (6/11). In the latter case the progression from focal or multifocal seizures to bilateral tonic-clonic seizures (4/6), as well as drug-resistance, were common (6/6). The onset time was low in the whole group, with average of circa 1.5 month (61–63).

##### PIGT (Phosphatidylinositol Glycan Anchor Biosynthesis Class T Protein; MIM 605754)

To date, 38 individuals with PIGT homozygous or compound heterozygous variants have been reported in the literature [summarized by Jiao et al. (38) and Wu et al. (64)], which were then jointly classified as GPIBD7 (MIM 615398; 20q13.12) (65–67). The clinical image of these patients was non-uniform, although more than 80% of patients presented with ID and seizures. Visual impairment was commonly observed in this study population. The summary of symptoms is presented in Table 5.

Table 5Articles presenting symptomatic image of patients with *PIGT* mutations.

**Table:** 

**Article**	**Jiao et al. (38)**	**Wu et al. (64)**	**Larsen et al. (65)**	**Jezela-Stanek et al. (66)**	**Lam et al. (68)**	**Skauli et al. (69)**
**(A)**						
Number of patients	2	1	1	7	2	2
Seizures	2	0	1	7	2	2
Delayed motor development	2	1	0	7	0	2
DD/ID	2	0	0	5	2	2
Hypotonia	1	0	1	7	2	2
Hypertonia/ spasticity	0	0	0	0	0	2
Dysphagia	0	0	0	0	0	0
Dysarthria	0	0	0	0	0	0
Cerebellar atrophy	0	0	0	3	2	2
Cerebellar dysfunction	0	0	0	0	0	2
Nystagmus	0	0	1	4	2	0
Strabismus	0	0	1	5	2	0
Visual impairment	0	1	1	0	2	2

**Table:** 

**Article**	**Nakashima et al**. **(****70****)**	**Kohashi et al**. **(****71****)**	**Pagnamaneta et al**. **(****72****)**	**Yang et al**. **(****73****)**	**Mason et al**. **(****74****)**	**Bayat et al**. **(****75****)**	**Chandar et al**. **(****76****)**
**(B)**							
Number of patients	1	1	3	1	1	15	1
Seizures	1	1	3	1	0	12	1
Delayed motor development	1	1	0	0	1	15	1
DD/ID	1	1	3	1	1	15	1
Hypotonia	1	1	0	0	1	0	1
Hypertonia/ spasticity	0	0	0	0	0	0	0
Dysphagia	0	1	0	0	0	0	0
Dysarthria	0	0	0	0	0	4	0
Cerebellar atrophy	1	1	1	0	0	5	0
Cerebellar dysfunction	0	0	1	0	0	0	0
Nystagmus	0	0	1	0	0	0	1
Strabismus	0	0	0	0	0	0	0
Visual impairment	0	1	3	0	1	0	0

***(A)** Part I **(B)** Part II. DD/ID, developmental delay/intellectual disability*.

The high variety was also observed in the classification of seizures. The highest number of patients experienced focal seizures: myoclonic (7/21), tonic (5/21), and hyperkinetic (1/21). Moreover, in 11 patients, focal epilepsy evolved into bilateral tonic-clonic. Tonic-clonic seizures had generalized onset in two patients, as well as unknown onset in six other patients. The first epileptic episode occurred at average 14 months, however some of the seizures had infantile onset. A few of the epileptic cases in this population were drug-resistant (8/33) (38, 64–66, 68–76).

Recently, Wu et al. (64) expanded the clinical spectrum of MCAHS3 to include bilateral anterior segment dysgenesis, avascular retinal periphery, and tractional retinal detachment.

Jiao et al. (38) gave the first report of a patient harboring three mutations in the *PIGT* gene-c.469 T > G(p.F157V) and c.1579_1581delinsCAT(N527H) paternally inherited, and c.1120A > G(p.N374D) of maternal origin. The proband is a boy with a motor development severely delayed since the onset of seizure (the first seizures occurred at the age of 3 during fever, then afebrile episodes occurred at the age of 9). Notably, his intelligence and language are normal. Moreover, no facial dysmorphism and congenital anomalies were noted. It means that the patient 16 reported by Jiao et al. is, to date, the only PIGT-CDG patient with neither facial nor other organ defects with the latest seizure onset age.

Otherwise, as described by other authors Bayat et al. (75), epilepsy is one of the cardinal features of the disorder, with myoclonic seizure being the most common seizure type in PIGT-GPIBDs patients. Moreover, focal seizures and epileptic spasms also occurred. In regards to treatment, drug resistance is often observed; thus, PIGT patients need polypharmacotherapy (66, 67). Another pharmacological treatment considered as AED is pyridoxine and PLP (pyridoxal phosphate) (70, 71). The patient reported by Jiao et al. is the second paper to report pyridoxine treatment in PIGT. Unfortunately, neither pyridoxine nor PLP were effective (38). The question concerning their effectiveness remains, however, open, due to still a limited number of *PIGT* cases.

##### PIGU (Phosphatidylinositol Glycan Anchor Biosynthesis Class U Protein; MIM 608528)

Patients identified with alterations of the *PIGU* gene presented with a high number of atypical symptoms, including strabismus (5/5), nystagmus (1/5), cerebellar atrophy (4/5), and visual impairment (3/5). Seizures, ID, and hypotonia were observed in every GPIBD21 case (MIM 618590; 20q11.22). Epileptic episodes were myoclonic in 5/5 patients, with additional grand mal seizures in one of them (77).

##### PIGV (Phosphatidylinositol Glycan Anchor Biosynthesis Class V Protein; MIM 610274)

A total of 9 patients diagnosed with GPIBD2 (MIM 239300; 1p36.11) were described, presenting a typical uniform clinical image of disorders of glycosylation. Dominant symptoms were seizures (8/9), followed by general development delay (7/9) and hypotonia (4/9). Most of the seizures had unknown onset (6/8), but two were classified as focal myoclonic episodes (2/8) (78).

##### PIGW (Phosphatidylinositol Glycan Anchor Biosynthesis Class W Protein; MIM 610275); PIGY (Phosphatidylinositol Glycan Anchor Biosynthesis Class Y Protein; MIM 610662)

A single GPIBD11 (MIM 616025; 17q12) patient was recently reported. He showed isolated symptomatology, consisting of ID and focal seizures (79).

Contrary to this case, four patients with identified *PIGY* gene alterations linked with GPIBD12 (MIM 616809; 4q22.1) presented with more varied symptoms. Visual impairment (2/4) and strabismus (1/4) were also observed, as well as from ID (4/4) and drug-resistant focal seizures (2/4) (80).

### Reporting Bias

As our systematic review was designed to include every report of GPIBD case, the reporting bias is not prominent at the stage of study selection. It could rather be attributed to the differences in interpretation of reports by the reviewers, leading to differences in the data extraction.

## Discussion

Around 150 different human proteins are anchored via GPI anchor, including > 40 enzymes (among others: alkaline phosphatase (ALP), 5′-nucleotidase, dipeptidase), adhesion molecules (f.e. contactins, glypicans, CD48), receptors (f.e. folate receptors, GDNF receptor alphas, FcγRIIIb), protease inhibitors (RECK), transcytotic transporters (GPIHBP1), and complement regulatory proteins (CD55 and CD59) (81).

The biosynthetic pathway of glycosylphosphatidylinositol involves three steps: biosynthesis of GPI anchor, attachment of protein and GPI anchor, and remodeling of glycosylphosphatidylinositol anchored proteins (GPI-APs) (3, 82). To sum it up, at least 16 reactions and more than 20 different proteins are encoded by phosphatidylinositol glycan (PIG) / post GPI attachment to proteins (PGAP) genes. At present, at least 31 genes involved in the biosynthesis of GPI synthesis and GPI-AP modifications have been reported. Pathogenic variants found in 22 genes are responsible for diseases' symptoms in human (3).

According to Murami et al., mutations in *PIGV, PIGO, PGAP2, PGAP3*, and *PIGW*, which affect late GPI-anchor synthesis, are associated with high levels of serum ALP, because GPI transamidase recognizes the currently incomplete GPI anchor and cleaves the GPI attachment signal, resulting in reduced GPI-AP surface levels and increased secretion into the extracellular space. The other GPIBDs may not be associated with elevated ALP (83).

Considering the involvement of GPI anchor in the variety of processes and structures in the human organism, it is important to highlight the devastating consequences of GPIBD, which are presented in this review. The main features of syndromes stemming from genetical disorders of GPI biosynthesis include global development delay, seizures, and hypotonia, with accompanying less frequent symptoms, such as cerebellar dysfunction and visual impairment. Interestingly, patients present some differences in symptomatology depending on location of genetic abnormalities. The phenotype of MCAHS (caused by *PIGA, PIGN*, and *PIGT*) is much more severe than the other GPIBDs. Although appearing at various times of onset, epileptic seizures in these patients are usually of focal and generalized motor semiology. Developmental delay concerns both intellectual and motor development, with similar mean onset (c.a. 6.8 months), and is mostly severe/profound. The identification of patients with GPIBDs has enabled the analysis of genotype–phenotype relationships for the molecular pathway due to overlapping spectrum of clinical presentation in majority of affected individuals.

One of the challenges for the future of handling patients with GPIBDs is the early and precise diagnosis. As the wide panel of genes and variety of their mutations may undermine the applicability of screening each newborn presenting with infantile onset neurological symptoms for the presence of GPIBDs, it is important to extract the features useful in differentiating patients with more probable diagnosis of GPIBD. Our systematic review of cases reported in the open-access English literature presents the concise summary of the phenotypes found in patients with different GPI biosynthesis defects. It may serve as the benchmark for clinical assessment of the GPIBD plausibility in patients, based on their clinical image.

Future directions of research should involve the differential diagnosis of GPIBD from other syndromes of similar phenotype and onset, which in their case could be Prader-Willi syndrome (PWS) and 1p36 monosomy syndrome (1p36MS)—a type of chromosome 1p36 deletion syndrome.

PWS is a rare (1:45,000), complex disorder, characterized by neonatal hypotonia, global developmental delay, short stature, and emotional problems. Although the epilepsy is not pathognomonic for this disorder, its prevalence ranges from 4 to 26% patients. In a study analyzing the seizures in PWS patients, the mean time of seizure onset was 4.5 years. Most of the patients (84.2%) in the follow-up achieved seizure freedom, reflected by the normalization of EEG image. Patients with lesions and abnormalities in CNS presented a higher risk of developing intractable epilepsy (84). In comparison to the results of our analysis, PWS patients are characterized by distinct and less severe phenotype of neurological disorders than GPIBD patients. The seizures in our group had earlier onset (mean: 1.5 vs. 4.5 years in PWS group) and were one of the leading symptoms (81% of patients). Most of the patients had severe or profound intellectual developmental delay, combined with DMD ranging from inability to roll-over to inability to walk. Dysmorphic facial features are present in both disorders; therefore, a short observational period could be key to assessing the differences in initial developmental delay and seizure onset, allowing to adequately assess the genetic screening.

Phenotype of 1p36MS is less distinct from GPIBDs—they both present facial dysmorphism, developmental delay, visual impairment, and high prevalence of seizures of similar mean time of onset (1.6 years). However, it has been recently suggested that the leading type of seizures in these disorders is infantile spasm (36.4% of examined patients), which is not very common in our group of patients with GPIBD. This observation prompts the conclusion that the children presenting with dysmorphic features, neurodevelopmental delay, and early onset of epileptic seizures should be screened for both 1p36MS and GPIBDs, with infantile spams type of seizures being an important factor indicating the more probable diagnosis of 1p36MS (85). Our systematic review may serve as a basis for the comparison with both of these syndromes.

A crucial issue in increasing efficacy of GPIBDs treatment is also, naturally, a better understanding of pathophysiology of these pathologies, which is yet another important issue to be addressed by future studies. In 2006 a model of human-induced pluripotent stem cells (hiPSC) lines with PIGA gene mutations were generated, using zinc finger nuclease technology. It provided the researchers with a reasonable model for studying the exact correlations between genetic alterations of genes involved in GPI biosynthesis and the changes occurring in the modified cells (86). These cells can also potentially be used for rescuing tissues of CNS, although with some limitations—not every alteration can be reversed with this method. Cultivation of iPSC also bears a risk of variability between the clones, whereas creating the isogenic cultures may result in unwanted mutations. As with many other medications, animals have a significant role in research on therapeutic strategies for GPIBD treatment. They may serve as a model for preclinical studies assessing the efficacy and safety of potential epigenetic or genetic modulators (87).

The studies reported in this systematic review have some limitations, of which the most prominent is the scarcity of data concerning the precise information on the symptoms in some of the reports. Due to the character of this review, the patients create a non-uniform population of children with GPIBD, which has further influence on the applicability of the results. Nonetheless, as the aim of the review was the inclusion of every available GPIBD case, such non-uniformity could not have been omitted.

The limitation of the review process, regardless of the influence of missing data on the data synthesis, is the quantity of the reviewers from different centers, which might have resulted in differences in interpretation of GPIBD reports. The risk of bias is usually assessed for the RCT studies, whereas observational studies are difficult to assess in this matter—the choice and the authors' modification of the tool for risk of bias assessment could be a matter of discussion.

## Data Availability Statement

The raw data supporting the conclusions of this article will be made available by the authors, without undue reservation.

## Author Contributions

JP contributed to conception and design of the study. MH and JH organized the database and performed the statistical analysis. JP, MH, JH, AT, MK, KS, AS, AJ-S, and RS wrote the first draft of the manuscript. All authors contributed to manuscript revision, read, and approved the submitted version.

## Conflict of Interest

The authors declare that the research was conducted in the absence of any commercial or financial relationships that could be construed as a potential conflict of interest.

## Publisher's Note

All claims expressed in this article are solely those of the authors and do not necessarily represent those of their affiliated organizations, or those of the publisher, the editors and the reviewers. Any product that may be evaluated in this article, or claim that may be made by its manufacturer, is not guaranteed or endorsed by the publisher.
