# Supplementary material for: Spectrum of Neurological Symptoms in Glycosylphosphatidylinositol Biosynthesis Defects: Systematic Review
Source: Front Neurol. 2022 Jan 4;12:758899. doi: 10.3389/fneur.2021.758899 (PMC8763846; doi:10.3389/fneur.2021.758899)
Supplement: Supplementary file 1 [file Table_1.DOCX]

**Table S1.** The summary of included studies

| Ref. | Author | Year | Gene | Patients | Gender | Nationality/Ethnicity | Mutation |
| --- | --- | --- | --- | --- | --- | --- | --- |
| 3 | Bayat et al. | 2020 | PIGA | 67 | 40M | - | Frameshift, deleterious mutations |
| 4 | Palmer et al. | 2016 | ARV1 | 1 | F | Lebanese | Deleterious homozygous in-frame mutation, truncating the zinc-binding motif |
| 5 | Darra et al. | 2021 | ARV1 | 2 | 2F | - | Compound heterozygous mutation |
| 6 | Alazami et al. | 2015 | ARV1 | 1 | - | - | - |
| 7 | Davids et al. | 2020 | ARV1 | 7 | 5F/2M | 2xMexican American; 5xLebanese Australian | Deleterious homozygous in-frame mutation |
| 8 | Yang et al. | 2013 | DPM1 | 1 | M | - | DPM1 point mutation |
| 9 | Kim et al. | 2000 | DPM1 | 2 | F/M | - | Point mutation and point mutation with 13-bp deletion |
| 10 | Imbach et al. | 2000 | DPM1 | 2 | F/M | - | Transversive and deletory mutations in DPM1 |
| 11 | Garcia-Silva et al. | 2004 | DPM1 | 1 | 9 | - | Homozygous DPM1 mutation |
| 12 | Dancourt et al. | 2006 | DPM1 | 2 | F/M | Algerian | Intronic mutation on DPM1 |
| 13 | Bursle et al. | 2017 | DPM1 | 1 | F | Caucasian | Point mutations |
| 14 | Schenk et al. | 2001 | MPDU1 | 3 | F/2M | - | Heterozygous and homozygous MPDU1 mutations |
| 15 | Kranz et al. | 2001 | MPDU1 | 1 | M | - | Homozygous pint mutation of MPDU1 |
| 16 | van Tol et al. | 2019 | MPDU1 | 2 | F/M | Iraqi | Homozygous missense mutation |
| 17 | Bastaki et al. | 2017 | MPDU1 | 3 | F/2M | 2xYemeni; 1xEmirati | Missense mutations in MPDU1 gene |
| 18 | Nguyen et al. | 2017 | GPAA1 | 10 | 5F/5M | 2xHispanic American; 1xWhite American; 2xEgyptian; 3xPakistani; 2xFinnish | Bi-allelic frameshift, intronic splicing, and missense mutations |
| 19 | Murakami et al. | 2014 | PGAP1 | 2 | F/M | Syrian | Nullizygous mutation |
| 20 | Williams et al. | 2015 | PGAP1 | 1 | M | - | Nullizygous mutation |
| 21 | Granzow et al. | 2015 | PGAP1 | 2 | F/M | Turkish | Homozygous loss-of-function mutations |
| 22 | Bosch et al. | 2015 | PGAP1 | 1 | M | - | Compound heterozygous mutations |
| 23 | Krawitz et al. | 2013 | PGAP2 | 2 | F/M | Finnish; Turkish | Compound heterozygous mutation; Homozygous mutation |
| 24 | Jezela-Stanek et al. | 2016 | PGAP2 | 1 | F | - | Heterozygous mutations |
|  |  |  | PIGN | 1 | F | - |  |
| 25 | Naseer et al. | 2016 | PGAP2 | 2 | F/M | - | Homozygous mutations |
| 26 | Perez et al. | 2017 | PGAP2 | 4 | 2F/2M | Beduin | dbSNP |
| 27 | Hansen et al. | 2013 | PGAP2 | 7 | 4F | - | Hypomorphic mutations |
| 28 | Howard et al. | 2014 | PGAP3 | 5 | 4F/M | 3xPakistani; 1xEuropean American; 1xSaudi-Arabian | Homozygous and compound heterozygous missense mutations |
| 29 | Knaus et al. | 2016 | PGAP3 | 8 | 6F/2M | 1xEuropean; 1xGerman; 2xFrench; 1xBritish; 2xPalestinian; 1xJapanese | Noncoding mutations |
| 30 | Nampoothiri et al. | 2017 | PGAP3 | 2 | F/M | Omani | Homozygous missense mutation |
| 31 | Abdel-Hamid et al. | 2017 | PGAP3 | 10 | 5F/5M | Egyptian | Homozygous frameshift mutations |
| 32 | Sakaguchi et al. | 2019 | PGAP3 | 1 | M | Croatian | Biallelic mutation |
| 33 | Da'as et al. | 2020 | PGAP3 | 1 | M | Qatari | Homozygous nonsense mutation |
| 34 | Bezuidenhout et al. | 2020 | PGAP3 | 3 | F/2M | Xhosa | Homozygous missense mutation |
| 35 | Tarailo-Graovac | 2015 | PIGA | 1 | M | Chinese | Germline nonsense mutation |
| 36 | Yang et al. | 2018 | PIGA | 1 | M | Chinese | Homozygous mutations |
| 37 | Neuhofer et al. | 2020 | PIGA | 1 | M | - | Hemizygous missense mutation |
| 38 | Jiao et al. | 2020 | PIGA | 8 | 8M | - | Missense hemizygous mutations |
|  |  |  | PIGN | 7 | 2F/5M | - | Missense, nonsense, synonymous, splicing and deleterious mutations |
|  |  |  | PIGT | 2 | 2M | Chinese | Missense mutations |
| 39 | Swoboda et al. | 2013 | PIGA | 3 | 3M | - | Germline mutations |
| 40 | Murakami et al. | 2019 | PIGB | 14 | - | - | Missense mutations |
| 41 | Makrythanasis et al. | 2016 | PIGG | 5 | 5F | 2xEgyptian; 1xJapanese; 2xPakistani | Compound heterozygous, homozygous and heterozygous deletrious mutations |
| 42 | Zhao et al. | 2017 | PIGG | 2 | F/M | Palestine | Homozygous nonsense mutation |
| 43 | Pagnamenta et al. | 2018 | PIGH | 2 | F/M | Pakistani | Homozygous mutations |
| 44 | Tremblay‐Laganière | 2021 | PIGH | 4 | F/3M | Indian; 2xGuatemalan; Azerbaijani | Homozygous missense mutation |
| 45 | Nguyen et al. | 2020 | PIGK | 12 | 5F/7M | 2xAsian Indian; 2xItalian; 1xFrench and Maghreb; 2xEgyptian; 1xNigerian, Igbo tribe; 3xPakistani | Homozygous missense mutation |
| 46 | Mogami et al. | 2017 | PIGL | 3 | F/2M | - | - |
| 47 | Ceroni et al. | 2018 | PIGL | 1 | M | Brazilian | Missense mutation |
| 48 | Altassan et al. | 2018 | PIGL | 1 | M | West African | Nonsense and missense mutation |
| 49 | Khayat et al. | 2015 | PIGN | 1 | F | Israeli-Arab | Homozygous deleterious mutation |
| 50 | Fleming et al. | 2015 | PIGN | 15 | 8F/7M | 2xCaucasian; African-American; Caucasian-African-American | Compound heteryzygous, missense and frameshift mutations |
| 51 | Thiffault et al. | 2017 | PIGN | 1 | M | - | Heterozygous mutations |
| 52 | Xiao et al. | 2020 | PIGN | 1 | - | Chinese | Compound hetorozygous variants |
| 53 | Sun et al. | 2021 | PIGN | 1 | M | Chinese | Homozygous splice mutation |
| 54 | Krawitz et al. | 2012 | PIGO | 3 | 3F | White British | Compound-heterozygous mutations |
| 55 | Nakamura et al. | 2014 | PIGO | 2 | F/M | - | Compound-heterozygous mutations |
| 56 | Tanigawa et al. | 2017 | PIGO | 6 | 4F/2M | - | Compound-heterozygous mutations |
| 57 | Krenn et al. | 2019 | PIGP | 1 | F | Polish | Homozygous frameshift mutations |
| 58 | Vetro et al. | 2020 | PIGP | 4 | 2F/2M | - | Homozygous deleterious frameshift mutations |
| 59 | Martin et al. | 2014 | PIGQ | 1 | M | West African | Homozygous mutations |
| 60 | Johnstone et al. | 2020 | PIGQ | 7 | 5F/2M | Turkish; European-Puerto Rican; 2xBritish Isles/French Canadian; Lebanese/Iraqi; Mexican; Afghani | Homozygous and compound heterozygous, missense and deleterious mutations |
| 61 | Nguyen et al. | 2018 | PIGS | 6 | 4M | - | Compound-heterozygous and homozygous deleterious and insertive mutations |
| 62 | Zhang et al. | 2020 | PIGS | 1 | M | Chinese | Transversive and duplicatory mutations |
| 63 | Efthymiou et al. | 2021 | PIGS | 6 | 2F/4M | - | Homozygous missense, nonsense and insertive mutations |
| 64 | Wu et al. | 2021 | PIGT | 1 | M | - | Compound heterozygous duplicatory mutation |
| 65 | Larsen et al. | 2019 | PIGT | 1 | M | Caucasian | Compound heterozygous mutations |
| 66 | Jezela-Stanek et al. | 2020 | PIGT | 7 | 3F/4M | - | Homozygous and compound heterozygous transversive mutations |
| 68 | Lam et al. | 2015 | PIGT | 2 | F/M | African-American/Caucasian | Compound heterozygous duplicatory frameshift mutation |
| 69 | Skaulie et al. | 2016 | PIGT | 2 | 2M | Somalian | Homozygous missense mutations |
| 70 | Nakashima et al. | 2014 | PIGT | 1 | F | - | Compound-heterozygous mutations |
| 71 | Kohashi et al. | 2018 | PIGT | 1 | M | - | Compound-heterozygous mutations |
| 72 | Pagnamenta et al. | 2017 | PIGT | 3 | F/2M | Caucasian; 2xAfghani | Compound-heterozygous and homozygous mutations |
| 73 | Yang et al. | 2018 | PIGT | 1 | M | Chinese | Homozygous missense mutation |
| 74 | Mason et al. | 2019 | PIGT | 1 | M | Greek | Compound-heterozygous mutations |
| 75 | Bayat et al. | 2021 | PIGT | 15 | 11F/4M | 1xCaucasian/African/Native American; 8xCaucasian; 1xAsian; 1xCaucasian/African; 3xRussian; Polish | Homozygous and compound heterozygous transversive mutations |
| 76 | Chandar et al. | 2021 | PIGT | 1 | F | Indian | Homozygous transversive mutation |
| 77 | Knaus et al. | 2019 | PIGU | 5 | 2F/3M | 1xTurkish;1xEuropean;1xNorwegian | Homozygous missense mutations |
| 78 | Horn et al. | 2013 | PIGV | 9 | 4F/5M | 7xGerman; 1xCroatian; 1xPakistani | Homozygous and heterozygous transversive mutations |
| 79 | Fu et al. | 2019 | PIGW | 1 | M | - | Compound-heterozygous transversive mutations |
| 80 | Ilkovski et al. | 2015 | PIGY | 4 | 3F/M | 2xCaucasian Australian; 2xPakistani | Homozygous missense mutations |

Footnotes: F, female; M, male
