# Supplementary material for: Spectrum of Neurological Symptoms in Glycosylphosphatidylinositol Biosynthesis Defects: Systematic Review
Source: Front Neurol. 2022 Jan 4;12:758899. doi: 10.3389/fneur.2021.758899 (PMC8763846; doi:10.3389/fneur.2021.758899)
Supplement: Supplementary file 2 [file Table_2.docx]

| Article type | - Brodsky RA. Paroxysmal nocturnal hemoglobinuria. Blood. 2014 124(18): 2804-11. doi: 10.1182/blood-2014-02-522128 - Hill A, DeZern AE, Kinoshita T, Brodsky RA. Paroxysmal nocturnal haemoglobinuria. Nat Rev Dis Primers. 2017 3: 17028. doi: 10.1038/nrdp.2017.28 - Paprocka J, Jezela-Stanek A, Tylki-Szymańska A, Grunewald S. Congenital Disorders of Glycosylation from a Neurological Perspective. Brain Sci. 2021 11(1): 88. doi: 10.3390/brainsci11010088 - Giblin SP, Midwood KS. Tenascin-C: Form versus function. Cell Adh Migr. 2015 9(1-2): 48-82. doi: 10.4161/19336918.2014.987587 - Stein MM, Thompson EE, Schoettler N, Helling BA, Magnaye KM, Stanhope C et al. A decade of research on the 17q12-21 asthma locus: Piecing together the puzzle. J Allergy Clin Immunol. 2018 142(3): 749-764.e3. doi: 10.1016/j.jaci.2017.12.974 - Hassel B, Rogne AG, Hope S. Intellectual Disability Associated With Pyridoxine-Responsive Epilepsies: The Need to Protect Cognitive Development. Front Psychiatry. 2019 10: 116. doi: 10.3389/fpsyt.2019.00116 - Pu JJ, Brodsky RA. Paroxysmal nocturnal hemoglobinuria from bench to bedside. Clin Transl Sci. 2011 4(3): 219-24. doi: 10.1111/j.1752-8062.2011.00262.x - Morren MA, Jaeken J, Visser G, Salles I, Van Geet C; NIHR BioResource et al. PIGO deficiency: palmoplantar keratoderma and novel mutations. Orphanet J Rare Dis. 2017 12(1):101. doi: 10.1186/s13023-017-0654-9 - Kettwig M, Elpeleg O, Wegener E, Dreha-Kulaczewski S, Henneke M, Gärtner J et al. Compound heterozygous variants in PGAP1 causing severe psychomotor retardation, brain atrophy, recurrent apneas and delayed myelination: a case report and literature review. BMC Neurol. 2016 16: 74. doi: 10.1186/s12883-016-0602-7 - Lee-Sundlov MM, Stowell SR, Hoffmeister KM. Multifaceted role of glycosylation in transfusion medicine, platelets, and red blood cells. J Thromb Haemost. 2020 18(7): 1535-1547. doi: 10.1111/jth.14874 - Almeida A, Layton M, Karadimitris A. Inherited glycosylphosphatidyl inositol deficiency: a treatable CDG. Biochim Biophys Acta. 2009 1792(9): 874-80. doi: 10.1016/j.bbadis.2008.12.010 - Johnstone DL, Nguyen TTM, Zambonin J, Kernohan KD, St-Denis A, Baratang NV et al. Early infantile epileptic encephalopathy due to biallelic pathogenic variants in PIGQ: Report of seven new subjects and review of the literature. J Inherit Metab Dis. 2020 43(6): 1321-1332. doi: 10.1002/jimd.12278 - Brandtzaeg P. Mucosal immunity: induction, dissemination, and effector functions. Scand J Immunol. 2009 70(6): 505-15. doi: 10.1111/j.1365-3083.2009.02319.x - Kinoshita T, Ohishi K, Takeda J. GPI-anchor synthesis in mammalian cells: genes, their products, and a deficiency. J Biochem. 1997 122(2): 251-7. doi: 10.1093/oxfordjournals.jbchem.a021746 - Tomita M. Biochemical background of paroxysmal nocturnal hemoglobinuria. Biochim Biophys Acta. 1999 1455(2-3): 269-86. doi: 10.1016/s0925-4439(99)00068-x - Johansen FE, Braathen R, Brandtzaeg P. Role of J chain in secretory immunoglobulin formation. Scand J Immunol. 2000 52(3): 240-8. doi: 10.1046/j.1365-3083.2000.00790.x - Luzzatto L. Recent advances in the pathogenesis and treatment of paroxysmal nocturnal hemoglobinuria. F1000Res. 2016 5: F1000 Faculty Rev-209. doi: 10.12688/f1000research.7288.1 - Parker CJ. Molecular basis of paroxysmal nocturnal hemoglobinuria. Stem Cells. 1996 14(4): 396-411. doi: 10.1002/stem.140396 - Abi Farraj L, Khatoun WD, Abou Chebel N, Wakim V, Dawali K, Ghassibe-Sabbagh M. Clinical, genetic, and molecular characterization of hyperphosphatasia with mental retardation: a case report and literature review. Diagn Pathol. 2019 14(1): 123. doi: 10.1186/s13000-019-0902-5 - Floris A, Piga M, Mangoni AA, Bortoluzzi A, Erre GL, Cauli A. Protective Effects of Hydroxychloroquine against Accelerated Atherosclerosis in Systemic Lupus Erythematosus. Mediators Inflamm. 2018 2018: 3424136. doi: 10.1155/2018/3424136 - Brodsky RA. Advances in the diagnosis and therapy of paroxysmal nocturnal hemoglobinuria. Blood Rev. 2008 22(2): 65-74. doi: 10.1016/j.blre.2007.10.002 - Veerreddy P. Hemoglobinuria misidentified as hematuria: review of discolored urine and paroxysmal nocturnal hemoglobinuria. Clin Med Insights Blood Disord. 2013 6: 7-17. doi: 10.4137/CMBD.S11517 - Nishimura J, Murakami Y, Kinoshita T. Paroxysmal nocturnal hemoglobinuria: An acquired genetic disease. Am J Hematol. 1999 62(3): 175-82. doi: 10.1002/(sici)1096-8652(199911)62:3<175::aid-ajh7>3.0.co;2-8 - Rosse WF. Paroxysmal nocturnal hemoglobinuria as a molecular disease. Medicine (Baltimore). 1997 76(2): 63-93. doi: 10.1097/00005792-199703000-00001 - Richards SJ, Hill A, Hillmen P. Recent advances in the diagnosis, monitoring, and management of patients with paroxysmal nocturnal hemoglobinuria. Cytometry B Clin Cytom. 2007 72(5): 291-8. doi: 10.1002/cyto.b.20358 - Nakakuma H. Mechanism of intravascular hemolysis in paroxysmal nocturnal hemoglobinuria (PNH). Am J Hematol. 1996 53(1): 22-9. doi: 10.1002/(SICI)1096-8652(199609)53:1<22::AID-AJH5>3.0.CO;2-7 - Jarva H, Meri S. Paroxysmal nocturnal haemoglobinuria: the disease and a hypothesis for a new treatment. Scand J Immunol. 1999 49(2): 119-25. doi: 10.1046/j.1365-3083.1999.00489.x - Al-Ani F, Chin-Yee I, Lazo-Langner A. Eculizumab in the management of paroxysmal nocturnal hemoglobinuria: patient selection and special considerations. Ther Clin Risk Manag. 2016 12: 1161-70. doi: 10.2147/TCRM.S96720 - Chen Y, Tao S, Deng Y, Song L, Yu L. Chronic myeloid leukemia transformation in a patient with paroxysmal nocturnal hemoglobinuria: a rare case report with literature review. Int J Clin Exp Med. 2015 8(5): 8226-9 - Yuasa T, Takahashi S, Hatake K, Yonese J, Fukui I. Biomarkers to predict response to sunitinib therapy and prognosis in metastatic renal cell cancer. Cancer Sci. 2011 102(11): 1949-57. doi: 10.1111/j.1349-7006.2011.02054.x - Spinelli FR, Barone F, Cacciapaglia F, Pecani A, Piga M. Atherosclerosis and Autoimmunity. Mediators Inflamm. 2018 2018: 6730421. doi: 10.1155/2018/6730421 - Piga A. Impact of bone disease and pain in thalassemia. Hematology Am Soc Hematol Educ Program. 2017 2017(1): 272-277. doi: 10.1182/asheducation-2017.1.272 - Aashiq M, Silverman DA, Na'ara S, Takahashi H, Amit M. Radioiodine-Refractory Thyroid Cancer: Molecular Basis of Redifferentiation Therapies, Management, and Novel Therapies. Cancers (Basel). 2019 11(9): 1382. doi: 10.3390/cancers11091382 - Ogawa S. Clonal hematopoiesis in acquired aplastic anemia. Blood. 2016 128(3): 337-47. doi: 10.1182/blood-2016-01-636381 - Voelkel NF, Vandivier RW, Tuder RM. Vascular endothelial growth factor in the lung. Am J Physiol Lung Cell Mol Physiol. 2006 290(2): L209-21. doi: 10.1152/ajplung.00185.2005 - Freeze HH. Understanding human glycosylation disorders: biochemistry leads the charge. J Biol Chem. 2013 288(10): 6936-45. doi: 10.1074/jbc.R112.429274 - Longo F, Piolatto A, Ferrero GB, Piga A. Ineffective Erythropoiesis in β-Thalassaemia: Key Steps and Therapeutic Options by Drugs. Int J Mol Sci. 2021 22(13): 7229. doi: 10.3390/ijms22137229 - Wiggs JL, Pasquale LR. Genetics of glaucoma. Hum Mol Genet. 2017 26(R1): R21-R27. doi: 10.1093/hmg/ddx184 - Merrill SA, Brodsky RA. Complement-driven anemia: more than just paroxysmal nocturnal hemoglobinuria. Hematology Am Soc Hematol Educ Program. 2018 2018(1): 371-376. doi: 10.1182/asheducation-2018.1.371 - Riaz SK, Iqbal Y, Malik MF. Diagnostic and therapeutic implications of the vascular endothelial growth factor family in cancer. Asian Pac J Cancer Prev. 16(5): 1677-82. doi: 10.7314/apjcp.2015.16.5.1677 - Mayrink J, Costa ML, Cecatti JG. Preeclampsia in 2018: Revisiting Concepts, Physiopathology, and Prediction. ScientificWorldJournal. 2018 2018: 6268276. doi: 10.1155/2018/6268276 - Gagliardi D, Mauri E, Magri F, Velardo D, Meneri M, Abati E et al. Can Intestinal Pseudo-Obstruction Drive Recurrent Stroke-Like Episodes in Late-Onset MELAS Syndrome? A Case Report and Review of the Literature. Front Neurol. 2019 10: 38. doi: 10.3389/fneur.2019.00038 - Mangoni AA, Zinellu A, Sotgia S, Carru C, Piga M, Erre GL. Protective Effects of Methotrexate against Proatherosclerotic Cytokines: A Review of the Evidence. Mediators Inflamm. 2017: 9632846. doi: 10.1155/2017/9632846 - Russo G, De Franceschi L, Colombatti R, Rigano P, Perrotta S, Voi V et al. Current challenges in the management of patients with sickle cell disease - A report of the Italian experience. Orphanet J Rare Dis. 2019 14(1): 120. doi: 10.1186/s13023-019-1099-0 - Smadja DM, Mentzer SJ, Fontenay M, Laffan MA, Ackermann M, Helms J, Jonigk D, Chocron R, Pier GB, Gendron N, Pons S, Diehl JL, Margadant C, Guerin C, Huijbers EJM, Philippe A, Chapuis N, Nowak-Sliwinska P, Karagiannidis C, Sanchez O, Kümpers P, Skurnik D, Randi AM, Griffioen AW. COVID-19 is a systemic vascular hemopathy: insight for mechanistic and clinical aspects. Angiogenesis. 2021 24(4): 755-788. doi: 10.1007/s10456-021-09805-6 - Hedera P. Hereditary Spastic Paraplegia Overview. 2000 Aug 15 [updated 2021 Feb 11]. In: Adam MP, Ardinger HH, Pagon RA, Wallace SE, Bean LJH, Mirzaa G, Amemiya A, editors. GeneReviews® [Internet]. Seattle (WA): University of Washington, Seattle; 1993–2021 - Longoni M, Pober BR, High FA. Congenital Diaphragmatic Hernia Overview. 2006 Feb 1 [updated 2020 Nov 5]. In: Adam MP, Ardinger HH, Pagon RA, Wallace SE, Bean LJH, Mirzaa G, Amemiya A, editors. GeneReviews® [Internet]. Seattle (WA): University of Washington, Seattle; 1993–2021 - Mayrink J, Costa ML, Cecatti JG. Preeclampsia in 2018: Revisiting Concepts, Physiopathology, and Prediction. ScientificWorldJournal. 2018 2018: 6268276. doi: 10.1155/2018/6268276 |
| --- | --- |
| Genetic Studies | - Jamieson E, Korologou-Linden R, Wootton RE, Guyatt AL, Battram T, Burrows K et al. Smoking, DNA Methylation, and Lung Function: a Mendelian Randomization Analysis to Investigate Causal Pathways. Am J Hum Genet. 2020 106(3):315-326. doi: 10.1016/j.ajhg.2020.01.015 - Albuquerque-Wendt A, Hütte HJ, Buettner FFR, Routier FH, Bakker H. Membrane Topological Model of Glycosyltransferases of the GT-C Superfamily. Int J Mol Sci. 2019 20(19): 4842. doi: 10.3390/ijms20194842 - Pronicka E, Piekutowska-Abramczuk D, Ciara E, Trubicka J, Rokicki D, Karkucińska-Więckowska A et al. New perspective in diagnostics of mitochondrial disorders: two years' experience with whole-exome sequencing at a national paediatric centre. J Transl Med. 2016 14(1): 174. doi: 10.1186/s12967-016-0930-9 - Wang Y, Hirata T, Maeda Y, Murakami Y, Fujita M, Kinoshita T. Free, unlinked glycosylphosphatidylinositols on mammalian cell surfaces revisited. J Biol Chem. 2019 294(13): 5038-5049. doi: 10.1074/jbc.RA119.007472 - Matabaro E, He Z, Liu YS, Zhang HJ, Gao XD, Fujita M. Molecular switching system using glycosylphosphatidylinositol to select cells highly expressing recombinant proteins. Sci Rep. 2017 7(1): 4033. doi: 10.1038/s41598-017-04330-3 - Long Q, Jeffries DE, Lin S, Chen X, He W, Wang Y et al. RedH and PigC Catalyze the Biosynthesis of Hybrubins via Phosphorylation of 4'-Methoxy-2,2'-Bipyrrole-5'-Carbaldehyde. Appl Environ Microbiol. 2020 86(2): e02331-19. doi: 10.1128/AEM.02331-19 - Lee GH, Fujita M, Nakanishi H, Miyata H, Ikawa M, Maeda Y et al. PGAP6, a GPI-specific phospholipase A2, has narrow substrate specificity against GPI-anchored proteins. J Biol Chem. 2020 295(42): 14501-14509. doi: 10.1074/jbc.RA120.014643 - Guo XY, Liu YS, Gao XD, Kinoshita T, Fujita M. Calnexin mediates the maturation of GPI-anchors through ER retention. J Biol Chem. 2020 295(48): 16393-16410. doi: 10.1074/jbc.RA120.015577 - Liu YS, Guo XY, Hirata T, Rong Y, Motooka D, Kitajima T et al. N-Glycan-dependent protein folding and endoplasmic reticulum retention regulate GPI-anchor processing. J Cell Biol. 2018 217(2): 585-599. doi: 10.1083/jcb.201706135 - Wang Y, Murakami Y, Yasui T, Wakana S, Kikutani H, Kinoshita T, Maeda Y. Significance of glycosylphosphatidylinositol-anchored protein enrichment in lipid rafts for the control of autoimmunity. J Biol Chem. 2013 288(35): 25490-25499. doi: 10.1074/jbc.M113.492611 - Bennett DC, Cazet A, Charest J, Contessa JN. MPDU1 regulates CEACAM1 and cell adhesion in vitro and in vivo. Glycoconj J. 2018 35(3): 265-274. doi: 10.1007/s10719-018-9819-6 - Nicklas JA, Carter EW, Albertini RJ. Both PIGA and PIGL mutations cause GPI-a deficient isolates in the Tk6 cell line. Environ Mol Mutagen. 2015 56(8): 663-73. doi: 10.1002/em.21953 - Tashima Y, Taguchi R, Murata C, Ashida H, Kinoshita T, Maeda Y. PGAP2 is essential for correct processing and stable expression of GPI-anchored proteins. Mol Biol Cell. 2006 17(3): 1410-20. doi: 10.1091/mbc.e05-11-1005 - Watanabe R, Murakami Y, Marmor MD, Inoue N, Maeda Y, Hino J et al. Initial enzyme for glycosylphosphatidylinositol biosynthesis requires PIG-P and is regulated by DPM2. EMBO J. 2000 19(16): 4402-11. doi: 10.1093/emboj/19.16.4402 - Couturier M, Bhalara HD, Chawrai SR, Monson R, Williamson NR, Salmond GPC et al. Substrate Flexibility of the Flavin-Dependent Dihydropyrrole Oxidases PigB and HapB Involved in Antibiotic Prodigiosin Biosynthesis. Chembiochem. 2020 21(4): 523-530. doi: 10.1002/cbic.201900424 - Maeda Y, Tanaka S, Hino J, Kangawa K, Kinoshita T. Human dolichol-phosphate-mannose synthase consists of three subunits, DPM1, DPM2 and DPM3. EMBO J. 2000 19(11): 2475-82. doi: 10.1093/emboj/19.11.2475 - Maeda Y, Tashima Y, Houjou T, Fujita M, Yoko-o T, Jigami Y et al. Fatty acid remodeling of GPI-anchored proteins is required for their raft association. Mol Biol Cell. 2007 18(4): 1497-506. doi: 10.1091/mbc.e06-10-0885 - Georgiev AG, Johansen J, Ramanathan VD, Sere YY, Beh CT, Menon AK. Arv1 regulates PM and ER membrane structure and homeostasis but is dispensable for intracellular sterol transport. Traffic. 2013 14(8): 912-21. doi: 10.1111/tra.12082 - Ashida H, Maeda Y, Kinoshita T. DPM1, the catalytic subunit of dolichol-phosphate mannose synthase, is tethered to and stabilized on the endoplasmic reticulum membrane by DPM3. J Biol Chem. 2006 281(2): 896-904. doi: 10.1074/jbc.M511311200 - Saudek V. Cystinosin, MPDU1, SWEETs and KDELR belong to a well-defined protein family with putative function of cargo receptors involved in vesicle trafficking. PLoS One. 7(2): e30876. doi: 10.1371/journal.pone.0030876 - Hiroi Y, Chen R, Sawa H, Hosoda T, Kudoh S, Kobayashi Y et al. Cloning of murine glycosyl phosphatidylinositol anchor attachment protein, GPAA1. Am J Physiol Cell Physiol. 2000 279(1): C205-12. doi: 10.1152/ajpcell.2000.279.1.C205 - Maeda Y, Tomita S, Watanabe R, Ohishi K, Kinoshita T. DPM2 regulates biosynthesis of dolichol phosphate-mannose in mammalian cells: correct subcellular localization and stabilization of DPM1, and binding of dolichol phosphate. EMBO J. 1998 17(17): 4920-9. doi: 10.1093/emboj/17.17.4920 - Lukacs M, Roberts T, Chatuverdi P, Stottmann RW. Glycosylphosphatidylinositol biosynthesis and remodeling are required for neural tube closure, heart development, and cranial neural crest cell survival. Elife. 2019 8: e45248. doi: 10.7554/eLife.45248 - Eisenhaber B, Eisenhaber S, Kwang TY, Grüber G, Eisenhaber F. Transamidase subunit GAA1/GPAA1 is a M28 family metallo-peptide-synthetase that catalyzes the peptide bond formation between the substrate protein's omega-site and the GPI lipid anchor's phosphoethanolamine. Cell Cycle. 13(12): 1912-7. doi: 10.4161/cc.28761 - Gandini R, Reichenbach T, Tan TC, Divne C. Structural basis for dolichylphosphate mannose biosynthesis. Nat Commun. 2017 8(1): 120. doi: 10.1038/s41467-017-00187-2 - Sundvold H, Sundvold-Gjerstad V, Malerød-Fjeld H, Haglund K, Stenmark H, Malerød L. Arv1 promotes cell division by recruiting IQGAP1 and myosin to the cleavage furrow. Cell Cycle. 15(5): 628-43. doi: 10.1080/15384101.2016.1146834 - Jaremko MJ, Lee DJ, Patel A, Winslow V, Opella SJ, McCammon JA et al. Manipulating Protein-Protein Interactions in Nonribosomal Peptide Synthetase Type II Peptidyl Carrier Proteins. Biochemistry. 2017 56(40): 5269-5273. doi: 10.1021/acs.biochem.7b00884 - Yi L, Bozkurt G, Li Q, Lo S, Menon AK, Wu H. Disulfide Bond Formation and N-Glycosylation Modulate Protein-Protein Interactions in GPI-Transamidase (GPIT). Sci Rep. 2017 8: 45912. doi: 10.1038/srep45912 - Hwang GW, Hayashi T, Kita K, Takahashi T, Kuge S, Naganuma A. siRNA-mediated inhibition of phosphatidylinositol glycan Class B (PIGB) confers resistance to methylmercury in HEK293 cells. J Toxicol Sci. 2007 32(5): 581-3. doi: 10.2131/jts.32.581 - Keller P, Payne JL, Tremml G, Greer PA, Gaboli M, Pandolfi PP et al. FES-Cre targets phosphatidylinositol glycan class A (PIGA) inactivation to hematopoietic stem cells in the bone marrow. J Exp Med. 2001 194(5): 581-9. doi: 10.1084/jem.194.5.581 - Lagor WR, Tong F, Jarrett KE, Lin W, Conlon DM, Smith M et al. Deletion of murine Arv1 results in a lean phenotype with increased energy expenditure. Nutr Diabetes. 2015 5(10): e181. doi: 10.1038/nutd.2015.32 - Liu SS, Jin F, Liu YS, Murakami Y, Sugita Y, Kato T et al. Functional Analysis of the GPI Transamidase Complex by Screening for Amino Acid Mutations in Each Subunit. Molecules. 2021 26(18): 5462. doi: 10.3390/molecules26185462 - Shechtman CF, Henneberry AL, Seimon TA, Tinkelenberg AH, Wilcox LJ, Lee E et al. Loss of subcellular lipid transport due to ARV1 deficiency disrupts organelle homeostasis and activates the unfolded protein response. J Biol Chem. 2011 286(14): 11951-9. doi: 10.1074/jbc.M110.215038 - Yang L, Gao Z, Hu L, Wu G, Yang X, Zhang L et al. Glycosylphosphatidylinositol Anchor Modification Machinery Deficiency Is Responsible for the Formation of Pro-Prion Protein (PrP) in BxPC-3 Protein and Increases Cancer Cell Motility. J Biol Chem. 2016 291(8): 3905-17. doi: 10.1074/jbc.M115.705830 - Sio YY, Anantharaman R, Lee SQE, Matta SA, Ng YT, Chew FT. The Asthma-associated PER1-like domain-containing protein 1 (PERLD1) Haplotype Influences Soluble Glycosylphosphatidylinositol Anchor Protein (sGPI-AP) Levels in Serum and Immune Cell Proliferation. Sci Rep. 2020 10(1): 715. doi: 10.1038/s41598-020-57592-9 - Bernat-Silvestre C, Sánchez-Simarro J, Ma Y, Montero-Pau J, Johnson K, Aniento F et al. AtPGAP1 functions as a GPI inositol-deacylase required for efficient transport of GPI-anchored proteins. Plant Physiol. 2021 kiab384. doi: 10.1093/plphys/kiab384 - Yuan X, Braunstein EM, Ye Z, Liu CF, Chen G, Zou J et al. Generation of glycosylphosphatidylinositol anchor protein-deficient blood cells from human induced pluripotent stem cells. Stem Cells Transl Med. 2013 2(11): 819-29. doi: 10.5966/sctm.2013-0069 - Lee GH, Fujita M, Takaoka K, Murakami Y, Fujihara Y, Kanzawa N et al. A GPI processing phospholipase A2, PGAP6, modulates Nodal signaling in embryos by shedding CRIPTO. J Cell Biol. 2016 215(5): 705-718. doi: 10.1083/jcb.201605121 - Su CT, Sinha S, Eisenhaber B, Eisenhaber F. Structural modelling of the lumenal domain of human GPAA1, the metallo-peptide synthetase subunit of the transamidase complex, reveals zinc-binding mode and two flaps surrounding the active site. Biol Direct. 2020 15(1): 14. doi: 10.1186/s13062-020-00266-3 - Wurmus R, Uyar B, Osberg B, Franke V, Gosdschan A, Wreczycka K. PiGx: reproducible genomics analysis pipelines with GNU Guix. Gigascience. 2018 7(12): giy123. doi: 10.1093/gigascience/giy123 - Kang JY, Hong Y, Ashida H, Shishioh N, Murakami Y, Morita YS et al. PIG-V involved in transferring the second mannose in glycosylphosphatidylinositol. J Biol Chem. 2005 280(10): 9489-97. doi: 10.1074/jbc.M413867200 - Pang V, Bates DO, Leach L. Regulation of human feto-placental endothelial barrier integrity by vascular endothelial growth factors: competitive interplay between VEGF-A165a, VEGF-A165b, PIGF and VE-cadherin. Clin Sci (Lond). 2017 131(23): 2763-2775. doi: 10.1042/CS20171252 - Lane WJ, Aeschlimann J, Vege S, Lomas-Francis C, Burgos A, Mah HH et al. PIGG defines the Emm blood group system. Sci Rep. 2021 11(1): 18545. doi: 10.1038/s41598-021-98090-w - Davis EM, Kim J, Menasche BL, Sheppard J, Liu X, Tan AC et al. Comparative Haploid Genetic Screens Reveal Divergent Pathways in the Biogenesis and Trafficking of Glycophosphatidylinositol-Anchored Proteins. Cell Rep. 2015 11(11): 1727-36. doi: 10.1016/j.celrep.2015.05.026 - Bemis JC, Avlasevich SL, Labash C, McKinzie P, Revollo J, Dobrovolsky VN et al. Glycosylphosphatidylinositol (GPI) anchored protein deficiency serves as a reliable reporter of Pig-a gene Mutation: Support from an in vitro assay based on L5178Y/Tk+/- cells and the CD90.2 antigen. Environ Mol Mutagen. 2018 59(1): 18-29. doi: 10.1002/em.22154 - Zoltewicz JS, Ashique AM, Choe Y, Lee G, Taylor S, Phamluong K et al. Wnt signaling is regulated by endoplasmic reticulum retention. PLoS One. 2009 4(7): e6191. doi: 10.1371/journal.pone.0006191 - Welti M, Hülsmeier AJ. Ethanol-induced impairment in the biosynthesis of N-linked glycosylation. J Cell Biochem. 2014 115(4): 754-62 - Ohishi K, Inoue N, Kinoshita T. PIG-S and PIG-T, essential for GPI anchor attachment to proteins, form a complex with GAA1 and GPI8. EMBO J. 2001 20(15): 4088-98. doi: 10.1093/emboj/20.15.4088 - Fu Y, Estoppey D, Roggo S, Pistorius D, Fuchs F, Studer C et al. Jawsamycin exhibits in vivo antifungal properties by inhibiting Spt14/Gpi3-mediated biosynthesis of glycosylphosphatidylinositol. Nat Commun. 2020 Jul 7;11(1):3387. doi: 10.1038/s41467-020-17221-5. Erratum in: Nat Commun. 2020 11(1): 3913 - Yuste-Checa P, Vega AI, Martín-Higueras C, Medrano C, Gámez A, Desviat LR et al. DPAGT1-CDG: Functional analysis of disease-causing pathogenic mutations and role of endoplasmic reticulum stress. PLoS One. 2017 12(6): e0179456. doi: 10.1371/journal.pone.0179456 - Da Pozzo E, Giacomelli C, Costa B, Cavallini C, Taliani S, Barresi E et al. TSPO PIGA Ligands Promote Neurosteroidogenesis and Human Astrocyte Well-Being. Int J Mol Sci. 2016 17(7): 1028. doi: 10.3390/ijms17071028 - Loeff FC, Falkenburg JHF, Hageman L, Huisman W, Veld SAJ, van Egmond HME et al. High Mutation Frequency of the PIGA Gene in T Cells Results in Reconstitution of GPI Anchor-/CD52- T Cells That Can Give Early Immune Protection after Alemtuzumab-Based T Cell-Depleted Allogeneic Stem Cell Transplantation. J Immunol. 2018 200(6): 2199-2208. doi: 10.4049/jimmunol.1701018 - Oswal N, Sahni NS, Bhattacharya A, Komath SS, Muthuswami R. Unique motifs identify PIG-A proteins from glycosyltransferases of the GT4 family. BMC Evol Biol. 2008 8: 168. doi: 10.1186/1471-2148-8-168 - Jerdeva GV, Tesar DB, Huey-Tubman KE, Ladinsky MS, Fraser SE, Bjorkman PJ. Comparison of FcRn- and pIgR-mediated transport in MDCK cells by fluorescence confocal microscopy. Traffic. 2010 11(9): 1205-20. doi: 10.1111/j.1600-0854.2010.01083.x - Eisenhaber B, Sinha S, Wong WC, Eisenhaber F. Function of a membrane-embedded domain evolutionarily multiplied in the GPI lipid anchor pathway proteins PIG-B, PIG-M, PIG-U, PIG-W, PIG-V, and PIG-Z. Cell Cycle. 17(7): 874-880. doi: 10.1080/15384101.2018 - Phonethepswath S, Bryce SM, Bemis JC, Dertinger SD. Erythrocyte-based Pig-a gene mutation assay: demonstration of cross-species potential. Mutat Res. 2008 657(2): 122-6. doi: 10.1016/j.mrgentox.2008.08.011 - Yan B, Huang M, Zeng C, Yao N, Zhang J, Yan B et al. Locally Produced IGF-1 Promotes Hypertrophy of the Ligamentum Flavum via the mTORC1 Signaling Pathway. Cell Physiol Biochem. 2018 48(1): 293-303. doi: 10.1159/000491729 - Austenaa L, Barozzi I, Chronowska A, Termanini A, Ostuni R, Prosperini E et al. The histone methyltransferase Wbp7 controls macrophage function through GPI glycolipid anchor synthesis. Immunity. 2012 36(4): 572-85. doi: 10.1016/j.immuni.2012.02.016 - Fujita M, Umemura M, Yoko-o T, Jigami Y. PER1 is required for GPI-phospholipase A2 activity and involved in lipid remodeling of GPI-anchored proteins. Mol Biol Cell. 2006 17(12): 5253-64. doi: 10.1091/mbc.e06-08-0715 - Ruggles KV, Garbarino J, Liu Y, Moon J, Schneider K, Henneberry A et al. A functional, genome-wide evaluation of liposensitive yeast identifies the "ARE2 required for viability" (ARV1) gene product as a major component of eukaryotic fatty acid resistance. J Biol Chem. 2014 289(7): 4417-31. doi: 10.1074/jbc.M113.515197 - Enk J, Levi A, Weisblum Y, Yamin R, Charpak-Amikam Y, Wolf DG et al. HSV1 MicroRNA Modulation of GPI Anchoring and Downstream Immune Evasion. Cell Rep. 2016 17(4): 949-956. doi: 10.1016/j.celrep.2016.09.077 - Ohishi K, Inoue N, Maeda Y, Takeda J, Riezman H, Kinoshita T. Gaa1p and gpi8p are components of a glycosylphosphatidylinositol (GPI) transamidase that mediates attachment of GPI to proteins. Mol Biol Cell. 2000 11(5): 1523-33. doi: 10.1091/mbc.11.5.1523 - Castillon GA, Michon L, Watanabe R. Apical sorting of lysoGPI-anchored proteins occurs independent of association with detergent-resistant membranes but dependent on their N-glycosylation. Mol Biol Cell. 2013 24(12): 2021-33. doi: 10.1091/mbc.E13-03-0160 - Li S, Yang J. System analysis of synonymous codon usage biases in archaeal virus genomes. J Theor Biol. 2014 355: 128-39. doi: 10.1016/j.jtbi.2014.03.022 - Yuan X, Braunstein EM, Ye Z, Liu CF, Chen G, Zou J et al. Generation of glycosylphosphatidylinositol anchor protein-deficient blood cells from human induced pluripotent stem cells. Stem Cells Transl Med. 2013 2(11): 819-29. doi: 10.5966/sctm.2013-0069 - Nakamura J, Gul H, Tian X, Bultman SJ, Swenberg JA. Detection of PIGO-deficient cells using proaerolysin: a valuable tool to investigate mechanisms of mutagenesis in the DT40 cell system. PLoS One. 7(3): e33563. doi: 10.1371/journal.pone.0033563 - Weigand JE, Boeckel JN, Gellert P, Dimmeler S. Hypoxia-induced alternative splicing in endothelial cells. PLoS One. 2012 7(8): e42697. doi: 10.1371/journal.pone.0042697 - Sawano A, Takahashi T, Yamaguchi S, Aonuma M, Shibuya M. Flt-1 but not KDR/Flk-1 tyrosine kinase is a receptor for placenta growth factor, which is related to vascular endothelial growth factor. Cell Growth Differ. 1996 7(2): 213-21 - Fujita M, Watanabe R, Jaensch N, Romanova-Michaelides M, Satoh T, Kato M et al. Sorting of GPI-anchored proteins into ER exit sites by p24 proteins is dependent on remodeled GPI. J Cell Biol. 2011 194(1): 61-75. doi: 10.1083/jcb.201012074 - Gao XH, Kondoh G, Tarutani M, Hara M, Inoue S, Nakanishi T et al. Rapid compensation for glycosylphosphatidylinositol anchor deficient keratinocytes after birth: visualization of glycosylphosphatidylinositol-anchored proteins in situ. J Invest Dermatol. 2002 118(6): 998-1002. doi: 10.1046/j.1523-1747.2002.01778.x - Shishioh N, Hong Y, Ohishi K, Ashida H, Maeda Y, Kinoshita T. GPI7 is the second partner of PIG-F and involved in modification of glycosylphosphatidylinositol. J Biol Chem. 2005 280(10): 9728-34. doi: 10.1074/jbc.M413755200 - Keysar SB, Fox MH. EMS mutant spectra generated by multi-parameter flow cytometry. Mutat Res. 2009 671(1-2): 6-12. doi: 10.1016/j.mrfmmm.2009.05.005 - Watanabe R, Kinoshita T, Masaki R, Yamamoto A, Takeda J, Inoue N. PIG-A and PIG-H, which participate in glycosylphosphatidylinositol anchor biosynthesis, form a protein complex in the endoplasmic reticulum. J Biol Chem. 1996 271(43): 26868-75. doi: 10.1074/jbc.271.43.26868 - Jasinski M, Keller P, Fujiwara Y, Orkin SH, Bessler M. GATA1-Cre mediates Piga gene inactivation in the erythroid/megakaryocytic lineage and leads to circulating red cells with a partial deficiency in glycosyl phosphatidylinositol-linked proteins (paroxysmal nocturnal hemoglobinuria type II cells). Blood. 2001 98(7): 2248-55. doi: 10.1182/blood.v98.7.2248 - Ramnarayanan SP, Cheng CA, Bastaki M, Tuma PL. Exogenous MAL reroutes selected hepatic apical proteins into the direct pathway in WIF-B cells. Mol Biol Cell. 2007 18(7): 2707-15. doi: 10.1091/mbc.e07-02-0096 - Barroso M, Sztul ES. Basolateral to apical transcytosis in polarized cells is indirect and involves BFA and trimeric G protein sensitive passage through the apical endosome. J Cell Biol. 1994 124(1-2): 83-100. doi: 10.1083/jcb.124.1.83 - Spurway TD, Dalley JA, High S, Bulleid NJ. Early events in glycosylphosphatidylinositol anchor addition. substrate proteins associate with the transamidase subunit gpi8p. J Biol Chem. 2001 276(19): 15975-82. doi: 10.1074/jbc.M010128200 - Hu Y, Li ZF, Wu X, Lu Q. Large induces functional glycans in an O-mannosylation dependent manner and targets GlcNAc terminals on alpha-dystroglycan. PLoS One. 2011 6(2): e16866. doi: 10.1371/journal.pone.0016866 - Clegg LE, Mac Gabhann F. A computational analysis of in vivo VEGFR activation by multiple co-expressed ligands. PLoS Comput Biol. 2017 13(3): e1005445. doi: 10.1371/journal.pcbi.1005445 - Zhan Y, Zhang G, Wang X, Qi Y, Bai S, Li D et al. Interplay between Cytoplasmic and Nuclear Androgen Receptor Splice Variants Mediates Castration Resistance. Mol Cancer Res. 2017 15(1): 59-68. doi: 10.1158/1541-7786.MCR-16-0236 - Jones MB, Tomiya N, Betenbaugh MJ, Krag SS. Analysis and metabolic engineering of lipid-linked oligosaccharides in glycosylation-deficient CHO cells. Biochem Biophys Res Commun. 2010 395(1): 36-41. doi: 10.1016/j.bbrc.2010.03.117 - Leung JC, Chan LY, Saleem MA, Mathieson PW, Tang SC, Lai KN. Combined blockade of angiotensin II and prorenin receptors ameliorates podocytic apoptosis induced by IgA-activated mesangial cells. Apoptosis. 2015 20(7): 907-20. doi: 10.1007/s10495-015-1117-1 - van den Dobbelsteen ME, van der Woude FJ, Schroeijers WE, van den Wall Bake AW, van Es LA, Daha MR. Binding of dimeric and polymeric IgA to rat renal mesangial cells enhances the release of interleukin 6. Kidney Int. 1994 46(2): 512-9. doi: 10.1038/ki.1994.302 - Vainauskas S, Menon AK. A conserved proline in the last transmembrane segment of Gaa1 is required for glycosylphosphatidylinositol (GPI) recognition by GPI transamidase. J Biol Chem. 2004 279(8): 6540-5. doi: 10.1074/jbc.M312191200 - Tian X, Chen Y, Nakamura J. Development of a novel PIG-A gene mutation assay based on a GPI-anchored fluorescent protein sensor. Genes Environ. 2019 41: 21. doi: 10.1186/s41021-019-0135-6 - Xu JG, Gong T, Wang YY, Zou T, Heng BC, Yang YQ et al. Inhibition of TGF-β Signaling in SHED Enhances Endothelial Differentiation. J Dent Res. 2018 97(2): 218-225. doi: 10.1177/0022034517733741 - Törmä A, Pitkänen JP, Huopaniemi L, Mattila P, Renkonen R. Concordant gene regulation related to perturbations of three GDP-mannose-related genes. FEMS Yeast Res. 2009 9(1): 63-72. doi: 10.1111/j.1567-1364.2008.00461.x - Hanaoka N, Kawaguchi T, Horikawa K, Nagakura S, Mitsuya H, Nakakuma H. Immunoselection by natural killer cells of PIGA mutant cells missing stress-inducible ULBP. Blood. 2006 107(3): 1184-91. doi: 10.1182/blood-2005-03-1337 - Ardiccioni C, Clarke OB, Tomasek D, Issa HA, von Alpen DC, Pond HL et al. Structure of the polyisoprenyl-phosphate glycosyltransferase GtrB and insights into the mechanism of catalysis. Nat Commun. 2016 7: 10175. doi: 10.1038/ncomms10175 - Hong Y, Maeda Y, Watanabe R, Inoue N, Ohishi K, Kinoshita T. Requirement of PIG-F and PIG-O for transferring phosphoethanolamine to the third mannose in glycosylphosphatidylinositol. J Biol Chem. 2000 275(27): 20911-9. doi: 10.1074/jbc.M001913200 - Maeda Y, Watanabe R, Harris CL, Hong Y, Ohishi K, Kinoshita K, Kinoshita T. PIG-M transfers the first mannose to glycosylphosphatidylinositol on the lumenal side of the ER. EMBO J. 2001 20(1-2): 250-61. doi: 10.1093/emboj/20.1.250 - Takahashi M, Inoue N, Ohishi K, Maeda Y, Nakamura N, Endo Y et al. PIG-B, a membrane protein of the endoplasmic reticulum with a large lumenal domain, is involved in transferring the third mannose of the GPI anchor. EMBO J. 1996 15(16): 4254-61 - Renegar KB, Jackson GD, Mestecky J. In vitro comparison of the biologic activities of monoclonal monomeric IgA, polymeric IgA, and secretory IgA. J Immunol. 1998 160(3): 1219-23 - Rees BJ, Tate M, Lynch AM, Thornton CA, Jenkins GJ, Walmsley RM et al. Development of an in vitro PIG-A gene mutation assay in human cells. Mutagenesis. 2017 32(2): 283-297. doi: 10.1093/mutage/gew059 - Kinoshita T. Enzymatic mechanism of GPI anchor attachment clarified. Cell Cycle. 13(12): 1838-9. doi: 10.4161/cc.29379 - Li D, Sun X, Yu F, Perle MA, Araten D, Boeke JD. Application of counter-selectable marker PIGA in engineering designer deletion cell lines and characterization of CRISPR deletion efficiency. Nucleic Acids Res. 2021 49(5): 2642-2654. doi: 10.1093/nar/gkab035 - Cindrova-Davies T, van Patot MT, Gardner L, Jauniaux E, Burton GJ, Charnock-Jones DS. Energy status and HIF signalling in chorionic villi show no evidence of hypoxic stress during human early placental development. Mol Hum Reprod. 2015 21(3): 296-308. doi: 10.1093/molehr/gau105 - Nakamura N, Inoue N, Watanabe R, Takahashi M, Takeda J, Stevens VL et al. Expression cloning of PIG-L, a candidate N-acetylglucosaminyl-phosphatidylinositol deacetylase. J Biol Chem. 1997 272(25): 15834-40. doi: 10.1074/jbc.272.25.15834 - Murakami Y, Siripanyaphinyo U, Hong Y, Tashima Y, Maeda Y, Kinoshita T. The initial enzyme for glycosylphosphatidylinositol biosynthesis requires PIG-Y, a seventh component. Mol Biol Cell. 2005 16(11): 5236-46. doi: 10.1091/mbc.e05-08-0743 - Murakami Y, Siripanyapinyo U, Hong Y, Kang JY, Ishihara S, Nakakuma H et al. PIG-W is critical for inositol acylation but not for flipping of glycosylphosphatidylinositol-anchor. Mol Biol Cell. 2003 14(10): 4285-95. doi: 10.1091/mbc.e03-03-0193 - Vainauskas S, Maeda Y, Kurniawan H, Kinoshita T, Menon AK. Structural requirements for the recruitment of Gaa1 into a functional glycosylphosphatidylinositol transamidase complex. J Biol Chem. 2002 277(34): 30535-42. doi: 10.1074/jbc.M205402200 - Karnan S, Konishi Y, Ota A, Takahashi M, Damdindorj L, Hosokawa Y et al. Simple monitoring of gene targeting efficiency in human somatic cell lines using the PIGA gene. PLoS One. 2012 7(10): e47389. doi: 10.1371/journal.pone.0047389 - Su T, Bryant DM, Luton F, Vergés M, Ulrich SM, Hansen KC et al. A kinase cascade leading to Rab11-FIP5 controls transcytosis of the polymeric immunoglobulin receptor. Nat Cell Biol. 2010 12(12): 1143-53. doi: 10.1038/ncb2118 - Emmerson CD, van der Vlist EJ, Braam MR, Vanlandschoot P, Merchiers P, de Haard HJ et al. Enhancement of polymeric immunoglobulin receptor transcytosis by biparatopic VHH. PLoS One. 2011 6(10): e26299. doi: 10.1371/journal.pone.0026299 - Park JE, Chen HH, Winer J, Houck KA, Ferrara N. Placenta growth factor. Potentiation of vascular endothelial growth factor bioactivity, in vitro and in vivo, and high affinity binding to Flt-1 but not to Flk-1/KDR. J Biol Chem. 1994 269(41): 25646-54 - Ahrens MJ, Li Y, Jiang H, Dudley AT. Convergent extension movements in growth plate chondrocytes require gpi-anchored cell surface proteins. Development. 2009 136(20): 3463-74. doi: 10.1242/dev.040592 - Bessler M, Rosti V, Peng Y, Cattoretti G, Notaro R, Ohsako S et al. Glycosylphosphatidylinositol-linked proteins are required for maintenance of a normal peripheral lymphoid compartment but not for lymphocyte development. Eur J Immunol. 2002 32(9): 2607-16. doi: 10.1002/1521-4141(200209)32:9<2607::AID-IMMU2607>3.0.CO;2-H - Pottekat A, Menon AK. Subcellular localization and targeting of N-acetylglucosaminyl phosphatidylinositol de-N-acetylase, the second enzyme in the glycosylphosphatidylinositol biosynthetic pathway. J Biol Chem. 2004 279(16): 15743-51. doi: 10.1074/jbc.M313537200 - Posadino AM, Cossu A, Piga A, Madrau MA, Del Caro A, Colombino M. Prune melanoidins protect against oxidative stress and endothelial cell death. Front Biosci (Elite Ed). 2011 3: 1034-41. doi: 10.2741/e309 - Kishore S, Khanna A, Zhang Z, Hui J, Balwierz PJ, Stefan M. The snoRNA MBII-52 (SNORD 115) is processed into smaller RNAs and regulates alternative splicing. Hum Mol Genet. 2010 19(7): 1153-64. doi: 10.1093/hmg/ddp585 - Müller G, Wied S, Piossek C, Bauer A, Bauer J, Frick W. Convergence and divergence of the signaling pathways for insulin and phosphoinositolglycans. Mol Med. 1998 4(5): 299-323 - Vemuganti SA, de Villena FP, O'Brien DA. Frequent and recent retrotransposition of orthologous genes plays a role in the evolution of sperm glycolytic enzymes. BMC Genomics. 2010 11: 285. doi: 10.1186/1471-2164-11-285 - Watanabe R, Inoue N, Westfall B, Taron CH, Orlean P, Takeda J et al. The first step of glycosylphosphatidylinositol biosynthesis is mediated by a complex of PIG-A, PIG-H, PIG-C and GPI1. EMBO J. 1998 17(4): 877-85. doi: 10.1093/emboj/17.4.877 - Kremer C, Breier G, Risau W, Plate KH. Up-regulation of flk-1/vascular endothelial growth factor receptor 2 by its ligand in a cerebral slice culture system. Cancer Res. 1997 57(17): 3852-9 - Ohishi K, Nagamune K, Maeda Y, Kinoshita T. Two subunits of glycosylphosphatidylinositol transamidase, GPI8 and PIG-T, form a functionally important intermolecular disulfide bridge. J Biol Chem. 2003 278(16): 13959-67. doi: 10.1074/jbc.M300586200 - Fosbrink M, Cudrici C, Tegla CA, Soloviova K, Ito T, Vlaicu S et al. Response gene to complement 32 is required for C5b-9 induced cell cycle activation in endothelial cells. Exp Mol Pathol. 2009 86(2): 87-94. doi: 10.1016/j.yexmp.2008.12.005 - Green CJ, Lichtlen P, Huynh NT, Yanovsky M, Laderoute KR, Schaffner W et al. Placenta growth factor gene expression is induced by hypoxia in fibroblasts: a central role for metal transcription factor-1. Cancer Res. 2001 61(6): 2696-703 - Graziani G, Ruffini F, Tentori L, Scimeca M, Dorio AS, Atzori MG et al. Antitumor activity of a novel anti-vascular endothelial growth factor receptor-1 monoclonal antibody that does not interfere with ligand binding. Oncotarget. 2016 7(45): 72868-72885. doi: 10.18632/oncotarget.12108 - Pike AF, Kramer NI, Blaauboer BJ, Seinen W, Brands R. A novel hypothesis for an alkaline phosphatase 'rescue' mechanism in the hepatic acute phase immune response. Biochim Biophys Acta. 2013 1832(12): 2044-56. doi: 10.1016/j.bbadis.2013.07.016 - Bastisch I, Tiede A, Deckert M, Ziolek A, Schmidt RE, Schubert J. Glycosylphosphatidylinositol (GPI)-deficient Jurkat T cells as a model to study functions of GPI-anchored proteins. Clin Exp Immunol. 2000 122(1): 49-54. doi: 10.1046/j.1365-2249.2000.01350.x - Saucan L, Palade GE. Membrane and secretory proteins are transported from the Golgi complex to the sinusoidal plasmalemma of hepatocytes by distinct vesicular carriers. J Cell Biol. 1994 125(4): 733-41. doi: 10.1083/jcb.125.4.733 - Wilson IB, Taylor JP, Webberley MC, Turner NJ, Flitsch SL. A novel mono-branched lipid phosphate acts as a substrate for dolichyl phosphate mannose synthetase. Biochem J. 1993 295 ( Pt 1)(Pt 1): 195-201. doi: 10.1042/bj2950195 - An P, Straka RJ, Pollin TI, Feitosa MF, Wojczynski MK, Daw EW et al. Genome-wide association studies identified novel loci for non-high-density lipoprotein cholesterol and its postprandial lipemic response. Hum Genet. 2014 133(7): 919-30. doi: 10.1007/s00439-014-1435-3 - Migdal M, Huppertz B, Tessler S, Comforti A, Shibuya M, Reich R et al. Neuropilin-1 is a placenta growth factor-2 receptor. J Biol Chem. 1998 273(35): 22272-8. doi: 10.1074/jbc.273.35.22272 - Takahama Y, Ohishi K, Tokoro Y, Sugawara T, Yoshimura Y, Okabe M et al. Functional competence of T cells in the absence of glycosylphosphatidylinositol-anchored proteins caused by T cell-specific disruption of the Pig-a gene. Eur J Immunol. 1998 28(7): 2159-66. doi: 10.1002/(SICI)1521-4141(199807)28:07<2159::AID-IMMU2159>3.0.CO;2-B - Braathen R, Sandvik A, Berntzen G, Hammerschmidt S, Fleckenstein B, Sandlie I et al. Identification of a polymeric Ig receptor binding phage-displayed peptide that exploits epithelial transcytosis without dimeric IgA competition. J Biol Chem. 2006 281(11): 7075-81. doi: 10.1074/jbc.M508509200 - Dertinger SD, Bryce SM, Phonethepswath S, Avlasevich SL. When pigs fly: immunomagnetic separation facilitates rapid determination of Pig-a mutant frequency by flow cytometric analysis. Mutat Res. 2011 721(2): 163-70. doi: 10.1016/j.mrgentox.2011.01.009 - Vainauskas S, Menon AK. Endoplasmic reticulum localization of Gaa1 and PIG-T, subunits of the glycosylphosphatidylinositol transamidase complex. J Biol Chem. 2005 280(16): 16402-9. doi: 10.1074/jbc.M414253200 - Inoue N, Kinoshita T, Orii T, Takeda J. Cloning of a human gene, PIG-F, a component of glycosylphosphatidylinositol anchor biosynthesis, by a novel expression cloning strategy. J Biol Chem. 1993 268(10): 6882-5 - Vergés M, Sebastián I, Mostov KE. Phosphoinositide 3-kinase regulates the role of retromer in transcytosis of the polymeric immunoglobulin receptor. Exp Cell Res. 2007 313(4): 707-18. doi: 10.1016/j.yexcr.2006.11.010 - Dunn DE, Yu J, Nagarajan S, Devetten M, Weichold FF, Medof ME et al. A knock-out model of paroxysmal nocturnal hemoglobinuria: Pig-a(-) hematopoiesis is reconstituted following intercellular transfer of GPI-anchored proteins. Proc Natl Acad Sci U S A. 1996 93(15): 7938-43. doi: 10.1073/pnas.93.15.7938 - Araten DJ, Krejci O, Ditata K, Wunderlich M, Sanders KJ, Zamechek L et al. The rate of spontaneous mutations in human myeloid cells. Mutat Res. 2013 749(1-2): 49-57. doi: 10.1016/j.mrfmmm.2013.05.004 - Pu M, Ma L, Ohkusu K, Isobe K, Taguchi R, Ikezawa H et al. Direct evidence of involvement of glycosylphosphatidylinositol-anchored proteins in the heavy metal-mediated signal delivery into T lymphocytes. FEBS Lett. 1995 361(2-3): 295-8. doi: 10.1016/0014-5793(95)00193-d - Tarallo V, Vesci L, Capasso O, Esposito MT, Riccioni T, Pastore L et al. A placental growth factor variant unable to recognize vascular endothelial growth factor (VEGF) receptor-1 inhibits VEGF-dependent tumor angiogenesis via heterodimerization. Cancer Res. 2010 70(5): 1804-13. doi: 10.1158/0008-5472.CAN-09-2609 - Vaerman JP, Langendries AE, Giffroy DA, Kaetzel CS, Fiani CM, Moro I et al. Antibody against the human J chain inhibits polymeric Ig receptor-mediated biliary and epithelial transport of human polymeric IgA. Eur J Immunol. 1998 28(1): 171-82. doi: 10.1002/(SICI)1521-4141(199801)28:01<171::AID-IMMU171>3.0.CO;2-# - Mohammed KA, Nasreen N, Tepper RS, Antony VB. Cyclic stretch induces PlGF expression in bronchial airway epithelial cells via nitric oxide release. Am J Physiol Lung Cell Mol Physiol. 2007 292(2): L559-66. doi: 10.1152/ajplung.00075.2006 - Luton F, Vergés M, Vaerman JP, Sudol M, Mostov KE. The SRC family protein tyrosine kinase p62yes controls polymeric IgA transcytosis in vivo. Mol Cell. 1999 4(4): 627-32. doi: 10.1016/s1097-2765(00)80213-0 - Johansen FE, Braathen R, Brandtzaeg P. The J chain is essential for polymeric Ig receptor-mediated epithelial transport of IgA. J Immunol. 2001 167(9): 5185-92. doi: 10.4049/jimmunol.167.9.5185 - Giffroy D, Courtoy PJ, Vaerman JP. Polymeric IgA binding to the human pIgR elicits intracellular signalling, but fails to stimulate pIgR-transcytosis. Scand J Immunol. 2001 53(1): 56-64. doi: 10.1046/j.1365-3083.2001.00843.x - Graupner A, Instanes C, Dertinger SD, Andersen JM, Lindeman B, Rongved TD et al. Single cell gel electrophoresis (SCGE) and Pig-a mutation assay in vivo-tools for genotoxicity testing from a regulatory perspective: a study of benzo[a]pyrene in Ogg1(-/-) mice. Mutat Res Genet Toxicol Environ Mutagen. 2014 772: 34-41. doi: 10.1016/j.mrgentox.2014.07.010 - Kessler A, Müller G, Wied S, Crecelius A, Eckel J. Signalling pathways of an insulin-mimetic phosphoinositolglycan-peptide in muscle and adipose tissue. Biochem J. 1998 330 ( Pt 1)(Pt 1): 277-86. doi: 10.1042/bj3300277 - Hong Y, Ohishi K, Watanabe R, Endo Y, Maeda Y, Kinoshita T. GPI1 stabilizes an enzyme essential in the first step of glycosylphosphatidylinositol biosynthesis. J Biol Chem. 1999 274(26): 18582-8. doi: 10.1074/jbc.274.26.18582 - Mascart-Lemone F, Duchateau J, Conley ME, Delacroix DL. A polymeric IgA response in serum can be produced by parenteral immunization. Immunology. 1987 61(4): 409-13 - Quintart J, Baudhuin P, Courtoy PJ. Marker enzymes in rat liver vesicles involved in transcellular transport. Eur J Biochem. 1989 184(3): 567-74. doi: 10.1111/j.1432-1033.1989.tb15051.x - Kontoghiorghes GJ, Piga A, Hoffbrand AV. Cytotoxic effects of the lipophilic iron chelator omadine. FEBS Lett. 1986 204(2): 208-12. doi: 10.1016/0014-5793(86)80813-4 - Itokawa T, Nokihara H, Nishioka Y, Sone S, Iwamoto Y, Yamada Y et al. Antiangiogenic effect by SU5416 is partly attributable to inhibition of Flt-1 receptor signaling. Mol Cancer Ther. 2002 1(5): 295-302 - Wheeler-Jones C, Abu-Ghazaleh R, Cospedal R, Houliston RA, Martin J, Zachary I. Vascular endothelial growth factor stimulates prostacyclin production and activation of cytosolic phospholipase A2 in endothelial cells via p42/p44 mitogen-activated protein kinase. FEBS Lett. 1997 420(1): 28-32. doi: 10.1016/s0014-5793(97)01481-6 - Sevlever D, Schiemann D, Guidubaldi J, Medof ME, Rosenberry TL. Accumulation of glucosaminyl(acyl)phosphatidylinositol in an S3 HeLa subline expressing normal dolicholphosphomannose synthase activity. Biochem J. 1997 321 ( Pt 3)(Pt 3): 837-44. doi: 10.1042/bj3210837 - Perez JH, Branch WJ, Smith L, Mullock BM, Luzio JP. Investigation of endosomal compartments involved in endocytosis and transcytosis of polymeric immunoglobulin A by subcellular fractionation of perfused isolated rat liver. Biochem J. 1988 251(3): 763-70. doi: 10.1042/bj2510763 - Scott LJ, Hubbard AL. Dynamics of four rat liver plasma membrane proteins and polymeric IgA receptor. Rates of synthesis and selective loss into the bile. J Biol Chem. 1992 Mar 267(9): 6099-106. Erratum in: J Biol Chem 1993 268(25): 19160 - Phonethepswath S, Franklin D, Torous DK, Bryce SM, Bemis JC, Raja S et al. Pig-a mutation: kinetics in rat erythrocytes following exposure to five prototypical mutagens. Toxicol Sci. 2010 114(1): 59-70. doi: 10.1093/toxsci/kfp289 - Rush JS, Subramanian T, Subramanian KL, Onono FO, Waechter CJ, Spielmann HP. Novel Citronellyl-Based Photoprobes Designed to Identify ER Proteins Interacting with Dolichyl Phosphate in Yeast and Mammalian Cells. Curr Chem Biol. 2015 9(2): 123-141. doi: 10.2174/2212796810666160216221610 - Mukhopadhyay S, Howlett AC. CB1 receptor-G protein association. Subtype selectivity is determined by distinct intracellular domains. Eur J Biochem. 2001 268(3): 499-505. doi: 10.1046/j.1432-1327.2001.01810.x - Kimoto T, Chikura S, Suzuki-Okada K, Kobayashi XM, Itano Y, Miura D et al. Effective use of the Pig-a gene mutation assay for mutagenicity screening: measuring CD59-deficient red blood cells in rats treated with genotoxic chemicals. J Toxicol Sci. 2012 37(5): 943-55. doi: 10.2131/jts.37.943 - Phaniraj S, Gao Z, Rane D, Peterson BR. Hydrophobic resorufamine derivatives: potent and selective red fluorescent probes of the endoplasmic reticulum of mammalian cells. Dyes Pigm. 2016 135: 127-133. doi: 10.1016/j.dyepig.2016.05.007 - Chepelev NL, Gagné R, Maynor T, Kuo B, Hobbs CA, Recio L, Yauk CL. Transcriptional profiling of male F344 rats suggests the involvement of calcium signaling in the mode of action of acrylamide-induced thyroid cancer. Food Chem Toxicol. 2017 107(Pt A): 186-200. doi: 10.1016/j.fct.2017.06.019 - Tarkowski A, Moldoveanu Z, Koopman WJ, Radl J, Haaijman JJ, Mestecky J. Cellular origins of human polymeric and monomeric IgA: enumeration of single cells secreting polymeric IgA1 and IgA2 in peripheral blood, bone marrow, spleen, gingiva and synovial tissue. Clin Exp Immunol. 1991 85(2): 341-8. doi: 10.1111/j.1365-2249.1991.tb05730.x - Mazurkewich S, Brott AS, Kimber MS, Seah SY. Structural and Kinetic Characterization of the 4-Carboxy-2-hydroxymuconate Hydratase from the Gallate and Protocatechuate 4,5-Cleavage Pathways of Pseudomonas putida KT2440. J Biol Chem. 2016 291(14): 7669-86. doi: 10.1074/jbc.M115.682054 - Lemaître-Coelho I, Altamirano GA, Barranco-Acosta C, Meykens R, Vaerman JP. In vivo experiments involving secretory component in the rat hepatic transfer of polymeric IgA from blood into bile. Immunology. 1981 43(2): 261-70 - Sancho J, González E, Rivera F, Escanero JF, Egido J. Hepatic and kidney uptake of soluble monomeric and polymeric IgA aggregates. Immunology. 1984 52(1): 161-7 - Oyama T, Ran S, Ishida T, Nadaf S, Kerr L, Carbone DP et al. Vascular endothelial growth factor affects dendritic cell maturation through the inhibition of nuclear factor-kappa B activation in hemopoietic progenitor cells. J Immunol. 1998 160(3): 1224-32 - Wieland WH, Orzáez D, Lammers A, Parmentier HK, Verstegen MW, Schots A. A functional polymeric immunoglobulin receptor in chicken (Gallus gallus) indicates ancient role of secretory IgA in mucosal immunity. Biochem J. 2004 380(Pt 3): 669-76. doi: 10.1042/BJ20040200 - Piberger AL, Krüger CT, Strauch BM, Schneider B, Hartwig A. BPDE-induced genotoxicity: relationship between DNA adducts, mutagenicity in the in vitro PIG-A assay, and the transcriptional response to DNA damage in TK6 cells. Arch Toxicol. 2018 92(1): 541-551. doi: 10.1007/s00204-017-2003-0 - Johnson K, Bertoli M, Phillips L, Töpf A, Van den Bergh P et al. Detection of variants in dystroglycanopathy-associated genes through the application of targeted whole-exome sequencing analysis to a large cohort of patients with unexplained limb-girdle muscle weakness. Skelet Muscle. 2018 8(1): 23. doi: 10.1186/s13395-018-0170-1 - Jones SM, Howell KE. Phosphatidylinositol 3-kinase is required for the formation of constitutive transport vesicles from the TGN. J Cell Biol. 1997 139(2): 339-49. doi: 10.1083/jcb.139.2.339 - Zheng Y, Gu Q, Xu X. Inhibition of ocular neovascularization by a novel peptide derived from human placenta growth factor-1. Acta Ophthalmol. 2012 90(7): e512-23. doi: 10.1111/j.1755-3768.2012.02476.x - Wallrabe H, Bonamy G, Periasamy A, Barroso M. Receptor complexes cotransported via polarized endocytic pathways form clusters with distinct organizations. Mol Biol Cell. 2007 18(6): 2226-43. doi: 10.1091/mbc.e06-08-0700 - Zhai K, Deng Y, Liang D, Tang J, Liu J, Yan B et al. RRM Transcription Factors Interact with NLRs and Regulate Broad-Spectrum Blast Resistance in Rice. Mol Cell. 2019 74(5): 996-1009.e7. doi: 10.1016/j.molcel.2019.03.013 - Dertinger SD, Phonethepswath S, Avlasevich SL, Torous DK, Mereness J, Bryce SM et al. Efficient monitoring of in vivo pig-a gene mutation and chromosomal damage: summary of 7 published studies and results from 11 new reference compounds. Toxicol Sci. 2012 130(2): 328-48. doi: 10.1093/toxsci/kfs258 - Stad RK, Bogers WM, Thoomes-van der Sluys ME, Van Es LA, Daha MR. In vivo activation of complement by IgA in a rat model. Clin Exp Immunol. 1992 87(1): 138-43. doi: 10.1111/j.1365-2249.1992.tb06427.x - Maurice C, Dertinger SD, Yauk CL, Marchetti F. Integrated In Vivo Genotoxicity Assessment of Procarbazine Hydrochloride Demonstrates Induction of Pig-a and LacZ Mutations, and Micronuclei, in MutaMouse Hematopoietic Cells. Environ Mol Mutagen. 2019 60(6): 505-512. doi: 10.1002/em.22271 - Prigent-Delecourt L, Coffin B, Colombel JF, Dehennin JP, Vaerman JP, Rambaud JC. Secretion of immunoglobulins and plasma proteins from the colonic mucosa: an in vivo study in man. Clin Exp Immunol. 1995 99(2): 221-5. doi: 10.1111/j.1365-2249.1995.tb05536.x - Ackermann LW, Wollenweber LA, Denning GM. IL-4 and IFN-gamma increase steady state levels of polymeric Ig receptor mRNA in human airway and intestinal epithelial cells. J Immunol. 1999 162(9): 5112-8 - Barratt J, Greer MR, Pawluczyk IZ, Allen AC, Bailey EM, Buck KS et al. Identification of a novel Fcalpha receptor expressed by human mesangial cells. Kidney Int. 2000 57(5): 1936-48. doi: 10.1046/j.1523-1755.2000.00043.x - Raikwar NS, Liu KZ, Thomas CP. Protein kinase C regulates FLT1 abundance and stimulates its cleavage in vascular endothelial cells with the release of a soluble PlGF/VEGF antagonist. Exp Cell Res. 2013 319(17): 2578-87. doi: 10.1016/j.yexcr.2013.07.005 - Kitamura T, Garofalo RP, Kamijo A, Hammond DK, Oka JA, Caflisch CR et al. Human intestinal epithelial cells express a novel receptor for IgA. J Immunol. 2000 164(10): 5029-34. doi: 10.4049/jimmunol.164.10.5029 - Stankowski LF Jr, Aardema MJ, Lawlor TE, Pant K, Roy S, Xu Y et al. Integration of Pig-a, micronucleus, chromosome aberration and comet assay endpoints in a 28-day rodent toxicity study with urethane. Mutagenesis. 2015 30(3): 335-42. doi: 10.1093/mutage/gev013 - Labash C, Carlson K, Avlasevich SL, Berg A, Bemis JC, MacGregor JT et al. Induction of Pig-a mutant erythrocytes in male and female rats exposed to 1,3-propane sultone, ethyl carbamate, or thiotepa. Mutat Res Genet Toxicol Environ Mutagen. 2015 782: 24-9. doi: 10.1016/j.mrgentox.2015.03.011 - Wickliffe JK, Dertinger SD, Torous DK, Avlasevich SL, Simon-Friedt BR, Wilson MJ. Diet-induced obesity increases the frequency of Pig-a mutant erythrocytes in male C57BL/6J mice. Environ Mol Mutagen. 2016 57(9): 668-677. doi: 10.1002/em.22058 - Ishii H, Inageta T, Mimori K, Saito T, Sasaki H, Isobe M, Mori M, Croce CM, Huebner K, Ozawa K, Furukawa Y. Frag1, a homolog of alternative replication factor C subunits, links replication stress surveillance with apoptosis. Proc Natl Acad Sci U S A. 2005 102(27): 9655-60. doi: 10.1073/pnas.0504222102 - Saredi S, Ardissone A, Ruggieri A, Mottarelli E, Farina L, Rinaldi R et al. Novel POMGNT1 point mutations and intragenic rearrangements associated with muscle-eye-brain disease. J Neurol Sci. 2012 318(1-2): 45-50. doi: 10.1016/j.jns.2012.04.008 - Zou J, Maeder ML, Mali P, Pruett-Miller SM, Thibodeau-Beganny S, Chou BK et al. Gene targeting of a disease-related gene in human induced pluripotent stem and embryonic stem cells. Cell Stem Cell. 2009 5(1): 97-110. doi: 10.1016/j.stem.2009.05.023 - Li N, Hwangbo C, Jaba IM, Zhang J, Papangeli I, Han J et al. miR-182 Modulates Myocardial Hypertrophic Response Induced by Angiogenesis in Heart. Sci Rep. 2016 6: 21228. doi: 10.1038/srep21228 - Basolo F, Fiore L, Fusco A, Giannini R, Albini A, Merlo GR et al. Potentiation of the malignant phenotype of the undifferentiated ARO thyroid cell line by insertion of the bcl-2 gene. Int J Cancer. 1999 81(6): 956-62. doi: 10.1002/(sici)1097-0215(19990611)81:6<956::aid-ijc19>3.0.co;2-n - Byrne M, Bennett RL, Cheng X, May WS. Progressive genomic instability in the Nup98-HoxD13 model of MDS correlates with loss of the PIG-A gene product. Neoplasia. 2014 16(8): 627-33. doi: 10.1016/j.neo.2014.07.004 - Poulsen NA, Robinson RC, Barile D, Larsen LB, Buitenhuis B. A genome-wide association study reveals specific transferases as candidate loci for bovine milk oligosaccharides synthesis. BMC Genomics. 2019 20(1): 404. doi: 10.1186/s12864-019-5786-y - Freitas-Andrade M, Carmeliet P, Charlebois C, Stanimirovic DB, Moreno MJ. PlGF knockout delays brain vessel growth and maturation upon systemic hypoxic challenge. J Cereb Blood Flow Metab. 2012 32(4): 663-75. doi: 10.1038/jcbfm.2011.167 - Jia X, Liu F, Zhao K, Lin J, Fang Y, Cai S et al. Identification of Essential Genes Associated With Prodigiosin Production in Serratia marcescens FZSF02. Front Microbiol. 2021 12: 705853. doi: 10.3389/fmicb.2021.705853 - Svejda B, Kidd M, Kazberouk A, Lawrence B, Pfragner R, Modlin IM. Limitations in small intestinal neuroendocrine tumor therapy by mTor kinase inhibition reflect growth factor-mediated PI3K feedback loop activation via ERK1/2 and AKT. Cancer. 2011 117(18): 4141-54. doi: 10.1002/cncr.26011 - Dertinger SD, Phonethepswath S, Franklin D, Weller P, Torous DK, Bryce SM et al. Integration of mutation and chromosomal damage endpoints into 28-day repeat dose toxicology studies. Toxicol Sci. 2010 115(2): 401-11. doi: 10.1093/toxsci/kfq070 - Bjerke K, Brandtzaeg P. Lack of relation between expression of HLA-DR and secretory component (SC) in follicle-associated epithelium of human Peyer's patches. Clin Exp Immunol. 1988 71(3): 502-7 - Cozzolino CA, Nilsson F, Iotti M, Sacchi B, Piga A, Farris S. Exploiting the nano-sized features of microfibrillated cellulose (MFC) for the development of controlled-release packaging. Colloids Surf B Biointerfaces. 2013 110: 208-16. doi: 10.1016/j.colsurfb.2013 - Ebersole JL, Novak MJ, Orraca L, Martinez-Gonzalez J, Kirakodu S, Chen KC et al. Hypoxia-inducible transcription factors, HIF1A and HIF2A, increase in aging mucosal tissues. Immunology. 2018 154(3): 452-464. doi: 10.1111/imm.12894 - Lorenzi MV, Horii Y, Yamanaka R, Sakaguchi K, Miki T. FRAG1, a gene that potently activates fibroblast growth factor receptor by C-terminal fusion through chromosomal rearrangement. Proc Natl Acad Sci U S A. 1996 93(17): 8956-61. doi: 10.1073/pnas.93.17.8956 - Damdindorj L, Karnan S, Ota A, Hossain E, Konishi Y, Hosokawa Y, Konishi H. A comparative analysis of constitutive promoters located in adeno-associated viral vectors. PLoS One. 2014 9(8): e106472. doi: 10.1371/journal.pone.0106472 - Ohmura T, Ledda-Columbano GM, Piga R, Columbano A, Glemba J, Katyal SL et al. Hepatocyte proliferation induced by a single dose of a peroxisome proliferator. Am J Pathol. 1996 148(3): 815-24 - Lee EO, Kim JG, Kim JD. Induction of vesicle-to-micelle transition by bile salts for DOPE vesicles incorporating immunoglobulin G. J Biochem. 1992 112(5): 671-6. doi: 10.1093/oxfordjournals.jbchem.a123957 - Karnan S, Ota A, Konishi Y, Wahiduzzaman M, Hosokawa Y, Konishi H. Improved methods of AAV-mediated gene targeting for human cell lines using ribosome-skipping 2A peptide. Nucleic Acids Res. 2016 44(6): e54. doi: 10.1093/nar/gkv1338 - Pflueger D, Terry S, Sboner A, Habegger L, Esgueva R, Lin PC et al. Discovery of non-ETS gene fusions in human prostate cancer using next-generation RNA sequencing. Genome Res. 2011 21(1): 56-67. doi: 10.1101/gr.110684.110 - Mammadzada P, Gudmundsson J, Kvanta A, André H. Differential hypoxic response of human choroidal and retinal endothelial cells proposes tissue heterogeneity of ocular angiogenesis. Acta Ophthalmol. 2016 94(8): 805-814. doi: 10.1111/aos.13119 - Puppin C, Puglisi F, Pellizzari L, Manfioletti G, Pestrin M, Pandolfi M et al. HEX expression and localization in normal mammary gland and breast carcinoma. BMC Cancer. 2006 : 192. doi: 10.1186/1471-2407-6-192 - Hirooka T, Yamamoto C, Yasutake A, Eto K, Kaji T. Expression of VEGF-related proteins in cultured human brain microvascular endothelial cells and pericytes after exposure to methylmercury. J Toxicol Sci. 2013 38(6): 837-45. doi: 10.2131/jts.38.837 - Song QY, Meng XR, Hinney A, Song JY, Huang T, Ma J, Wang HJ. Waist-hip ratio related genetic loci are associated with risk of impaired fasting glucose in Chinese children: a case control study. Nutr Metab (Lond). 2018 15: 34. doi: 10.1186/s12986-018-0270-2 - Chang CH, Li JR, Shu KH, Fu YC, Wu MJ. Hydronephrotic urine in the obstructed kidney promotes urothelial carcinoma cell proliferation, migration, invasion through the activation of mTORC2-AKT and ERK signaling pathways. PLoS One. 2013 Sep 4;8(9):e74300. doi: 10.1371/journal.pone.0074300. Erratum in: PLoS One. 2015 10(11): e0142702 - Lu Y, Zheng Y, Ai J, Xu X. Therapeutic effects of a novel PIGF-1 derived peptide, ZY-1, on corneal neovascularization in vitro and in vivo. Discov Med. 2016 21(117): 349-61 - Irvin J, Danchik C, Rall J, Babcock A, Pine M, Barnaby D et al. Bioactivity and composition of a preserved connective tissue matrix derived from human placental tissue. J Biomed Mater Res B Appl Biomater. 2018 106(8): 2731-2740. doi: 10.1002/jbm.b.34054 - Okada T, Kimura A, Kanki K, Nakatani S, Nagahara Y, Hiraga M et al. Liver Resident Macrophages (Kupffer Cells) Share Several Functional Antigens in Common with Endothelial Cells. Scand J Immunol. 2016 83(2): 139-50. doi: 10.1111/sji.12402 - Reiner AP, Lange EM, Jenny NS, Chaves PH, Ellis J, Li J et al. Soluble CD14: genomewide association analysis and relationship to cardiovascular risk and mortality in older adults. Arterioscler Thromb Vasc Biol. 2013 33(1): 158-64. doi: 10.1161/ATVBAHA.112.300421 - Mitsui T, Tani K, Maki J, Eguchi T, Tamada S, Eto E et al. Upregulation of Angiogenic Factors via Protein Kinase C and Hypoxia-induced Factor-1α Pathways under High-glucose Conditions in the Placenta. Acta Med Okayama. 2018 72(4): 359-367. doi: 10.18926/AMO/56171 - Sztul E, Colombo M, Stahl P, Samanta R. Control of protein traffic between distinct plasma membrane domains. Requirement for a novel 108,000 protein in the fusion of transcytotic vesicles with the apical plasma membrane. J Biol Chem. 1993 268(3): 1876-85 - Kernt M, Neubauer AS, Liegl RG, Hirneiss C, Alge CS, Wolf A et al. Sorafenib prevents human retinal pigment epithelium cells from light-induced overexpression of VEGF, PDGF and PlGF. Br J Ophthalmol. 2010 94(11): 1533-9. doi: 10.1136/bjo.2010.182162 - Howlett AC, Wilken GH, Pigg JJ, Houston DB, Lan R, Liu Q et al. Azido- and isothiocyanato-substituted aryl pyrazoles bind covalently to the CB1 cannabinoid receptor and impair signal transduction. J Neurochem. 2000 74(5): 2174-81. doi: 10.1046/j.1471-4159.2000.0742174.x - Dou J, Chen JS, Wang J, Chen GB, Zhao FS, Tang Q et al. Novel constructs of tuberculosis gene vaccine and its immune effect on mice. Cell Mol Immunol. 2005 2(1): 57-62 - Kim WY, Prudkin L, Feng L, Kim ES, Hennessy B, Lee JS et al. Epidermal growth factor receptor and K-Ras mutations and resistance of lung cancer to insulin-like growth factor 1 receptor tyrosine kinase inhibitors. Cancer. 2012 Aug 15;118(16):3993-4003. doi: 10.1002/cncr.26656. Epub 2012 Feb 22. Erratum in: Cancer. 2012 118(24): 6301 - Roetto A, Totaro A, Piperno A, Piga A, Longo F, Garozzo G et al. New mutations inactivating transferrin receptor 2 in hemochromatosis type 3. Blood. 2001 97(9): 2555-60. doi: 10.1182/blood.v97.9.2555 - Wang Q, Xu B, Du J, Xu X, Shang C, Wang X et al. MicroRNA-139-5p/Flt1/Wnt/β-catenin regulatory crosstalk modulates the progression of glioma. Int J Mol Med. 2018 41(4): 2139-2149. doi: 10.3892/ijmm.2018.3439 - Henno A, Blacher S, Lambert CA, Deroanne C, Noël A, Lapière C et al. Histological and transcriptional study of angiogenesis and lymphangiogenesis in uninvolved skin, acute pinpoint lesions and established psoriasis plaques: an approach of vascular development chronology in psoriasis. J Dermatol Sci. 2010 57(3): 162-9. doi: 10.1016/j.jdermsci.2009.12.006 - Kim JS, Kim ES, Liu D, Lee JJ, Solis L, Behrens C et al. Prognostic implications of tumoral expression of insulin like growth factors 1 and 2 in patients with non-small-cell lung cancer. Clin Lung Cancer. 2014 15(3): 213-21. doi: 10.1016/j.cllc.2013.12.006 - Harper SJ, Pringle JH, Wicks AC, Hattersley J, Layward L, Allen A et al. Expression of J chain mRNA in duodenal IgA plasma cells in IgA nephropathy. Kidney Int. 1994 45(3): 836-44. doi: 10.1038/ki.1994.110 - Venturoli C, Piga I, Curtarello M, Verza M, Esposito G, Venuto S et al. Genetic Perturbation of Pyruvate Dehydrogenase Kinase 1 Modulates Growth, Angiogenesis and Metabolic Pathways in Ovarian Cancer Xenografts. Cells. 2021 10(2): 325. doi: 10.3390/cells10020325 - Connelly L, Barham W, Pigg R, Saint-Jean L, Sherrill T, Cheng DS et al. Activation of nuclear factor kappa B in mammary epithelium promotes milk loss during mammary development and infection. J Cell Physiol. 2010 222(1): 73-81. doi: 10.1002/jcp.21922 - Alonso V, Escudero P, Fernández-Martos C, Salud A, Méndez M, Gallego J et al. Coexpression of p-IGF-1R and MMP-7 Modulates Panitumumab and Cetuximab Efficacy in RAS Wild-Type Metastatic Colorectal Cancer Patients. Neoplasia. 2018 20(7): 678-686. doi: 10.1016/j.neo.2018.05.004 - Origa R, Marceddu G, Danjou F, Perseu L, Satta S, Demartis FR et al. IFNL3 polymorphisms and HCV infection in patients with beta thalassemia. Ann Hepatol. 2015 14(3): 389-95 - Mortazavi Y, Tooze JA, Gordon-Smith EC, Rutherford TR. N-RAS gene mutation in patients with aplastic anemia and aplastic anemia/ paroxysmal nocturnal hemoglobinuria during evolution to clonal disease. Blood. 2000 95(2): 646-50 - Nakakido M, Tamura K, Chung S, Ueda K, Fujii R, Kiyotani K et al. Phosphatidylinositol glycan anchor biosynthesis, class X containing complex promotes cancer cell proliferation through suppression of EHD2 and ZIC1, putative tumor suppressors. Int J Oncol. 2016 49(3): 868-76. doi: 10.3892/ijo.2016.3607. Epub 2016 Jul 6 - Chang Y, He J, Tang J, Chen K, Wang Z, Xia Q et al. Investigation of the gene co-expression network and hub genes associated with acute mountain sickness. Hereditas. 2020 157(1): 13. doi: 10.1186/s41065-020-00127-z - Wegner J, Zillinger T, Schlee-Guimaraes TM, Bartok E, Schlee M. An epigenetic GPI anchor defect impairs TLR4 signaling in the B cell transdifferentiation model for primary human monocytes BLaER1. Sci Rep. 2021 Jul 22;11(1):14983. doi: 10.1038/s41598-021-94386-z. Erratum in: Sci Rep. 2021 11(1): 17661 - Lemieux CL, Douglas GR, Gingerich J, Phonethepswath S, Torous DK, Dertinger SD et al. Simultaneous measurement of benzo[a]pyrene-induced Pig-a and lacZ mutations, micronuclei and DNA adducts in Muta™ Mouse. Environ Mol Mutagen. 2011 52(9): 756-65. doi: 10.1002/em.20688 - van der Harst P, Zhang W, Mateo Leach I, Rendon A, Verweij N, Sehmi J et al. Seventy-five genetic loci influencing the human red blood cell. Nature. 2012 492(7429): 369-75. doi: 10.1038/nature11677 - Steri M, Orrù V, Idda ML, Pitzalis M, Pala M, Zara I et al. Overexpression of the Cytokine BAFF and Autoimmunity Risk. N Engl J Med. 2017 376(17): 1615-1626. doi: 10.1056/NEJMoa1610528 - Blanco I, Kuchenbaecker K, Cuadras D, Wang X, Barrowdale D, de Garibay GR et al. Assessing associations between the AURKA-HMMR-TPX2-TUBG1 functional module and breast cancer risk in BRCA1/2 mutation carriers. PLoS One. 2015 10(4): e0120020. doi: 10.1371/journal.pone.0120020 - Bian X, Biswas A, Huang X, Lee KJ, Li TK, Masuyama H et al. Short-Term Prediction of Adverse Outcomes Using the sFlt-1 (Soluble fms-Like Tyrosine Kinase 1)/PlGF (Placental Growth Factor) Ratio in Asian Women With Suspected Preeclampsia. Hypertension. 2019 74(1): 164-172. doi: 10.1161/HYPERTENSIONAHA.119.12760 - Andreetta C, Puppin C, Minisini A, Valent F, Pegolo E, Damante G et al. Thymidine phosphorylase expression and benefit from capecitabine in patients with advanced breast cancer. Ann Oncol. 2009 20(2): 265-71. doi: 10.1093/annonc/mdn592 - Park HJ, Kim SH, Jung YW, Shim SS, Kim JY, Cho YK et al. Screening models using multiple markers for early detection of late-onset preeclampsia in low-risk pregnancy. BMC Pregnancy Childbirth. 2014 14: 35. doi: 10.1186/1471-2393-14-35 - Zhu M, Ren Z, Possomato-Vieira JS, Khalil RA. Restoring placental growth factor-soluble fms-like tyrosine kinase-1 balance reverses vascular hyper-reactivity and hypertension in pregnancy. Am J Physiol Regul Integr Comp Physiol. 2016 311(3): R505-21. doi: 10.1152/ajpregu.00137.2016 - Lo WC, Dubey NK, Tsai FC, Lu JH, Peng BY, Chiang PC et al. Amelioration of Nicotine-Induced Osteoarthritis by Platelet-Derived Biomaterials Through Modulating IGF-1/AKT/IRS-1 Signaling Axis. Cell Transplant. 2020 Jan-Dec;29:963689720947348. doi: 10.1177/0963689720947348 - Song H, Zhang L, Luo Y, Zhang S, Li B. Effects of collagen peptides intake on skin ageing and platelet release in chronologically aged mice revealed by cytokine array analysis. J Cell Mol Med. 2018 22(1): 277-288. doi: 10.1111/jcmm.13317 - Parvatiyar K, Alsabbagh EM, Ochsner UA, Stegemeyer MA, Smulian AG, Hwang SH et al. Global analysis of cellular factors and responses involved in Pseudomonas aeruginosa resistance to arsenite. J Bacteriol. 2005 187(14): 4853-64. doi: 10.1128/JB.187.14.4853-4864.2005 - Kendler KS, Kalsi G, Holmans PA, Sanders AR, Aggen SH, Dick DM et al. Genomewide association analysis of symptoms of alcohol dependence in the molecular genetics of schizophrenia (MGS2) control sample. Alcohol Clin Exp Res. 2011 35(5): 963-75. doi: 10.1111/j.1530-0277.2010.01427.x - Koster MP, Wortelboer EJ, Stoutenbeek P, Visser GH, Schielen PC. Modeling Down syndrome screening performance using first-trimester serum markers. Ultrasound Obstet Gynecol. 2011 38(2): 134-9. doi: 10.1002/uog.8881 - Li C, Zhou Y, Loberg A, Tahara SM, Malik P, Kalra VK. Activated Transcription Factor 3 in Association with Histone Deacetylase 6 Negatively Regulates MicroRNA 199a2 Transcription by Chromatin Remodeling and Reduces Endothelin-1 Expression. Mol Cell Biol. 2016 36(22): 2838-2854. doi: 10.1128/MCB.00345-16 - Trump N, McTague A, Brittain H, Papandreou A, Meyer E, Ngoh A et al. Improving diagnosis and broadening the phenotypes in early-onset seizure and severe developmental delay disorders through gene panel analysis. J Med Genet. 2016 53(5): 310-7. doi: 10.1136/jmedgenet-2015-103263 - Nowak J, Wozniak J, Mendek-Czajkowska E, Dlugokecka A, Mika-Witkowska R, Rogatko-Koros M et al. Potential link between MHC-self-peptide presentation and hematopoiesis; the analysis of HLA-DR expression in CD34-positive cells and self-peptide presentation repertoires of MHC molecules associated with paroxysmal nocturnal hemoglobinuria. Cell Biochem Biophys. 2013 65(3): 321-33. doi: 10.1007/s12013-012-9435-1 - Zimna A, Wiernicki B, Kolanowski T, Rozwadowska N, Malcher A, Labedz W et al. Biological and Pro-Angiogenic Properties of Genetically Modified Human Primary Myoblasts Overexpressing Placental Growth Factor in In Vitro and In Vivo Studies. Arch Immunol Ther Exp (Warsz). 2018 66(2): 145-159. doi: 10.1007/s00005-017-0486-2 - Braga DL, Mota STS, Zóia MAP, Lima PMAP, Orsolin PC, Vecchi L et al. Ethanolic Extracts from Azadirachta indica Leaves Modulate Transcriptional Levels of Hormone Receptor Variant in Breast Cancer Cell Lines. Int J Mol Sci. 2018 19(7): 1879. doi: 10.3390/ijms19071879 - Bezzi G, Piga EJ, Binolfi A, Armas P. CNBP Binds and Unfolds In Vitro G-Quadruplexes Formed in the SARS-CoV-2 Positive and Negative Genome Strands. Int J Mol Sci. 2021 22(5): 2614. doi: 10.3390/ijms22052614 - Lowe N, Rees JS, Roote J, Ryder E, Armean IM, Johnson G et al. Analysis of the expression patterns, subcellular localisations and interaction partners of Drosophila proteins using a pigP protein trap library. Development. 2014 141(20): 3994-4005. doi: 10.1242/dev.111054 - Alpini G, Glaser SS, Zhang JP, Francis H, Han Y, Gong J et al. Regulation of placenta growth factor by microRNA-125b in hepatocellular cancer. J Hepatol. 2011 55(6): 1339-45. doi: 10.1016/j.jhep.2011.04.015 - Roland CL, May CD, Watson KL, Al Sannaa GA, Dineen SP, Feig R et al. Analysis of Clinical and Molecular Factors Impacting Oncologic Outcomes in Undifferentiated Pleomorphic Sarcoma. Ann Surg Oncol. 2016 23(7): 2220-8. doi: 10.1245/s10434-016-5115-5 - Cauli A, Shaw J, Giles J, Hatano H, Rysnik O, Payeli S et al. The arthritis-associated HLA-B*27:05 allele forms more cell surface B27 dimer and free heavy chain ligands for KIR3DL2 than HLA-B*27:09. Rheumatology (Oxford). 2013 52(11): 1952-62. doi: 10.1093/rheumatology/ket219 - Ma Q, Gu JT, Wang B, Feng J, Yang L, Kang XW et al. PlGF signaling and macrophage repolarization contribute to the anti-neoplastic effect of metformin. Eur J Pharmacol. 2019 863: 172696. doi: 10.1016/j.ejphar.2019.172696 - Zhang R, Gehlen J, Kawalia A, Melissari MT, Dakal TC, Menon AM et al. Human exome and mouse embryonic expression data implicate ZFHX3, TRPS1, and CHD7 in human esophageal atresia. PLoS One. 2020 15(6): e0234246. doi: 10.1371/journal.pone.0234246 - Wu Y, Zhang J, Peng B, Tian D, Zhang D, Li Y, Feng X, Liu J, Li J, Zhang T, Liu X, Lu J, Chen B, Wang S. Generating viable mice with heritable embryonically lethal mutations using the CRISPR-Cas9 system in two-cell embryos. Nat Commun. 2019 10(1): 2883. doi: 10.1038/s41467-019-10748-2 - Lyu Z, Jin H, Yan Z, Hu K, Jiang H, Peng H et al. Effects of NRP1 on angiogenesis and vascular maturity in endothelial cells are dependent on the expression of SEMA4D. Int J Mol Med. 2020 46(4): 1321-1334. doi: 10.3892/ijmm.2020.4692 - McLaughlin K, Nadeem L, Wat J, Baczyk D, Lye SJ, Kingdom JC. Low molecular weight heparin promotes transcription and release of placental growth factor from endothelial cells. Am J Physiol Heart Circ Physiol. 2020 318(4): H1008-H1017. doi: 10.1152/ajpheart.00109.2020 - Pramatirta AY, Mose J, Effendi JS, Krisnadi SR, Anwar AD, Fauziah PN et al. Correlation between cell-free mRNA expressions and PLGF protein level in severe preeclampsia. BMC Res Notes. 2015 8: 208. doi: 10.1186/s13104-015-1186-9 - Novitskaya T, Baserga M, de Caestecker MP. Organ-specific defects in insulin-like growth factor and insulin receptor signaling in late gestational asymmetric intrauterine growth restriction in Cited1 mutant mice. Endocrinology. 2011 152(6): 2503-16. doi: 10.1210/en.2010-1385 |
| Studies on non-human models | - Piłsyk S, Perlinska-Lenart U, Janik A, Gryz E, Ajchler-Adamska M, Kruszewska JS. Yil102c-A is a Functional Homologue of the DPMII Subunit of Dolichyl Phosphate Mannose Synthase in Saccharomyces cerevisiae. Int J Mol Sci. 2020 21(23): 8938. doi: 10.3390/ijms21238938 - Villasmil ML, Barbosa AD, Cunningham JL, Siniossoglou S, Nickels JT Jr. An Erg11 lanosterol 14-α-demethylase-Arv1 complex is required for Candida albicans virulence. PLoS One. 2020 15(7): e0235746. doi: 10.1371/journal.pone.0235746 - Budirahardja Y, Doan TD, Zaidel-Bar R. Glycosyl phosphatidylinositol anchor biosynthesis is essential for maintaining epithelial integrity during Caenorhabditis elegans embryogenesis. PLoS Genet. 2015 11(3): e1005082. doi: 10.1371/journal.pgen.1005082 - Fineran PC, Williamson NR, Lilley KS, Salmond GP. Virulence and prodigiosin antibiotic biosynthesis in Serratia are regulated pleiotropically by the GGDEF/EAL domain protein, PigX. J Bacteriol. 2007 189(21): 7653-62. doi: 10.1128/JB.00671-07 - Okai H, Ikema R, Nakamura H, Kato M, Araki M, Mizuno A et al. Cold-sensitive phenotypes of a yeast null mutant of ARV1 support its role as a GPI flippase. FEBS Lett. 2020 594(15): 2431-2439. doi: 10.1002/1873-3468.13843 - Oortveld MA, Keerthikumar S, Oti M, Nijhof B, Fernandes AC, Kochinke K et al. Human intellectual disability genes form conserved functional modules in Drosophila. PLoS Genet. 2013 9(10): e1003911. doi: 10.1371/journal.pgen.1003911 - Shanks RM, Lahr RM, Stella NA, Arena KE, Brothers KM, Kwak DH et al. A Serratia marcescens PigP homolog controls prodigiosin biosynthesis, swarming motility and hemolysis and is regulated by cAMP-CRP and HexS. PLoS One. 2013 8(3): e57634. doi: 10.1371/journal.pone.0057634 - Cortes LK, Scarcelli JJ, Taron CH. Complementation of essential yeast GPI mannosyltransferase mutations suggests a novel specificity for certain Trypanosoma and Plasmodium PigB proteins. PLoS One. 2014 9(1): e87673. doi: 10.1371/journal.pone.0087673 - Rodríguez de Los Santos M, Rivalan M, David FS, Stumpf A, Pitsch J, Tsortouktzidis D et al. A CRISPR-Cas9-engineered mouse model for GPI-anchor deficiency mirrors human phenotypes and exhibits hippocampal synaptic dysfunctions. Proc Natl Acad Sci U S A. 2021 118(2): e2014481118. doi: 10.1073/pnas.2014481118 - Gristwood T, McNeil MB, Clulow JS, Salmond GP, Fineran PC. PigS and PigP regulate prodigiosin biosynthesis in Serratia via differential control of divergent operons, which include predicted transporters of sulfur-containing molecules. J Bacteriol. 2011 193(5): 1076-85. doi: 10.1128/JB.00352-10 - Villasmil ML, Nickels JT Jr. Determination of the membrane topology of Arv1 and the requirement of the ER luminal region for Arv1 function in Saccharomyces cerevisiae. FEMS Yeast Res. 2011 11(6): 524-7. doi: 10.1111/j.1567-1364.2011.00737.x - Gallo-Ebert C, Francisco J, Liu HY, Draper R, Modi K, Hayward MD et al. Mice lacking ARV1 have reduced signs of metabolic syndrome and non-alcoholic fatty liver disease. J Biol Chem. 2018 293(16): 5956-5974. doi: 10.1074/jbc.RA117.000800 - Kalivoda EJ, Stella NA, Aston MA, Fender JE, Thompson PP, Kowalski RP et al. Cyclic AMP negatively regulates prodigiosin production by Serratia marcescens. Res Microbiol. 2010 161(2): 158-67. doi: 10.1016/j.resmic.2009.12.004 - Sartelet A, Li W, Pailhoux E, Richard C, Tamma N, Karim et al. Genome-wide next-generation DNA and RNA sequencing reveals a mutation that perturbs splicing of the phosphatidylinositol glycan anchor biosynthesis class H gene (PIGH) and causes arthrogryposis in Belgian Blue cattle. BMC Genomics. 2015 16(1): 316. doi: 10.1186/s12864-015-1528-y - McKean DM, Niswander L. Defects in GPI biosynthesis perturb Cripto signaling during forebrain development in two new mouse models of holoprosencephaly. Biol Open. 2012 1(9): 874-83. doi: 10.1242/bio.20121982 - Carmean V, Yonkers MA, Tellez MB, Willer JR, Willer GB, Gregg RG, Geisler R, Neuhauss SC, Ribera AB. pigk Mutation underlies macho behavior and affects Rohon-Beard cell excitability. J Neurophysiol. 2015 114(2): 1146-57. doi: 10.1152/jn.00355.2015 - Wu Y, Xiao N, Yu L, Pan C, Li Y, Zhang X et al. Combination Patterns of Major R Genes Determine the Level of Resistance to the M. oryzae in Rice (Oryza sativa L.). PLoS One. 2015 10(6): e0126130. doi: 10.1371/journal.pone.0126130 - Ueda Y, Yamaguchi R, Ikawa M, Okabe M, Morii E, Maeda Y et al. PGAP1 knock-out mice show otocephaly and male infertility. J Biol Chem. 2007 282(42): 30373-80. doi: 10.1074/jbc.M705601200 - Villasmil ML, Ansbach A, Nickels JT Jr. The putative lipid transporter, Arv1, is required for activating pheromone-induced MAP kinase signaling in Saccharomyces cerevisiae. Genetics. 2011 187(2): 455-65. doi: 10.1534/genetics.110.120725 - Kajiwara K, Watanabe R, Pichler H, Ihara K, Murakami S, Riezman H et al. Yeast ARV1 is required for efficient delivery of an early GPI intermediate to the first mannosyltransferase during GPI assembly and controls lipid flow from the endoplasmic reticulum. Mol Biol Cell. 2008 19(5): 2069-82. doi: 10.1091/mbc.e07-08-0740 - Gallo-Ebert C, McCourt PC, Donigan M, Villasmil ML, Chen W, Pandya D et al. Arv1 lipid transporter function is conserved between pathogenic and nonpathogenic fungi. Fungal Genet Biol. 2012 49(2): 101-13. doi: 10.1016/j.fgb.2011.11.006 - Kamariah N, Eisenhaber F, Adhikari S, Eisenhaber B, Grüber G. Purification and crystallization of yeast glycosylphosphatidylinositol transamidase subunit PIG-S (PIG-S(71-467)). Acta Crystallogr Sect F Struct Biol Cryst Commun. 2011 67(Pt 8): 896-9. doi: 10.1107/S1744309111024080 - Yamamoto-Hino M, Kawaguchi K, Ono M, Furukawa K, Goto S. Lamin is essential for nuclear localization of the GPI synthesis enzyme PIG-B and GPI-anchored protein production in Drosophila. J Cell Sci. 2020 133(6): jcs238527. doi: 10.1242/jcs.238527 - Tanaka S, Maeda Y, Tashima Y, Kinoshita T. Inositol deacylation of glycosylphosphatidylinositol-anchored proteins is mediated by mammalian PGAP1 and yeast Bst1p. J Biol Chem. 2004 279(14): 14256-63. doi: 10.1074/jbc.M313755200 - Kolicheski AL, Johnson GS, Mhlanga-Mutangadura T, Taylor JF, Schnabel RD, Kinoshita T et al. A homozygous PIGN missense mutation in Soft-Coated Wheaten Terriers with a canine paroxysmal dyskinesia. Neurogenetics. 2017 18(1): 39-47. doi: 10.1007/s10048-016-0502-4 - Williamson NR, Simonsen HT, Ahmed RA, Goldet G, Slater H, Woodley L et al. Biosynthesis of the red antibiotic, prodigiosin, in Serratia: identification of a novel 2-methyl-3-n-amyl-pyrrole (MAP) assembly pathway, definition of the terminal condensing enzyme, and implications for undecylprodigiosin biosynthesis in Streptomyces. Mol Microbiol. 2005 56(4): 971-89. doi: 10.1111/j.1365-2958.2005.04602.x - Swain E, Stukey J, McDonough V, Germann M, Liu Y, Sturley SL et al. Yeast cells lacking the ARV1 gene harbor defects in sphingolipid metabolism. Complementation by human ARV1. J Biol Chem. 2002 277(39): 36152-60. doi: 10.1074/jbc.M206624200 - Murata D, Nomura KH, Dejima K, Mizuguchi S, Kawasaki N, Matsuishi-Nakajima Y et al. GPI-anchor synthesis is indispensable for the germline development of the nematode Caenorhabditis elegans. Mol Biol Cell. 2012 23(6): 982-95. doi: 10.1091/mbc.E10-10-0855 - Tinkelenberg AH, Liu Y, Alcantara F, Khan S, Guo Z, Bard M et al. Mutations in yeast ARV1 alter intracellular sterol distribution and are complemented by human ARV1. J Biol Chem. 2000 275(52): 40667-70. doi: 10.1074/jbc.C000710200 - Poplawsky AR, Chun W. pigB determines a diffusible factor needed for extracellular polysaccharide slime and xanthomonadin production in Xanthomonas campestris pv. campestris. J Bacteriol. 1997 179(2): 439-44. doi: 10.1128/jb.179.2.439-444.1997 - Romanowski EG, Lehner KM, Martin NC, Patel KR, Callaghan JD, Stella NA et al. Thermoregulation of Prodigiosin Biosynthesis by Serratia marcescens is Controlled at the Transcriptional Level and Requires HexS. Pol J Microbiol. 68(1): 43-50. doi: 10.21307/pjm-2019-005 - Fineran PC, Slater H, Everson L, Hughes K, Salmond GP. Biosynthesis of tripyrrole and beta-lactam secondary metabolites in Serratia: integration of quorum sensing with multiple new regulatory components in the control of prodigiosin and carbapenem antibiotic production. Mol Microbiol. 2005 56(6): 1495-517. doi: 10.1111/j.1365-2958.2005.04660.x - Beck PJ, Orlean P, Albright C, Robbins PW, Gething MJ, Sambrook JF. The Saccharomyces cerevisiae DPM1 gene encoding dolichol-phosphate-mannose synthase is able to complement a glycosylation-defective mammalian cell line. Mol Cell Biol. 1990 10(9): 4612-22. doi: 10.1128/mcb.10.9.4612-4622.1990 - Lukacs M, Blizzard LE, Stottmann RW. CNS glycosylphosphatidylinositol deficiency results in delayed white matter development, ataxia and premature death in a novel mouse model. Hum Mol Genet. 2020 29(7): 1205-1217. doi: 10.1093/hmg/ddaa046 - Saw WG, Eisenhaber B, Eisenhaber F, Grüber G. Low-resolution structure of the soluble domain GPAA1 (yGPAA170-247) of the glycosylphosphatidylinositol transamidase subunit GPAA1 from Saccharomyces cerevisiae. Biosci Rep. 2013 33(2): e00033. doi: 10.1042/BSR20120107 - Liu S, Ran T, Shen X, Xu L, Wang W, Xu D. Expression, crystallization and preliminary crystallographic data analysis of PigF, an O-methyltransferase from the prodigiosin-synthetic pathway in Serratia. Acta Crystallogr Sect F Struct Biol Cryst Commun. 2012 68(Pt 8): 898-901. doi: 10.1107/S1744309112024001 - Shin DJ, Choy HE, Hong Y. Use of Clostridium septicum alpha toxins for isolation of various glycosylphosphatidylinositol-deficient cells. J Microbiol. 2005 43(3): 266-71 - Tong F, Billheimer J, Shechtman CF, Liu Y, Crooke R, Graham M et al. Decreased expression of ARV1 results in cholesterol retention in the endoplasmic reticulum and abnormal bile acid metabolism. J Biol Chem. 2010 285(44): 33632-41. doi: 10.1074/jbc.M110.165761 - Oortveld MA, Keerthikumar S, Oti M, Nijhof B, Fernandes AC, Kochinke K et al. Human intellectual disability genes form conserved functional modules in Drosophila. PLoS Genet. 2013 9(10): e1003911. doi: 10.1371/journal.pgen.1003911 - Gristwood T, Fineran PC, Everson L, Salmond GP. PigZ, a TetR/AcrR family repressor, modulates secondary metabolism via the expression of a putative four-component resistance-nodulation-cell-division efflux pump, ZrpADBC, in Serratia sp. ATCC 39006. Mol Microbiol. 2008 69(2): 418-35. doi: 10.1111/j.1365-2958.2008.06291.x - Ghugtyal V, Vionnet C, Roubaty C, Conzelmann A. CWH43 is required for the introduction of ceramides into GPI anchors in Saccharomyces cerevisiae. Mol Microbiol. 2007 65(6): 1493-502. doi: 10.1111/j.1365-2958.2007.05883.x - Keller P, Tremml G, Rosti V, Bessler M. X inactivation and somatic cell selection rescue female mice carrying a Piga-null mutation. Proc Natl Acad Sci U S A. 1999 96(13): 7479-83. doi: 10.1073/pnas.96.13.7479 - Umemura M, Fujita M, Yoko-O T, Fukamizu A, Jigami Y. Saccharomyces cerevisiae CWH43 is involved in the remodeling of the lipid moiety of GPI anchors to ceramides. Mol Biol Cell. 2007 18(11): 4304-16. doi: 10.1091/mbc.e07-05-0482 - Kawaguchi K, Yamamoto-Hino M, Murakami Y, Kinoshita T, Goto S. Hrd1-dependent Degradation of the Unassembled PIGK Subunit of the GPI Transamidase Complex. Cell Struct Funct. 2021 46(2): 65-71. doi: 10.1247/csf.21019 - Hu R, Mukhina GL, Piantadosi S, Barber JP, Jones RJ, Brodsky RA. PIG-A mutations in normal hematopoiesis. Blood. 2005 105(10): 3848-54. doi: 10.1182/blood-2004-04-1472 - Sagane K, Umemura M, Ogawa-Mitsuhashi K, Tsukahara K, Yoko-o T, Jigami Y. Analysis of membrane topology and identification of essential residues for the yeast endoplasmic reticulum inositol acyltransferase Gwt1p. J Biol Chem. 2011 286(16): 14649-58. doi: 10.1074/jbc.M110.193490 - Ji Z, Tinti M, Ferguson MAJ. Proteomic identification of the UDP-GlcNAc: PI α1-6 GlcNAc-transferase subunits of the glycosylphosphatidylinositol biosynthetic pathway of Trypanosoma brucei. PLoS One. 2021 16(3): e0244699. doi: 10.1371/journal.pone.0244699 - Nagamune K, Ohishi K, Ashida H, Hong Y, Hino J, Kangawa K et al. GPI transamidase of Trypanosoma brucei has two previously uncharacterized (trypanosomatid transamidase 1 and 2) and three common subunits. Proc Natl Acad Sci U S A. 2003 100(19): 10682-7. doi: 10.1073/pnas.1833260100 - Hong Y, Ohishi K, Kang JY, Tanaka S, Inoue N, Nishimura J et al. Human PIG-U and yeast Cdc91p are the fifth subunit of GPI transamidase that attaches GPI-anchors to proteins. Mol Biol Cell. 2003 14(5): 1780-9. doi: 10.1091/mbc.e02-12-0794 - Ibeagha-Awemu EM, Li R, Ammah AA, Dudemaine PL, Bissonnette N, Benchaar C et al. Transcriptome adaptation of the bovine mammary gland to diets rich in unsaturated fatty acids shows greater impact of linseed oil over safflower oil on gene expression and metabolic pathways. BMC Genomics. 2016 17: 104. doi: 10.1186/s12864-016-2423-x - Tomita S, Inoue N, Maeda Y, Ohishi K, Takeda J, Kinoshita T. A homologue of Saccharomyces cerevisiae Dpm1p is not sufficient for synthesis of dolichol-phosphate-mannose in mammalian cells. J Biol Chem. 1998 273(15): 9249-54. doi: 10.1074/jbc.273.15.9249 - Rifai A, Wu CG. Clearance kinetics, tissue localization and fate of IgA-anti-idiotype complexes. Immunology. 1990 69(4): 610-5 - Liu S, Li Y, Deng B, Xu Z. Recombinant Lactococcus lactis expressing porcine insulin-like growth factor I ameliorates DSS-induced colitis in mice. BMC Biotechnol. 2016 16: 25. doi: 10.1186/s12896-016-0255-z - Hazenbos WL, Wu P, Eastham-Anderson J, Kinoshita T, Brown EJ. Impaired FcεRI stability, signaling, and effector functions in murine mast cells lacking glycosylphosphatidylinositol-anchored proteins. Blood. 2011 118(16): 4377-83. doi: 10.1182/blood-2011-02-338053 - Orlean P. Dolichol phosphate mannose synthase is required in vivo for glycosyl phosphatidylinositol membrane anchoring, O mannosylation, and N glycosylation of protein in Saccharomyces cerevisiae. Mol Cell Biol. 1990 10(11): 5796-805. doi: 10.1128/mcb.10.11.5796-5805.1990 - Ashida H, Hong Y, Murakami Y, Shishioh N, Sugimoto N, Kim YU et al. Mammalian PIG-X and yeast Pbn1p are the essential components of glycosylphosphatidylinositol-mannosyltransferase I. Mol Biol Cell. 2005 16(3): 1439-48. doi: 10.1091/mbc.e04-09-0802 - Yada T, Sugiura R, Kita A, Itoh Y, Lu Y, Hong Y et al. Its8, a fission yeast homolog of Mcd4 and Pig-n, is involved in GPI anchor synthesis and shares an essential function with calcineurin in cytokinesis. J Biol Chem. 2001 276(17): 13579-86. doi: 10.1074/jbc.M009260200 - Colussi PA, Taron CH, Mack JC, Orlean P. Human and Saccharomyces cerevisiae dolichol phosphate mannose synthases represent two classes of the enzyme, but both function in Schizosaccharomyces pombe. Proc Natl Acad Sci U S A. 1997 94(15): 7873-8. doi: 10.1073/pnas.94.15.7873 - Baulard AR, Gurcha SS, Engohang-Ndong J, Gouffi K, Locht C, Besra GS. In vivo interaction between the polyprenol phosphate mannose synthase Ppm1 and the integral membrane protein Ppm2 from Mycobacterium smegmatis revealed by a bacterial two-hybrid system. J Biol Chem. 2003 278(4): 2242-8. doi: 10.1074/jbc.M207922200 - Watanabe NA, Miyazaki M, Horii T, Sagane K, Tsukahara K, Hata K. E1210, a new broad-spectrum antifungal, suppresses Candida albicans hyphal growth through inhibition of glycosylphosphatidylinositol biosynthesis. Antimicrob Agents Chemother. 2012 56(2): 960-71. doi: 10.1128/AAC.00731-11 - Hara-Chikuma M, Takeda J, Tarutani M, Uchida Y, Holleran WM, Endo Y et al. Epidermal-specific defect of GPI anchor in Pig-a null mice results in Harlequin ichthyosis-like features. J Invest Dermatol. 2004 123(3): 464-9. doi: 10.1111/j.0022-202X.2004.23227.x - Cullen PJ, Schultz J, Horecka J, Stevenson BJ, Jigami Y, Sprague GF Jr. Defects in protein glycosylation cause SHO1-dependent activation of a STE12 signaling pathway in yeast. Genetics. 2000 155(3): 1005-18. doi: 10.1093/genetics/155.3.1005 - Basagoudanavar SH, Feng X, Krishnegowda G, Muthusamy A, Gowda DC. Plasmodium falciparum GPI mannosyltransferase-III has novel signature sequence and is functional. Biochem Biophys Res Commun. 2007 364(4): 748-54. doi: 10.1016/j.bbrc.2007 - Kapoor M, Moloney M, Soltow QA, Pillar CM, Shaw KJ. Evaluation of Resistance Development to the Gwt1 Inhibitor Manogepix (APX001A) in Candida Species. Antimicrob Agents Chemother. 2019 64(1): e01387-19. doi: 10.1128/AAC.01387-19 - Leidich SD, Kostova Z, Latek RR, Costello LC, Drapp DA, Gray W et al. Temperature-sensitive yeast GPI anchoring mutants gpi2 and gpi3 are defective in the synthesis of N-acetylglucosaminyl phosphatidylinositol. Cloning of the GPI2 gene. J Biol Chem. 1995 270(22): 13029-35. doi: 10.1074/jbc.270.22.13029 - Nagamune K, Nozaki T, Maeda Y, Ohishi K, Fukuma T, Hara T et al. Critical roles of glycosylphosphatidylinositol for Trypanosoma brucei. Proc Natl Acad Sci U S A. 2000 97(19): 10336-41. doi: 10.1073/pnas.180230697 - Taron CH, Wiedman JM, Grimme SJ, Orlean P. Glycosylphosphatidylinositol biosynthesis defects in Gpi11p- and Gpi13p-deficient yeast suggest a branched pathway and implicate gpi13p in phosphoethanolamine transfer to the third mannose. Mol Biol Cell. 2000 11(5): 1611-30. doi: 10.1091/mbc.11.5.1611 - Yamamoto-Hino M, Katsumata E, Suzuki E, Maeda Y, Kinoshita T, Goto S. Nuclear envelope localization of PIG-B is essential for GPI-anchor synthesis in Drosophila. J Cell Sci. 2018 131(20): jcs218024. doi: 10.1242/jcs.218024 - Kandasamy LC, Tsukamoto M, Banov V, Tsetsegee S, Nagasawa Y, Kato M et al. Limb-clasping, cognitive deficit and increased vulnerability to kainic acid-induced seizures in neuronal glycosylphosphatidylinositol deficiency mouse models. Hum Mol Genet. 2021 30(9): 758-770. doi: 10.1093/hmg/ddab052 - Tremml G, Dominguez C, Rosti V, Zhang Z, Pandolfi PP, Keller P et al. Increased sensitivity to complement and a decreased red blood cell life span in mice mosaic for a nonfunctional Piga gene. Blood. 1999 94(9): 2945-54 - Dai X, Gao G, Wu M, Wei W, Qu J, Li G et al. Construction and application of a Xanthomonas campestris CGMCC15155 strain that produces white xanthan gum. Microbiologyopen. 2019 8(2): e00631. doi: 10.1002/mbo3.631 - Björkholm P, Ernst AM, Hacke M, Wieland F, Brügger B, von Heijne G. Identification of novel sphingolipid-binding motifs in mammalian membrane proteins. Biochim Biophys Acta. 2014 1838(8): 2066-70. doi: 10.1016/j.bbamem.2014.04.026 - Kim YU, Hong Y. Functional analysis of the first mannosyltransferase (PIG-M) involved in glycosylphosphatidylinositol synthesis in Plasmodium falciparum. Mol Cells. 2007 24(2): 294-300 - Tiede A, Schubert J, Nischan C, Jensen I, Westfall B, Taron CH, Orlean P, Schmidt RE. Human and mouse Gpi1p homologues restore glycosylphosphatidylinositol membrane anchor biosynthesis in yeast mutants. Biochem J. 1998 334 ( Pt 3)(Pt 3): 609-16. doi: 10.1042/bj3340609 - Tanikawa T, Nakagawa Y, Matsuyama T. Transcriptional downregulator hexS controlling prodigiosin and serrawettin W1 biosynthesis in Serratia marcescens. Microbiol Immunol. 2006 50(8): 587-96. doi: 10.1111/j.1348-0421.2006.tb03833.x - Orlean P, Albright C, Robbins PW. Cloning and sequencing of the yeast gene for dolichol phosphate mannose synthase, an essential protein. J Biol Chem. 1988 263(33): 17499-507 - Delorenzi M, Sexton A, Shams-Eldin H, Schwarz RT, Speed T, Schofield L. Genes for glycosylphosphatidylinositol toxin biosynthesis in Plasmodium falciparum. Infect Immun. 2002 70(8): 4510-22. doi: 10.1128/IAI.70.8.4510-4522.2002 - Kawagoe K, Kitamura D, Okabe M, Taniuchi I, Ikawa M, Watanabe T et al. Glycosylphosphatidylinositol-anchor-deficient mice: implications for clonal dominance of mutant cells in paroxysmal nocturnal hemoglobinuria. Blood. 1996 87(9): 3600-6 - Zakrzewska A, Palamarczyk G, Krotkiewski H, Zdebska E, Saloheimo M, Penttilä M et al. Overexpression of the gene encoding GTP:mannose-1-phosphate guanyltransferase, mpg1, increases cellular GDP-mannose levels and protein mannosylation in Trichoderma reesei. Appl Environ Microbiol. 2003 69(8): 4383-9. doi: 10.1128/AEM.69.8.4383-4389.2003 - Wegele R, Tasler R, Zeng Y, Rivera M, Frankenberg-Dinkel N. The heme oxygenase(s)-phytochrome system of Pseudomonas aeruginosa. J Biol Chem. 2004 279(44): 45791-802. doi: 10.1074/jbc.M408303200 - Gillmor CS, Lukowitz W, Brininstool G, Sedbrook JC, Hamann T, Poindexter P et al. Glycosylphosphatidylinositol-anchored proteins are required for cell wall synthesis and morphogenesis in Arabidopsis. Plant Cell. 2005 17(4): 1128-40. doi: 10.1105/tpc.105.031815 - Morita YS, Sena CB, Waller RF, Kurokawa K, Sernee MF, Nakatani F et al. PimE is a polyprenol-phosphate-mannose-dependent mannosyltransferase that transfers the fifth mannose of phosphatidylinositol mannoside in mycobacteria. J Biol Chem. 2006 281(35): 25143-55. doi: 10.1074/jbc.M604214200 - Toyn JH, Lin XA, Thompson MW, Guss V, Meredith JE Jr, Sankaranarayanan S et al. Viable mouse gene ablations that robustly alter brain Aβ levels are rare. BMC Neurosci. 2010 11: 143. doi: 10.1186/1471-2202-11-143 - Murakami Y, Kinoshita T, Maeda Y, Nakano T, Kosaka H, Takeda J. Different roles of glycosylphosphatidylinositol in various hematopoietic cells as revealed by a mouse model of paroxysmal nocturnal hemoglobinuria. Blood. 1999 94(9): 2963-70 - Hong Y, Maeda Y, Watanabe R, Ohishi K, Mishkind M, Riezman H et al. Pig-n, a mammalian homologue of yeast Mcd4p, is involved in transferring phosphoethanolamine to the first mannose of the glycosylphosphatidylinositol. J Biol Chem. 1999 274(49): 35099-106. doi: 10.1074/jbc.274.49.35099 - Perlińska-Lenart U, Kurzatkowski W, Janas P, Kopińska A, Palamarczyk G, Kruszewska JS. Protein production and secretion in an Aspergillus nidulans mutant impaired in glycosylation. Acta Biochim Pol. 52(1): 195-206 - Yano J, Rachochy V, Van Houten JL. Glycosyl phosphatidylinositol-anchored proteins in chemosensory signaling: antisense manipulation of Paramecium tetraurelia PIG-A gene expression. Eukaryot Cell. 2003 2(6): 1211-9. doi: 10.1128/EC.2.6.1211-1219.2003 - Perlińska-Lenart U, Orlowski J, Laudy AE, Zdebska E, Palamarczyk G, Kruszewska JS. Glycoprotein hypersecretion alters the cell wall in Trichoderma reesei strains expressing the Saccharomyces cerevisiae dolichylphosphate mannose synthase gene. Appl Environ Microbiol. 2006 72(12): 7778-84. doi: 10.1128/AEM.02375-06 - Slater H, Crow M, Everson L, Salmond GP. Phosphate availability regulates biosynthesis of two antibiotics, prodigiosin and carbapenem, in Serratia via both quorum-sensing-dependent and -independent pathways. Mol Microbiol. 2003 47(2): 303-20. doi: 10.1046/j.1365-2958.2003.03295.x - Lillico S, Field MC, Blundell P, Coombs GH, Mottram JC. Essential roles for GPI-anchored proteins in African trypanosomes revealed using mutants deficient in GPI8. Mol Biol Cell. 2003 14(3): 1182-94. doi: 10.1091/mbc.e02-03-0167 - Garay E, Campos SE, González de la Cruz J, Gaspar AP, Jinich A, Deluna A. High-resolution profiling of stationary-phase survival reveals yeast longevity factors and their genetic interactions. PLoS Genet. 2014 10(2): e1004168. doi: 10.1371/journal.pgen - Jonard PP, Rambaud JC, Dive C, Vaerman JP, Galian A, Delacroix DL. Secretion of immunoglobulins and plasma proteins from the jejunal mucosa. Transport rate and origin of polymeric immunoglobulin A. J Clin Invest. 1984 74(2): 525-35. doi: 10.1172/JCI111450 - Renegar KB, Kudsk KA, Dewitt RC, Wu Y, King BK. Impairment of mucosal immunity by parenteral nutrition: depressed nasotracheal influenza-specific secretory IgA levels and transport in parenterally fed mice. Ann Surg. 2001 233(1): 134-8. doi: 10.1097/00000658-200101000-00019 - Wiedman JM, Fabre AL, Taron BW, Taron CH, Orlean P. In vivo characterization of the GPI assembly defect in yeast mcd4-174 mutants and bypass of the Mcd4p-dependent step in mcd4Delta cells. FEMS Yeast Res. 2007 7(1): 78-83. doi: 10.1111/j.1567-1364.2006.00139.x - Chang T, Milne KG, Güther ML, Smith TK, Ferguson MA. Cloning of Trypanosoma brucei and Leishmania major genes encoding the GlcNAc-phosphatidylinositol de-N-acetylase of glycosylphosphatidylinositol biosynthesis that is essential to the African sleeping sickness parasite. J Biol Chem. 2002 277(51): 50176-82. doi: 10.1074/jbc.M208374200 - Hoppe CA, Connolly TP, Hubbard AL. Transcellular transport of polymeric IgA in the rat hepatocyte: biochemical and morphological characterization of the transport pathway. J Cell Biol. 1985 101(6): 2113-23. doi: 10.1083/jcb.101.6.2113 - Limet JN, Quintart J, Schneider YJ, Courtoy PJ. Receptor-mediated endocytosis of polymeric IgA and galactosylated serum albumin in rat liver. Evidence for intracellular ligand sorting and identification of distinct endosomal compartments. Eur J Biochem. 1985 146(3): 539-48. doi: 10.1111/j.1432-1033.1985.tb08685.x - Banerjee DK, Carrasquillo EA, Hughey P, Schutzbach JS, Martínez JA, Baksi K. In vitro phosphorylation by cAMP-dependent protein kinase up-regulates recombinant Saccharomyces cerevisiae mannosylphosphodolichol synthase. J Biol Chem. 2005 280(6): 4174-81. doi: 10.1074/jbc.M406962200 - Graupner A, Eide DM, Instanes C, Andersen JM, Brede DA, Dertinger SD et al. Gamma radiation at a human relevant low dose rate is genotoxic in mice. Sci Rep. 2016 6: 32977. doi: 10.1038/srep32977 - Vats D, Vishwakarma RA, Bhattacharya S, Bhattacharya A. Reduction of cell surface glycosylphosphatidylinositol conjugates in Entamoeba histolytica by antisense blocking of E. histolytica GlcNAc-phosphatidylinositol deacetylase expression: effect on cell proliferation, endocytosis, and adhesion to target cells. Infect Immun. 2005 73(12): 8381-92. doi: 10.1128/IAI.73.12.8381-8392.2005 - Grabińska K, Sosińska G, Orłowski J, Swiezewska E, Berges T, Karst F et al. Functional relationships between the Saccharomyces cerevisiae cis-prenyltransferases required for dolichol biosynthesis. Acta Biochim Pol. 2005 52(1): 221-32 - Plavner N, Eichler J. Defining the topology of the N-glycosylation pathway in the halophilic archaeon Haloferax volcanii. J Bacteriol. 2008 190(24): 8045-52. doi: 10.1128/JB.01200-08 - Han N, Ran T, Lou X, Gao Y, He J, Tang L et al. Expression, crystallization and preliminary crystallographic data analysis of PigI, a putative L-prolyl-AMP ligase from the prodigiosin synthetic pathway in Serratia. Acta Crystallogr F Struct Biol Commun. 2014 70(Pt 5): 624-7. doi: 10.1107/S2053230X14005780 - Kruszewska JS, Butterweck AH, Kurzatkowski W, Migdalski A, Kubicek CP, Palamarczyk G. Overexpression of the Saccharomyces cerevisiae mannosylphosphodolichol synthase-encoding gene in Trichoderma reesei results in an increased level of protein secretion and abnormal cell ultrastructure. Appl Environ Microbiol. 1999 65(6): 2382-7. doi: 10.1128/AEM.65.6.2382-2387.1999 - Albright CF, Orlean P, Robbins PW. A 13-amino acid peptide in three yeast glycosyltransferases may be involved in dolichol recognition. Proc Natl Acad Sci U S A. 1989 86(19): 7366-9. doi: 10.1073/pnas.86.19.7366 - Watanabe R, Ohishi K, Maeda Y, Nakamura N, Kinoshita T. Mammalian PIG-L and its yeast homologue Gpi12p are N-acetylglucosaminylphosphatidylinositol de-N-acetylases essential in glycosylphosphatidylinositol biosynthesis. Biochem J. 1999 339 ( Pt 1)(Pt 1): 185-92 - Taron BW, Colussi PA, Wiedman JM, Orlean P, Taron CH. Human Smp3p adds a fourth mannose to yeast and human glycosylphosphatidylinositol precursors in vivo. J Biol Chem. 2004 279(34): 36083-92. doi: 10.1074/jbc.M405081200 - Wichroski MJ, Ward GE. Biosynthesis of glycosylphosphatidylinositol is essential to the survival of the protozoan parasite Toxoplasma gondii. Eukaryot Cell. 2003 2(5): 1132-6. doi: 10.1128/EC.2.5.1132-1136.2003 - Hong Y, Nagamune K, Ohishi K, Morita YS, Ashida H, Maeda Y et al. TbGPI16 is an essential component of GPI transamidase in Trypanosoma brucei. FEBS Lett. 2006 580(2): 603-6. doi: 10.1016/j.febslet.2005.12.075 - Mahal SP, Jablonski J, Suponitsky-Kroyter I, Oelschlegel AM, Herva ME, Oldstone M et al. Propagation of RML prions in mice expressing PrP devoid of GPI anchor leads to formation of a novel, stable prion strain. PLoS Pathog. 2012 8(6): e1002746. doi: 10.1371/journal.ppat.1002746 - De Sampaïo G, Bourdineaud JP, Lauquin GJ. A constitutive role for GPI anchors in Saccharomyces cerevisiae: cell wall targeting. Mol Microbiol. 1999 34(2): 247-56. doi: 10.1046/j.1365-2958.1999.01585.x - Newman HA, Romeo MJ, Lewis SE, Yan BC, Orlean P, Levin DE. Gpi19, the Saccharomyces cerevisiae homologue of mammalian PIG-P, is a subunit of the initial enzyme for glycosylphosphatidylinositol anchor biosynthesis. Eukaryot Cell. 2005 4(11): 1801-7. doi: 10.1128/EC.4.11.1801-1807.2005 - Schönbächler M, Horvath A, Fassler J, Riezman H. The yeast spt14 gene is homologous to the human PIG-A gene and is required for GPI anchor synthesis. EMBO J. 1995 14(8): 1637-45 - Tarutani M, Itami S, Okabe M, Ikawa M, Tezuka T, Yoshikawa K et al. Tissue-specific knockout of the mouse Pig-a gene reveals important roles for GPI-anchored proteins in skin development. Proc Natl Acad Sci U S A. 1997 94(14): 7400-5. doi: 10.1073/pnas.94.14.7400 - Abu-Qarn M, Giordano A, Battaglia F, Trauner A, Hitchen PG, Morris HR et al. Identification of AglE, a second glycosyltransferase involved in N glycosylation of the Haloferax volcanii S-layer glycoprotein. J Bacteriol. 2008 190(9): 3140-6. doi: 10.1128/JB.00056-08 - Daddaoua A, Krell T, Ramos JL. Regulation of glucose metabolism in Pseudomonas: the phosphorylative branch and entner-doudoroff enzymes are regulated by a repressor containing a sugar isomerase domain. J Biol Chem. 2009 284(32): 21360-8. doi: 10.1074/jbc.M109.014555 - Meyer U, Fraering P, Bosson R, Imhof I, Benghezal M, Vionnet C et al. The glycosylphosphatidylinositol (GPI) signal sequence of human placental alkaline phosphatase is not recognized by human Gpi8p in the context of the yeast GPI anchoring machinery. Mol Microbiol. 2002 46(3): 745-8. doi: 10.1046/j.1365-2958.2002.03192.x - Hodgson DA, Shaw P, Shapiro L. Isolation and genetic analysis of Caulobacter mutants defective in cell shape and membrane lipid synthesis. Genetics. 1984 108(4): 809-26. doi: 10.1093/genetics/108.4.809 - Ware FE, Lehrman MA. Expression cloning of a novel suppressor of the Lec15 and Lec35 glycosylation mutations of Chinese hamster ovary cells. J Biol Chem. 1996 271(24): 13935-8. doi: 10.1074/jbc.271.24.13935 - Benghezal M, Benachour A, Rusconi S, Aebi M, Conzelmann A. Yeast Gpi8p is essential for GPI anchor attachment onto proteins. EMBO J. 1996 15(23): 6575-83 - Axelsson K, Johansson S, Eketorp G, Zazzi H, Hemmendorf B, Gellerfors P. Disulfide arrangement of human insulin-like growth factor I derived from yeast and plasma. Eur J Biochem. 1992 206(3): 987-94. doi: 10.1111/j.1432-1033.1992.tb17010.x - Ashraf M, Sreejith P, Yadav U, Komath SS. Catalysis by N-acetyl-D-glucosaminylphosphatidylinositol de-N-acetylase (PIG-L) from Entamoeba histolytica: new roles for conserved residues. J Biol Chem. 2013 288(11): 7590-7595. doi: 10.1074/jbc.M112.427245 - Sütterlin C, Escribano MV, Gerold P, Maeda Y, Mazon MJ, Kinoshita T et al. Saccharomyces cerevisiae GPI10, the functional homologue of human PIG-B, is required for glycosylphosphatidylinositol-anchor synthesis. Biochem J. 1998 332 ( Pt 1)(Pt 1): 153-9. doi: 10.1042/bj3320153 - Hiroi Y, Komuro I, Chen R, Hosoda T, Mizuno T, Kudoh S et al. Molecular cloning of human homolog of yeast GAA1 which is required for attachment of glycosylphosphatidylinositols to proteins. FEBS Lett. 1998 421(3): 252-8. doi: 10.1016/s0014-5793(97)01576-7 - Weber C, Blazquez S, Marion S, Ausseur C, Vats D, Krzeminski M et al. Bioinformatics and functional analysis of an Entamoeba histolytica mannosyltransferase necessary for parasite complement resistance and hepatical infection. PLoS Negl Trop Dis. 2008 2(2): e165. doi: 10.1371/journal.pntd.0000165 - Speed RR, Winkler HH. Acquisition of thymidylate by the obligate intracytoplasmic bacterium Rickettsia prowazekii. J Bacteriol. 1991 173(5): 1704-10. doi: 10.1128/jb.173.5.1704-1710.1991 - Graupner A, Instanes C, Andersen JM, Brandt-Kjelsen A, Dertinger SD, Salbu B et al. Genotoxic effects of two-generational selenium deficiency in mouse somatic and testicular cells. Mutagenesis. 2015 30(2): 217-25. doi: 10.1093/mutage/geu059 - Ashraf M, Yadav B, Perinthottathil S, Kumar KS, Vats D, Muthuswami R et al. N-acetyl-D-glucosaminylphosphatidylinositol de-N-acetylase from Entamoeba histolytica: metal alters catalytic rates but not substrate affinity. J Biol Chem. 2011 286(4): 2543-9. doi: 10.1074/jbc.C110.178343 - Bouwman LMS, Nieuwenhuizen AG, Swarts HJM, Piga R, van Schothorst EM, Keijer J. Metabolic effects of the dietary monosaccharides fructose, fructose-glucose, or glucose in mice fed a starch-containing moderate high-fat diet. Physiol Rep. 2020 8(3): e14350. doi: 10.14814/phy2.14350 - Jiang F, Wang X, Wang B, Chen L, Zhao Z, Waterfield NR et al. The Pseudomonas aeruginosa Type VI Secretion PGAP1-like Effector Induces Host Autophagy by Activating Endoplasmic Reticulum Stress. Cell Rep. 2016 16(6): 1502-1509. doi: 10.1016/j.celrep.2016.07.012 - Pan X, Sun C, Tang M, You J, Osire T, Zhao Y et al. LysR-Type Transcriptional Regulator MetR Controls Prodigiosin Production, Methionine Biosynthesis, Cell Motility, H2O2 Tolerance, Heat Tolerance, and Exopolysaccharide Synthesis in Serratia marcescens. Appl Environ Microbiol. 2020 86(4): e02241-19. doi: 10.1128/AEM.02241-19 - Grabińska KA, Ghosh SK, Guan Z, Cui J, Raetz CR, Robbins PW et al. Dolichyl-phosphate-glucose is used to make O-glycans on glycoproteins of Trichomonas vaginalis. Eukaryot Cell. 2008 7(8): 1344-51. doi: 10.1128/EC.00061-08 - Ferrando ML, van Baarlen P, Orrù G, Piga R, Bongers RS, Wels M et al. Carbohydrate availability regulates virulence gene expression in Streptococcus suis. PLoS One. 2014 9(3): e89334. doi: 10.1371/journal.pone.0089334 - Chick WS, Ludwig M, Zhao X, Kitzenberg D, Williams K, Johnson TE. Screening for stress-resistance mutations in the mouse. Front Genet. 2014 5: 310. doi: 10.3389/fgene.2014.00310 - Zembek P, Perlinska-Lenart U, Brunner K, Reithner B, Palamarczyk G, Mach RL. Elevated activity of dolichyl phosphate mannose synthase enhances biocontrol abilities of Trichoderma atroviride. Mol Plant Microbe Interact. 2011 24(12): 1522-9. doi: 10.1094/MPMI-02-11-0025 - Harmatz PR, Carter EA, Sullivan D, Hatz RA, Baker R, Breazeale E et al. Effect of thermal injury in the rat on transfer of IgA protein into bile. Ann Surg. 1989 210(2): 203-7. doi: 10.1097/00000658-198908000-00011 - Das SK, Barhwal K, Hota SK, Thakur MK, Srivastava RB. Disrupting monotony during social isolation stress prevents early development of anxiety and depression like traits in male rats. BMC Neurosci. 2015 16: 2. doi: 10.1186/s12868-015-0141-y - Gao J, Xu D, Sabat G, Valdivia H, Xu W, Shi NQ. Disrupting KATP channels diminishes the estrogen-mediated protection in female mutant mice during ischemia-reperfusion. Clin Proteomics. 2014 11(1): 19. doi: 10.1186/1559-0275-11-19 - Gley K, Murani E, Trakooljul N, Zebunke M, Puppe B, Wimmers K et al. Transcriptome profiles of hypothalamus and adrenal gland linked to haplotype related to coping behavior in pigs. Sci Rep. 2019 9(1): 13038. doi: 10.1038/s41598-019-49521-2 - Ikeda K, Mason PJ, Bessler M. 3'UTR-truncated Hmga2 cDNA causes MPN-like hematopoiesis by conferring a clonal growth advantage at the level of HSC in mice. Blood. 2011 117(22): 5860-9. doi: 10.1182/blood-2011-02-334425 - Marteau P, Vaerman JP, Dehennin JP, Bord S, Brassart D, Pochart P et al. Effects of intrajejunal perfusion and chronic ingestion of Lactobacillus johnsonii strain La1 on serum concentrations and jejunal secretions of immunoglobulins and serum proteins in healthy humans. Gastroenterol Clin Biol. 1997 21(4): 293-8 - MacFarlane AJ, Behan NA, Field MS, Williams A, Stover PJ, Yauk CL. Dietary folic acid protects against genotoxicity in the red blood cells of mice. Mutat Res. 2015 779: 105-11. doi: 10.1016/j.mrfmmm.2015.06.012 - Kobayashi A, Donaldson DS, Kanaya T, Fukuda S, Baillie JK, Freeman TC et al. Identification of novel genes selectively expressed in the follicle-associated epithelium from the meta-analysis of transcriptomics data from multiple mouse cell and tissue populations. DNA Res. 2012 19(5): 407-22. doi: 10.1093/dnares/dss022 - Lorrai I, Piga V, Carai MA, Riva A, Morazzoni P, Gessa GL et al. A Phaseolus vulgaris Extract Reduces Cue-Induced Reinstatement of Chocolate Seeking in Rats. Front Pharmacol. 2016 7: 109. doi: 10.3389/fphar.2016.00109 - Wu Y, Chen X, Zhou Q, He Q, Kang J, Zheng J et al. ITE and TCDD differentially regulate the vascular remodeling of rat placenta via the activation of AhR. PLoS One. 2014 9(1): e86549. doi: 10.1371/journal.pone.0086549 - Srinivasan R, Mohankumar R, Kannappan A, Karthick Raja V, Archunan G, Karutha Pandian S et al. Exploring the Anti-quorum Sensing and Antibiofilm Efficacy of Phytol against Serratia marcescens Associated Acute Pyelonephritis Infection in Wistar Rats. Front Cell Infect Microbiol. 2017 7: 498. doi: 10.3389/fcimb.2017.00498 - McKinley ET, Bugaj JE, Zhao P, Guleryuz S, Mantis C, Gokhale PC et al. 18FDG-PET predicts pharmacodynamic response to OSI-906, a dual IGF-1R/IR inhibitor, in preclinical mouse models of lung cancer. Clin Cancer Res. 2011 17(10): 3332-40. doi: 10.1158/1078-0432.CCR-10-2274 |
| Studies describing disorders other than GPIBD | - Daan NM, Koster MP, de Wilde MA, Dalmeijer GW, Evelein AM, Fauser BC et al. Biomarker Profiles in Women with PCOS and PCOS Offspring; A Pilot Study. PLoS One. 2016 11(11): e0165033. doi: 10.1371/journal.pone.0165033 - Brodsky RA. Paroxysmal nocturnal hemoglobinuria without GPI-anchor deficiency. J Clin Invest. 2019 129(12): 5074-5076. doi: 10.1172/JCI131647 - Höchsmann B, Murakami Y, Osato M, Knaus A, Kawamoto M, Inoue N et al. Complement and inflammasome overactivation mediates paroxysmal nocturnal hemoglobinuria with autoinflammation. J Clin Invest. 2019 129(12): 5123-5136. doi: 10.1172/JCI123501 - Borowitz MJ, Craig FE, Digiuseppe JA, Illingworth AJ, Rosse W, Sutherland DR et al. Guidelines for the diagnosis and monitoring of paroxysmal nocturnal hemoglobinuria and related disorders by flow cytometry. Cytometry B Clin Cytom. 2010 78(4): 211-30. doi: 10.1002/cyto.b.20525 - Zhang XX, Ni B, Li Q, Hu LP, Jiang SH, Li RK et al. GPAA1 promotes gastric cancer progression via upregulation of GPI-anchored protein and enhancement of ERBB signalling pathway. J Exp Clin Cancer Res. 2019 38(1): 214. doi: 10.1186/s13046-019-1218-8 - Ho JC, Cheung ST, Patil M, Chen X, Fan ST. Increased expression of glycosyl-phosphatidylinositol anchor attachment protein 1 (GPAA1) is associated with gene amplification in hepatocellular carcinoma. Int J Cancer. 2006 119(6): 1330-7. doi: 10.1002/ijc.22005 - Hu R, Mukhina GL, Lee SH, Jones RJ, Englund PT, Brown P et al. Silencing of genes required for glycosylphosphatidylinositol anchor biosynthesis in Burkitt lymphoma. Exp Hematol. 2009 37(4): 423-434.e2. doi: 10.1016/j.exphem.2009.01.003 - Chen G, Li SY, Cai HY, Zuo FY. Enhanced expression and significance of glycosylphosphatidylinositol anchor attachment protein 1 in colorectal cancer. Genet Mol Res. 2014 13(1): 499-507. doi: 10.4238/2014.January.21.19 - Sun M, Qiu J, Zhai H, Wang Y, Ma P, Li M et al. Prognostic Implications of Novel Gene Signatures in Gastric Cancer Microenvironment. Med Sci Monit. 2020 26: e924604. doi: 10.12659/MSM.924604 - Loeff FC, Rijs K, van Egmond EHM, Zoutman WH, Qiao X, Kroes WGM et al. Loss of the GPI-anchor in B-lymphoblastic leukemia by epigenetic downregulation of PIGH expression. Am J Hematol. 2019 94(1): 93-102. doi: 10.1002/ajh.25337 - Zhang JX, Wang JH, Sun XG, Hou TZ. GPAA1 promotes progression of childhood acute lymphoblastic leukemia through regulating c-myc. Eur Rev Med Pharmacol Sci. 2020 24(9): 4931-4939. doi: 10.26355/eurrev_202005_21182 - Peng X, Lei C, He A, Luo R, Cai Y, Dong W. Upregulation of phosphatidylinositol glycan anchor biosynthesis class C is associated with unfavorable survival prognosis in patients with hepatocellular carcinoma. Oncol Lett. 2021 21(3): 237. doi: 10.3892/ol.2021.12498 - Kim P, Scott MR, Meador-Woodruff JH. Abnormal ER quality control of neural GPI-anchored proteins via dysfunction in ER export processing in the frontal cortex of elderly subjects with schizophrenia. Transl Psychiatry. 2019 9(1): 6. doi: 10.1038/s41398-018-0359-4 - Labeau A, Simon-Loriere E, Hafirassou ML, Bonnet-Madin L, Tessier S, Zamborlini A, Dupré T, Seta N, Schwartz O, Chaix ML, Delaugerre C, Amara A, Meertens L. A Genome-Wide CRISPR-Cas9 Screen Identifies the Dolichol-Phosphate Mannose Synthase Complex as a Host Dependency Factor for Dengue Virus Infection. J Virol. 2020 94(7): e01751-19. doi: 10.1128/JVI.01751-19 - Cho HY, Kyung MS. CYFRA 21-1 and Placental Growth Factor as Screening Markers for Endometriosis. Med Sci Monit. 2019 25: 1087-1092. doi: 10.12659/MSM.912787 - Teye EK, Sido A, Xin P, Finnberg NK, Gokare P, Kawasawa YI et al. PIGN gene expression aberration is associated with genomic instability and leukemic progression in acute myeloid leukemia with myelodysplastic features. Oncotarget. 2017 8(18): 29887-29905. doi: 10.18632/oncotarget.15136 - Fiumara A, Barone R, Del Campo G, Striano P, Jaeken J. Electroclinical Features of Early-Onset Epileptic Encephalopathies in Congenital Disorders of Glycosylation (CDGs). JIMD Rep. 27: 93-9. doi: 10.1007/8904_2015_497 - Zhao P, Nairn AV, Hester S, Moremen KW, O'Regan RM, Oprea G et al. Proteomic identification of glycosylphosphatidylinositol anchor-dependent membrane proteins elevated in breast carcinoma. J Biol Chem. 2012 287(30): 25230-40. doi: 10.1074/jbc.M112.339465 - Magalhães APPS, Burin MG, Souza CFM, de Bitencourt FH, Sebastião FM, Silva TO et al. Transferrin isoelectric focusing for the investigation of congenital disorders of glycosylation: analysis of a ten-year experience in a Brazilian center. J Pediatr (Rio J). 2020 96(6): 710-716. doi: 10.1016/j.jped.2019.05.008 - Lodhi OUH, Sohail S, Hassan D. A Rare Case of Paroxysmal Nocturnal Hemoglobinuria With Bilateral Renal Vein Thrombosis. Cureus. 2020 12(6): e8806. doi: 10.7759/cureus.8806 - Ware RE, Howard TA, Kamitani T, Chang H-M, Yeh ETH, Seldin MF. Chromosomal assignment of genes involved in glycosylphosphatidylinositol anchor biosynthesis: implications for the pathogenesis of paroxysmal nocturnal hemoglobinuria. Blood. 1994 83(12): 3753-7 - Costa JR, Caputo VS, Makarona K, Layton DM, Roberts IA, Almeida AM et al. Cell-type-specific transcriptional regulation of PIGM underpins the divergent hematologic phenotype in inherited GPl deficiency. Blood. 2014 124(20): 3151-4. doi: 10.1182/blood-2014-09-598813. - Nagpal JK, Dasgupta S, Jadallah S, Chae YK, Ratovitski EA, Toubaji A et al. Profiling the expression pattern of GPI transamidase complex subunits in human cancer. Mod Pathol. 2008 21(8): 979-91. doi: 10.1038/modpathol.2008.76 - Wu G, Guo Z, Chatterjee A, Huang X, Rubin E, Wu F et al. Overexpression of glycosylphosphatidylinositol (GPI) transamidase subunits phosphatidylinositol glycan class T and/or GPI anchor attachment 1 induces tumorigenesis and contributes to invasion in human breast cancer. Cancer Res. 2006 66(20): 9829-36. doi: 10.1158/0008-5472.CAN-06-0506 - Palau VE, Chakraborty K, Wann D, Lightner J, Hilton K, Brannon M et al. γ-Tocotrienol induces apoptosis in pancreatic cancer cells by upregulation of ceramide synthesis and modulation of sphingolipid transport. BMC Cancer. 2018 18(1): 564. doi: 10.1186/s12885-018-4462-y - Langemeijer S, Schaap C, Preijers F, Jansen JH, Blijlevens N, Inoue N. Paroxysmal nocturnal hemoglobinuria caused by CN-LOH of constitutional PIGB mutation and 70-kbp microdeletion on 15q. Blood Adv. 2020 4(22): 5755-5761. doi: 10.1182/bloodadvances.2020002210 - Schleinitz D, Klöting N, Lindgren CM, Breitfeld J, Dietrich A, Schön MR et al. Fat depot-specific mRNA expression of novel loci associated with waist-hip ratio. Int J Obes (Lond). 2014 38(1): 120-5. doi: 10.1038/ijo.2013.56 - Park J, Kim M, Kim Y, Han K, Chung NG, Cho B et al. Clonal Cell Proliferation in Paroxysmal Nocturnal Hemoglobinuria: Evaluation of PIGA Mutations and T-cell Receptor Clonality. Ann Lab Med. 2019 39(5): 438-446. doi: 10.3343/alm.2019.39.5.438 - Chang Y, He J, Tang J, Chen K, Wang Z, Xia Q et al. Investigation of the gene co-expression network and hub genes associated with acute mountain sickness. Hereditas. 2020 157(1): 13. doi: 10.1186/s41065-020-00127-z - Zhang A, Zhou LF, Xiang XL, Wang K, Zhou Q, Duan T. Folic acid attenuates dexamethasone-induced placental growth restriction. Eur Rev Med Pharmacol Sci. 2015 19(7): 1130-40 - Horne AW, Shaw JL, Murdoch A, McDonald SE, Williams AR, Jabbour HN et al. Placental growth factor: a promising diagnostic biomarker for tubal ectopic pregnancy. J Clin Endocrinol Metab. 2011 96(1): E104-8. doi: 10.1210/jc.2010-1403 - Guo X, Tian S, Cao P, Xie Y, Dong W. High Expression of PIGC Predicts Unfavorable Survival in Hepatocellular Carcinoma. J Hepatocell Carcinoma. 2021 8: 211-222. doi: 10.2147/JHC.S297601 - Robak E, Kulczycka L, Sysa-Jedrzejowska A, Wierzbowska A, Robak T. Circulating proangiogenic molecules PIGF, SDF-1 and sVCAM-1 in patients with systemic lupus erythematosus. Eur Cytokine Netw. 2007 18(4): 181-7. doi: 10.1684/ecn.2007.0103 - Agrahari AK, Pieroni E, Gatto G, Kumar A. The impact of missense mutation in PIGA associated to paroxysmal nocturnal hemoglobinuria and multiple congenital anomalies-hypotonia-seizures syndrome 2: A computational study. Heliyon. 2019 5(10): e02709. doi: 10.1016/j.heliyon.2019.e02709 - Söderman J, Berglind L, Almer S. Gene Expression-Genotype Analysis Implicates GSDMA, GSDMB, and LRRC3C as Contributors to Inflammatory Bowel Disease Susceptibility. Biomed Res Int. 2015: 834805. doi: 10.1155/2015/834805 - Rana M, Dong J, Robertson MJ, Basil P, Coarfa C, Weigel NL. Androgen receptor and its splice variant, AR-V7, differentially induce mRNA splicing in prostate cancer cells. Sci Rep. 2021 11(1): 1393. doi: 10.1038/s41598-021-81164-0 - Johnston JJ, Gropman AL, Sapp JC, Teer JK, Martin JM, Liu CF et al. The phenotype of a germline mutation in PIGA: the gene somatically mutated in paroxysmal nocturnal hemoglobinuria. Am J Hum Genet. 2012 90(2): 295-300. doi: 10.1016/j.ajhg.2011.11.031 - Alessandri JL, Gordon CT, Jacquemont ML, Gruchy N, Ajeawung NF, Benoist G et al. Recessive loss of function PIGN alleles, including an intragenic deletion with founder effect in La Réunion Island, in patients with Fryns syndrome. Eur J Hum Genet. 2018 26(3): 340-349. doi: 10.1038/s41431-017-0087-x - Krawitz PM, Höchsmann B, Murakami Y, Teubner B, Krüger U, Klopocki E et al. A case of paroxysmal nocturnal hemoglobinuria caused by a germline mutation and a somatic mutation in PIGT. Blood. 2013 122(7): 1312-5. doi: 10.1182/blood-2013-01-481499 - Hosokawa K, Kajigaya S, Keyvanfar K, Qiao W, Xie Y, Biancotto A et al. Whole transcriptome sequencing identifies increased CXCR2 expression in PNH granulocytes. Br J Haematol. 2017 177(1): 136-141. doi: 10.1111/bjh.14502 - Oortwijn BD, Roos A, Royle L, van Gijlswijk-Janssen DJ, Faber-Krol MC, Eijgenraam JW et al. Differential glycosylation of polymeric and monomeric IgA: a possible role in glomerular inflammation in IgA nephropathy. J Am Soc Nephrol. 2006 17(12): 3529-39. doi: 10.1681/ASN.2006040388 - Ware RE, Rosse WF, Howard TA. Mutations within the Piga gene in patients with paroxysmal nocturnal hemoglobinuria. Blood. 1994 83(9): 2418-22 - Teye EK, Lu S, Chen F, Yang W, Abraham T, Stairs DB et al. PIGN spatiotemporally regulates the spindle assembly checkpoint proteins in leukemia transformation and progression. Sci Rep. 2021 11(1): 19022. doi: 10.1038/s41598-021-98218-y - Odame Anto E, Owiredu WKBA, Sakyi SA, Turpin CA, Ephraim RKD, Fondjo LA et al. Adverse pregnancy outcomes and imbalance in angiogenic growth mediators and oxidative stress biomarkers is associated with advanced maternal age births: A prospective cohort study in Ghana. PLoS One. 2018 13(7): e0200581. doi: 10.1371/journal.pone.0200581 - Dingli D, Luzzatto L, Pacheco JM. Neutral evolution in paroxysmal nocturnal hemoglobinuria. Proc Natl Acad Sci U S A. 2008 105(47): 18496-500. doi: 10.1073/pnas.0802749105 - Wei XL, Luo TQ, Li JN, Xue ZC, Wang Y, Zhang Y et al. Development and Validation of a Prognostic Classifier Based on Lipid Metabolism-Related Genes in Gastric Cancer. Front Mol Biosci. 2021 8: 691143. doi: 10.3389/fmolb.2021.691143 - Gavriilaki E, Yuan X, Ye Z, Ambinder AJ, Shanbhag SP, Streiff MB et al. Modified Ham test for atypical hemolytic uremic syndrome. Blood. 2015 125(23): 3637-46. doi: 10.1182/blood-2015-02-629683 - Rath G, Aggarwal R, Jawanjal P, Tripathi R, Batra A. HIF-1 Alpha and Placental Growth Factor in Pregnancies Complicated With Preeclampsia: A Qualitative and Quantitative Analysis. J Clin Lab Anal. 2016 30(1): 75-83. doi: 10.1002/jcla.21819 - Ngene NC, Moodley J, Naicker T. The performance of pre-delivery serum concentrations of angiogenic factors in predicting postpartum antihypertensive drug therapy following abdominal delivery in severe preeclampsia and normotensive pregnancy. PLoS One. 2019 14(4): e0215807. doi: 10.1371/journal.pone.0215807 - Perdigones N, Morales M, Mason P, Bessler M. Case Report: Paroxysmal nocturnal hemoglobinuria in a woman heterozygous for G6PD A-. F1000Res. 2014 3: 194. doi: 10.12688/f1000research.4980.2 - Zhou X, Lu Y, Guo P, Zhou C. Upregulation of microRNA‑140‑3p mediates dachshund family transcription factor 1 expression in immunoglobulin A nephropathy through cell cycle‑dependent mechanisms. Mol Med Rep. 2021 23(2): 134. doi: 10.3892/mmr.2020.11773 - Amet T, Lan J, Shepherd N, Yang K, Byrd D, Xing Y et al. Glycosylphosphatidylinositol Anchor Deficiency Attenuates the Production of Infectious HIV-1 and Renders Virions Sensitive to Complement Attack. AIDS Res Hum Retroviruses. 2016 32(10-11): 1100-1112. doi: 10.1089/AID.2016.0046 - Jiang WW, Zahurak M, Zhou ZT, Park HL, Guo ZM, Wu GJ et al. Alterations of GPI transamidase subunits in head and neck squamous carcinoma. Mol Cancer. 2007 6: 74. doi: 10.1186/1476-4598-6-74 - Moehler TM, Seckinger A, Hose D, Andrulis M, Moreaux J, Hielscher T et al. The glycome of normal and malignant plasma cells. PLoS One. 2013 8(12): e83719. doi: 10.1371/journal.pone.0083719 - Santagostino A, Lombardi L, Dine G, Hirsch P, Misra SC. Paroxysmal Nocturnal Hemoglobinuria with a Distinct Molecular Signature Diagnosed Ten Years after Allogenic Bone Marrow Transplantation for Acute Myeloid Leukemia. Case Rep Hematol. 2019 2019: 8928623. doi: 10.1155/2019/8928623 - Brothers KM, Stella NA, Romanowski EG, Kowalski RP, Shanks RM. EepR Mediates Secreted-Protein Production, Desiccation Survival, and Proliferation in a Corneal Infection Model. Infect Immun. 2015 83(11): 4373-82. doi: 10.1128/IAI.00466-15 - Chen R, Eshleman JR, Brodsky RA, Medof ME. Glycophosphatidylinositol-anchored protein deficiency as a marker of mutator phenotypes in cancer. Cancer Res. 2001 61(2): 654-8 - Corat MA, Schlums H, Wu C, Theorell J, Espinoza DA, Sellers SE et al. Acquired somatic mutations in PNH reveal long-term maintenance of adaptive NK cells independent of HSPCs. Blood. 2017 129(14): 1940-1946. doi: 10.1182/blood-2016-08-734285 - Griscelli-Bennaceur A, Gluckman E, Scrobohaci ML, Jonveaux P, Vu T, Bazarbachi A et al. Aplastic anemia and paroxysmal nocturnal hemoglobinuria: search for a pathogenetic link. Blood. 1995 85(5): 1354-63 - Angelova PR, Barilani M, Lovejoy C, Dossena M, Viganò M, Seresini A et al. Mitochondrial dysfunction in Parkinsonian mesenchymal stem cells impairs differentiation. Redox Biol. 2018 14: 474-484. doi: 10.1016/j.redox.2017.10.016 - Brodsky RA, Mukhina GL, Li S, Nelson KL, Chiurazzi PL, Buckley JT et al. Improved detection and characterization of paroxysmal nocturnal hemoglobinuria using fluorescent aerolysin. Am J Clin Pathol. 2000 114(3): 459-66. doi: 10.1093/ajcp/114.3.459 - Savage WJ, Barber JP, Mukhina GL, Hu R, Chen G, Matsui W et al. Glycosylphosphatidylinositol-anchored protein deficiency confers resistance to apoptosis in PNH. Exp Hematol. 2009 37(1): 42-51. doi: 10.1016/j.exphem.2008.09.002 - Taylor VC, Sims M, Brett S, Field MC. Antibody selection against CD52 produces a paroxysmal nocturnal haemoglobinuria phenotype in human lymphocytes by a novel mechanism. Biochem J. 1997 322 ( Pt 3)(Pt 3): 919-25. doi: 10.1042/bj3220919 - Shen W, Clemente MJ, Hosono N, Yoshida K, Przychodzen B, Yoshizato T et al. Deep sequencing reveals stepwise mutation acquisition in paroxysmal nocturnal hemoglobinuria. J Clin Invest. 2014 124(10): 4529-38. doi: 10.1172/JCI74747 - Kalsi J, Delacroix DL, Hodgson HJ. IgA in alcoholic cirrhosis. Clin Exp Immunol. 1983 52(3): 499-504 - Gargiulo L, Papaioannou M, Sica M, Talini G, Chaidos A, Richichi B et al. Glycosylphosphatidylinositol-specific, CD1d-restricted T cells in paroxysmal nocturnal hemoglobinuria. Blood. 2013 121(14): 2753-61. doi: 10.1182/blood-2012-11-469353 - Ware RE, Nishimura J, Moody MA, Smith C, Rosse WF, Howard TA. The PIG-A mutation and absence of glycosylphosphatidylinositol-linked proteins do not confer resistance to apoptosis in paroxysmal nocturnal hemoglobinuria. Blood. 1998 92(7): 2541-50 - Chen R, Nagarajan S, Prince GM, Maheshwari U, Terstappen LW, Kaplan DR et al. Impaired growth and elevated fas receptor expression in PIGA(+) stem cells in primary paroxysmal nocturnal hemoglobinuria. J Clin Invest. 2000 106(5): 689-96. doi: 10.1172/JCI8328 - Nishimura Ji, Phillips KL, Ware RE, Hall S, Wilson L, Gentry TL et al. Efficient retrovirus-mediated PIG-A gene transfer and stable restoration of GPI-anchored protein expression in cells with the PNH phenotype. Blood. 2001 97(10): 3004-10. doi: 10.1182/blood.v97.10.3004 - Bessler M, Mason PJ, Hillmen P, Miyata T, Yamada N, Takeda J et al. Paroxysmal nocturnal haemoglobinuria (PNH) is caused by somatic mutations in the PIG-A gene. EMBO J. 1994 13(1): 110-7 - Sloand EM, Mainwaring L, Keyvanfar K, Chen J, Maciejewski J, Klein HG et al. Transfer of glycosylphosphatidylinositol-anchored proteins to deficient cells after erythrocyte transfusion in paroxysmal nocturnal hemoglobinuria. Blood. 2004 104(12): 3782-8. doi: 10.1182/blood-2004-02-0645 - Liang Y, Zhang J, Zhou Y, Xing G, Zhao G, Liu Z. Proliferation and Cytokine Production of Human Mesangial Cells Stimulated by Secretory IgA Isolated from Patients with IgA Nephropathy. Cell Physiol Biochem. 36(5): 1793-1808. doi: 10.1159/000430151 - Luzzatto L. PNH from mutations of another PIG gene. Blood. 2013 122(7): 1099-100. doi: 10.1182/blood-2013-06-508556 - Yoon JH, Cho HI, Park SS, Chang YH, Kim BK. Mutation analysis of the PIG-A gene in Korean patients with paroxysmal nocturnal haemoglobinuria. J Clin Pathol. 2002 55(6): 410-3. doi: 10.1136/jcp.55.6.410 - Sloand EM, Pfannes L, Scheinberg P, More K, Wu CO, Horne M et al. Increased soluble urokinase plasminogen activator receptor (suPAR) is associated with thrombosis and inhibition of plasmin generation in paroxysmal nocturnal hemoglobinuria (PNH) patients. Exp Hematol. 2008 36(12): 1616-24. doi: 10.1016/j.exphem.2008.06.016 - Schultz IJ, Kiemeney LA, Witjes JA, Schalken JA, Willems JL, Swinkels DW et al. CDC91L1 (PIG-U) mRNA expression in urothelial cell carcinomas. Int J Cancer. 2005 116(2): 282-4. doi: 10.1002/ijc.21040 - Budak MŞ, Toprak G, Akgöl S, Obut M, Oglak C, Baglı I et al. An investigation of the effect of placental growth factor on intrapartum fetal compromise prediction in terminduced high risk pregnancies. Ginekol Pol. 89(12): 700-704. doi: 10.5603/GP.a2018.0118 - Hatanaka M, Seya T, Matsumoto M, Hara T, Nonaka M, Inoue N et al. Mechanisms by which the surface expression of the glycosyl-phosphatidylinositol-anchored complement regulatory proteins decay-accelerating factor (CD55) and CD59 is lost in human leukaemia cell lines. Biochem J. 1996 314 ( Pt 3)(Pt 3): 969-76. doi: 10.1042/bj3140969 - Buck KS, Foster EM, Watson D, Barratt J, Pawluczyk IZ, Knight JF, Feehally J, Allen AC. Expression of T cell receptor variable region families by bone marrow gammadelta T cells in patients with IgA nephropathy. Clin Exp Immunol. 2002 127(3): 527-32. doi: 10.1111/j.1365-2249.2002.01784.x - Peterson LA, Ignatovich IV, Grill AE, Beauchamp A, Ho YY, DiLernia AS, Zhang L. Individual Differences in the Response of Human β-Lymphoblastoid Cells to the Cytotoxic, Mutagenic, and DNA-Damaging Effects of a DNA Methylating Agent, N-Methylnitrosourethane. Chem Res Toxicol. 2019 32(11): 2214-2226. doi: 10.1021/acs.chemrestox.9b00266 - Kerber M, Reiss Y, Wickersheim A, Jugold M, Kiessling F, Heil M et al. Flt-1 signaling in macrophages promotes glioma growth in vivo. Cancer Res. 2008 68(18): 7342-51. doi: 10.1158/0008-5472.CAN-07-6241 - Schena FP, Scivittaro V, Ranieri E, Sinico R, Benuzzi S, Di Cillo M et al. Abnormalities of the IgA immune system in members of unrelated pedigrees from patients with IgA nephropathy. Clin Exp Immunol. 1993 92(1): 139-44. doi: 10.1111/j.1365-2249.1993.tb05960.x - Iida Y, Takeda J, Miyata T, Inoue N, Nishimura J, Kitani T et al. Characterization of genomic PIG-A gene: a gene for glycosylphosphatidylinositol-anchor biosynthesis and paroxysmal nocturnal hemoglobinuria. Blood. 1994 83(11): 3126-31 - Su T, Chapin SJ, Bryant DM, Shewan AM, Young K, Mostov KE. Reduced immunoglobulin A transcytosis associated with immunoglobulin A nephropathy and nasopharyngeal carcinoma. J Biol Chem. 2011 286(52): 44921-5. doi: 10.1074/jbc.M111.296731 - Lee SC, Abdel-Wahab O. The mutational landscape of paroxysmal nocturnal hemoglobinuria revealed: new insights into clonal dominance. J Clin Invest. 2014 124(10): 4227-30. doi: 10.1172/JCI77984 - Sieuwerts AM, Onstenk W, Kraan J, Beaufort CM, Van M, De Laere B et al. AR splice variants in circulating tumor cells of patients with castration-resistant prostate cancer: relation with outcome to cabazitaxel. Mol Oncol. 2019 13(8): 1795-1807. doi: 10.1002/1878-0261.12529 - Robert D, Mahon FX, Richard E, Etienne G, de Verneuil H, Moreau-Gaudry F. A SIN lentiviral vector containing PIGA cDNA allows long-term phenotypic correction of CD34+-derived cells from patients with paroxysmal nocturnal hemoglobinuria. Mol Ther. 2003 7(3): 304-16. doi: 10.1016/s1525-0016(03)00011-x - Ostendorf T, Nischan C, Schubert J, Grussenmeyer T, Scholz C, Zielinska-Skowronek M, Schmidt RE. Heterogeneous PIG-A mutations in different cell lineages in paroxysmal nocturnal hemoglobinuria. Blood. 1995 85(6): 1640-6 - Miyata T, Yamada N, Iida Y, Nishimura J, Takeda J, Kitani T et al. Abnormalities of PIG-A transcripts in granulocytes from patients with paroxysmal nocturnal hemoglobinuria. N Engl J Med. 1994 330(4): 249-55. doi: 10.1056/NEJM199401273300404 - Maciejewski JP, Sloand EM, Sato T, Anderson S, Young NS. Impaired hematopoiesis in paroxysmal nocturnal hemoglobinuria/aplastic anemia is not associated with a selective proliferative defect in the glycosylphosphatidylinositol-anchored protein-deficient clone. Blood. 1997 89(4): 1173-81 - Delacroix DL, Hodgson HJ, McPherson A, Dive C, Vaerman JP. Selective transport of polymeric immunoglobulin A in bile. Quantitative relationships of monomeric and polymeric immunoglobulin A, immunoglobulin M, and other proteins in serum, bile, and saliva. J Clin Invest. 1982 70(2): 230-41. doi: 10.1172/jci110610 - Oved JH, Stanley N, Babushok DV, Huang Y, Duke JL, Monos DS et al. Development of hemolytic paroxysmal nocturnal hemoglobinuria without graft loss following hematopoietic stem cell transplantation for acquired aplastic anemia. Pediatr Transplant. 2019 23(4): e13393. doi: 10.1111/petr.13393 - Layward L, Allen AC, Harper SJ, Hattersley JM, Feehally J. Increased and prolonged production of specific polymeric IgA after systemic immunization with tetanus toxoid in IgA nephropathy. Clin Exp Immunol. 1992 88(3): 394-8. doi: 10.1111/j.1365-2249.1992.tb06460.x - Leung JC, Chan LY, Tang SC, Lam MF, Chow CW, Lim AI et al. Oxidative damages in tubular epithelial cells in IgA nephropathy: role of crosstalk between angiotensin II and aldosterone. J Transl Med. 2011 9: 169. doi: 10.1186/1479-5876-9-169 - Dezern AE, Borowitz MJ. ICCS/ESCCA consensus guidelines to detect GPI-deficient cells in paroxysmal nocturnal hemoglobinuria (PNH) and related disorders part 1 - clinical utility. Cytometry B Clin Cytom. 2018 94(1): 16-22. doi: 10.1002/cyto.b.21608 - Brodsky RA, Mukhina GL, Nelson KL, Lawrence TS, Jones RJ, Buckley JT. Resistance of paroxysmal nocturnal hemoglobinuria cells to the glycosylphosphatidylinositol-binding toxin aerolysin. Blood. 1999 93(5): 1749-56 - Vaerman JP, Langendries A, Giffroy D, Brandtzaeg P, Kobayashi K. Lack of SC/pIgR-mediated epithelial transport of a human polymeric IgA devoid of J chain: in vitro and in vivo studies. Immunology. 1998 95(1): 90-6. doi: 10.1046/j.1365-2567.1998.00560.x - Birkenhäger R, Schneppe B, Röckl W, Wilting J, Weich HA, McCarthy JE. Synthesis and physiological activity of heterodimers comprising different splice forms of vascular endothelial growth factor and placenta growth factor. Biochem J. 1996 316 ( Pt 3)(Pt 3): 703-7. doi: 10.1042/bj3160703 - Monsky WL, Fukumura D, Gohongi T, Ancukiewcz M, Weich HA, Torchilin VP et al. Augmentation of transvascular transport of macromolecules and nanoparticles in tumors using vascular endothelial growth factor. Cancer Res. 1999 59(16): 4129-35 - Takehara E, Mandai S, Shikuma S, Akita W, Chiga M, Mori T et al. Post-infectious Proliferative Glomerulonephritis with Monoclonal Immunoglobulin G Deposits Associated with Complement Factor H Mutation. Intern Med. 2017 56(7): 811-817. doi: 10.2169/internalmedicine.56.7778 - Yamada N, Miyata T, Maeda K, Kitani T, Takeda J, Kinoshita T. Somatic mutations of the PIG-A gene found in Japanese patients with paroxysmal nocturnal hemoglobinuria. Blood. 1995 85(4): 885-92 - Cao Y, Linden P, Shima D, Browne F, Folkman J. In vivo angiogenic activity and hypoxia induction of heterodimers of placenta growth factor/vascular endothelial growth factor. J Clin Invest. 1996 98(11): 2507-11. doi: 10.1172/JCI119069 - Ware RE, Rosse WF, Hall SE. Immunophenotypic analysis of reticulocytes in paroxysmal nocturnal hemoglobinuria. Blood. 1995 86(4): 1586-9 - Lai KN, Chan LY, Tang SC, Tsang AW, Guo H, Tse KC et al. Characteristics of polymeric lambda-IgA binding to leukocytes in IgA nephropathy. J Am Soc Nephrol. 2002 13(9): 2309-19. doi: 10.1097/01.asn.0000026497.82930.73 - Mortazavi Y, Merk B, McIntosh J, Marsh JC, Schrezenmeier H, Rutherford TR et al. The spectrum of PIG-A gene mutations in aplastic anemia/paroxysmal nocturnal hemoglobinuria (AA/PNH): a high incidence of multiple mutations and evidence of a mutational hot spot. Blood. 2003 101(7): 2833-41. doi: 10.1182/blood-2002-07-2095 - Mao Y, Pei N, Chen X, Chen H, Yan R, Bai N et al. Angiotensin 1-7 Overexpression Mediated by a Capsid-optimized AAV8 Vector Leads to Significant Growth Inhibition of Hepatocellular Carcinoma In vivo. Int J Biol Sci. 2018 14(1): 57-68. doi: 10.7150/ijbs.22235 - Brodsky RA, Vala MS, Barber JP, Medof ME, Jones RJ. Resistance to apoptosis caused by PIG-A gene mutations in paroxysmal nocturnal hemoglobinuria. Proc Natl Acad Sci U S A. 1997 94(16): 8756-60. doi: 10.1073/pnas.94.16.8756 - Franco De Carvalho R, Arruda VR, Saad ST, Costa FF. Detection of somatic mutations of the PIG-A gene in Brazilian patients with paroxysmal nocturnal hemoglobinuria. Braz J Med Biol Res. 2001 34(6): 763-6. doi: 10.1590/s0100-879x2001000600010 - Bartholomeusz RC, Forrest BD, Labrooy JT, Ey PL, Pyle D, Shearman DJ, Rowley D. The serum polymeric IgA antibody response to typhoid vaccination; its relationship to the intestinal IgA response. Immunology. 1990 69(2): 190-4 - Cossu A, Posadino AM, Giordo R, Emanueli C, Sanguinetti AM, Piscopo A et al. Apricot melanoidins prevent oxidative endothelial cell death by counteracting mitochondrial oxidation and membrane depolarization. PLoS One. 2012 7(11): e48817. doi: 10.1371/journal.pone.0048817 - Delacroix DL, Elkom KB, Geubel AP, Hodgson HF, Dive C, Vaerman JP. Changes in size, subclass, and metabolic properties of serum immunoglobulin A in liver diseases and in other diseases with high serum immunoglobulin A. J Clin Invest. 1983 71(2): 358-67. doi: 10.1172/jci110777 - Katagiri T, Kawamoto H, Nakakuki T, Ishiyama K, Okada-Hatakeyama M, Ohtake S, Seiki Y et al. Individual hematopoietic stem cells in human bone marrow of patients with aplastic anemia or myelodysplastic syndrome stably give rise to limited cell lineages. Stem Cells. 2013 31(3): 536-46. doi: 10.1002/stem.1301 - Hu R, Dunn TA, Wei S, Isharwal S, Veltri RW, Humphreys E et al. Ligand-independent androgen receptor variants derived from splicing of cryptic exons signify hormone-refractory prostate cancer. Cancer Res. 2009 69(1): 16-22. doi: 10.1158/0008-5472.CAN-08-2764 - Braathen R, Hohman VS, Brandtzaeg P, Johansen FE. Secretory antibody formation: conserved binding interactions between J chain and polymeric Ig receptor from humans and amphibians. J Immunol. 2007 178(3): 1589-97. doi: 10.4049/jimmunol.178.3.1589 - Mannelli F, Bencini S, Peruzzi B, Cutini I, Sanna A, Benelli M et al. A systematic analysis of bone marrow cells by flow cytometry defines a specific phenotypic profile beyond GPI deficiency in paroxysmal nocturnal hemoglobinuria. Cytometry B Clin Cytom. 2013 84(2): 71-81. doi: 10.1002/cyto.b.21064 - Rosti V, Tremml G, Soares V, Pandolfi PP, Luzzatto L, Bessler M. Murine embryonic stem cells without pig-a gene activity are competent for hematopoiesis with the PNH phenotype but not for clonal expansion. J Clin Invest. 1997 100(5): 1028-36. doi: 10.1172/JCI119613 - Nagarajan S, Brodsky RA, Young NS, Medof ME. Genetic defects underlying paroxysmal nocturnal hemoglobinuria that arises out of aplastic anemia. Blood. 1995 86(12): 4656-61 - Stern LL, Mason JB, Selhub J, Choi SW. Genomic DNA hypomethylation, a characteristic of most cancers, is present in peripheral leukocytes of individuals who are homozygous for the C677T polymorphism in the methylenetetrahydrofolate reductase gene. Cancer Epidemiol Biomarkers Prev. 2000 9(8): 849-53 - Rifai A, Millard K. Glomerular deposition of immune complexes prepared with monomeric or polymeric IgA. Clin Exp Immunol. 1985 60(2): 363-8 - Nafa K, Mason PJ, Hillmen P, Luzzatto L, Bessler M. Mutations in the PIG-A gene causing paroxysmal nocturnal hemoglobinuria are mainly of the frameshift type. Blood. 1995 86(12): 4650-5 - Van Etten JL, Nyquist M, Li Y, Yang R, Ho Y, Johnson R et al. Targeting a Single Alternative Polyadenylation Site Coordinately Blocks Expression of Androgen Receptor mRNA Splice Variants in Prostate Cancer. Cancer Res. 2017 77(19): 5228-5235. doi: 10.1158/0008-5472.CAN-17-0320 - Baker DL, Fujiwara Y, Pigg KR, Tsukahara R, Kobayashi S, Murofushi H et al. Carba analogs of cyclic phosphatidic acid are selective inhibitors of autotaxin and cancer cell invasion and metastasis. J Biol Chem. 2006 281(32): 22786-93. doi: 10.1074/jbc.M512486200 - Togashi T, Choi DK, Taylor TD, Suzuki Y, Sugano S, Hattori M et al. A novel gene, DSCR5, from the distal Down syndrome critical region on chromosome 21q22.2. DNA Res. 2000 7(3): 207-12. doi: 10.1093/dnares/7.3.207 - Oba-Yamamoto C, Kameda H, Miyoshi H, Sekizaki T, Takase T, Yanagimachi T et al. Acromegaly Cases Exhibiting Increased Growth Hormone Levels during Oral Glucose Loading with Preadministration of Dipeptidyl Peptidase-4 Inhibitor. Intern Med. 2021 60(15): 2375-2383. doi: 10.2169/internalmedicine.4755-20 - Pramoonjago P, Wanachiwanawin W, Chinprasertsak S, Pattanapanayasat K, Takeda J, Kinoshita T. Somatic mutations of PIG-A in Thai patients with paroxysmal nocturnal hemoglobinuria. Blood. 1995 86(5): 1736-9 - Nishimura J, Inoue N, Wada H, Ueda E, Pramoonjago P, Hirota T et al. A patient with paroxysmal nocturnal hemoglobinuria bearing four independent PIG-A mutant clones. Blood. 1997 89(9): 3470-6 - Endo M, Ware RE, Vreeke TM, Singh SP, Howard TA, Tomita A et al. Molecular basis of the heterogeneity of expression of glycosyl phosphatidylinositol anchored proteins in paroxysmal nocturnal hemoglobinuria. Blood. 1996 87(6): 2546-57 - Endo M, Beatty PG, Vreeke TM, Wittwer CT, Singh SP, Parker CJ. Syngeneic bone marrow transplantation without conditioning in a patient with paroxysmal nocturnal hemoglobinuria: in vivo evidence that the mutant stem cells have a survival advantage. Blood. 1996 88(2): 742-50 - Avnet S, Cenni E, Granchi D, Perut F, Amato I, Battistelli L et al. Isolation and characterization of a new cell line from a renal carcinoma bone metastasis. Anticancer Res. 2004 24(3a): 1705-11 - Cappeller WA, Bloch KJ, Hatz RA, Carter EA, Fagundes J, Sullivan DA et al. Reduction in biliary IgA after burn injury. Role of diminished delivery via the thoracic duct and of enhanced loss from the systemic circulation. Ann Surg. 1992 215(4): 338-43. doi: 10.1097/00000658-199204000-00006 - Kageyama Y, Miwa H, Tawara I, Ohishi K, Masuya M, Katayama N. A population of CD20+CD27+CD43+CD38lo/int B1 cells in PNH are missing GPI-anchored proteins and harbor PIGA mutations. Blood. 2019 134(1): 89-92. doi: 10.1182/blood.2019001343 - Barillari G, Albonici L, Franzese O, Modesti A, Liberati F, Barillari P et al. The basic residues of placenta growth factor type 2 retrieve sequestered angiogenic factors into a soluble form: implications for tumor angiogenesis. Am J Pathol. 1998 152(5): 1161-6 - Soragni E, Miao W, Iudicello M, Jacoby D, De Mercanti S, Clerico M et al. Epigenetic therapy for Friedreich ataxia. Ann Neurol. 2014 76(4): 489-508. doi: 10.1002/ana.24260 - Lee JE, Kim C, Yang H, Park I, Oh N, Hua S et al. Novel glycosylated VEGF decoy receptor fusion protein, VEGF-Grab, efficiently suppresses tumor angiogenesis and progression. Mol Cancer Ther. 2015 14(2): 470-9. doi: 10.1158/1535-7163.MCT-14-0968-T - Pronicka E, Piekutowska-Abramczuk D, Ciara E, Trubicka J, Rokicki D, Karkucińska-Więckowska A et al. New perspective in diagnostics of mitochondrial disorders: two years' experience with whole-exome sequencing at a national paediatric centre. J Transl Med. 2016 14(1): 174. doi: 10.1186/s12967-016-0930-9 - Nagakura S, Ishihara S, Dunn DE, Nishimura J, Kawaguchi T, Horikawa K et al. Decreased susceptibility of leukemic cells with PIG-A mutation to natural killer cells in vitro. Blood. 2002 100(3): 1031-7. doi: 10.1182/blood.v100.3.1031 - Cochery-Nouvellon É, Mercier É, Bouvier S, Balducchi JP, Quéré I, Perez-Martin A et al. Obstetric antiphospholipid syndrome: early variations of angiogenic factors are associated with adverse outcomes. Haematologica. 2017 102(5): 835-842. doi: 10.3324/haematol.2016 - Chen Y, Tao S, Deng Y, Song L, Yu L. Chronic myeloid leukemia transformation in a patient with paroxysmal nocturnal hemoglobinuria: a rare case report with literature review. Int J Clin Exp Med. 2015 8(5): 8226-9 - Miyagami S, Koide K, Sekizawa A, Ventura W, Yotsumoto J, Oishi S et al. Physiological changes in the pattern of placental gene expression early in the first trimester. Reprod Sci. 2013 20(6): 710-4. doi: 10.1177/1933719112466309 - Pu JJ, Hu R, Mukhina GL, Carraway HE, McDevitt MA, Brodsky RA. The small population of PIG-A mutant cells in myelodysplastic syndromes do not arise from multipotent hematopoietic stem cells. Haematologica. 2012 97(8): 1225-33. doi: 10.3324/haematol.2011.048215 - Serra M, Longo F, Roetto A, Sandri A, Piga A. A child with hyperferritinemia: case report. Ital J Pediatr. 2011 37: 20. doi: 10.1186/1824-7288-37-20 - Zhang L, Qi H, Liu Z, Peng WJ, Cao H, Guo CY et al. Construction of a ceRNA coregulatory network and screening of hub biomarkers for salt-sensitive hypertension. J Cell Mol Med. 2020 24(13): 7254-7265. doi: 10.1111/jcmm.15285 - Kai T, Shichishima T, Noji H, Yamamoto T, Okamoto M, Ikeda K et al. Phenotypes and phosphatidylinositol glycan-class A gene abnormalities during cell differentiation and maturation from precursor cells to mature granulocytes in patients with paroxysmal nocturnal hemoglobinuria. Blood. 2002 100(10): 3812-8. doi: 10.1182/blood.V100.10.3812 - Li L, Fridley BL, Kalari K, Niu N, Jenkins G, Batzler A et al. Discovery of genetic biomarkers contributing to variation in drug response of cytidine analogues using human lymphoblastoid cell lines. BMC Genomics. 2014 15: 93. doi: 10.1186/1471-2164-15-93 - Parente YDM, Castro AL, Araújo FB, Teixeira AC, Lima ÍC, Daher EF. Acute renal failure by rapidly progressive glomerulonephritis with IgA deposition in a patient concomitantly diagnosed with multibacillary Hansen's disease: a case report. J Bras Nefrol. 2019 41(1): 152-156. doi: 10.1590/2175-8239-jbn-2018-0056 - In JG, Tuma PL. MAL2 selectively regulates polymeric IgA receptor delivery from the Golgi to the plasma membrane in WIF-B cells. Traffic. 2010 11(8): 1056-66. doi: 10.1111/j.1600-0854.2010.01074.x - Keith WN, Vulliamy T, Zhao J, Ar C, Erzik C, Bilsland A et al. A mutation in a functional Sp1 binding site of the telomerase RNA gene (hTERC) promoter in a patient with Paroxysmal Nocturnal Haemoglobinuria. BMC Blood Disord. 2004 4(1): 3. doi: 10.1186/1471-2326-4-3 - Aydinok Y, Porter JB, Piga A, Elalfy M, El-Beshlawy A, Kilinç Y et al. Prevalence and distribution of iron overload in patients with transfusion-dependent anemias differs across geographic regions: results from the CORDELIA study. Eur J Haematol. 2015 95(3): 244-53. doi: 10.1111/ejh.12487 - Knuuttila M, Yatkin E, Kallio J, Savolainen S, Laajala TD, Aittokallio T et al. Castration induces up-regulation of intratumoral androgen biosynthesis and androgen receptor expression in an orthotopic VCaP human prostate cancer xenograft model. Am J Pathol. 2014 184(8): 2163-73. doi: 10.1016/j.ajpath.2014.04.010 - Cao F, Souders Ii CL, Perez-Rodriguez V, Martyniuk CJ. Elucidating Conserved Transcriptional Networks Underlying Pesticide Exposure and Parkinson's Disease: A Focus on Chemicals of Epidemiological Relevance. Front Genet. 2019 9: 701. doi: 10.3389/fgene.2018.00701 - Reid ES, Papandreou A, Drury S, Boustred C, Yue WW, Wedatilake Y et al. Advantages and pitfalls of an extended gene panel for investigating complex neurometabolic phenotypes. Brain. 2016 139(11): 2844-2854. doi: 10.1093/brain/aww221 - Deuchler S, Schubert R, Singh P, Chedid A, Brui N, Kenikstul N et al. Vitreous expression of cytokines and growth factors in patients with diabetic retinopathy-An investigation of their expression based on clinical diabetic retinopathy grade. PLoS One. 2021 16(5): e0248439. doi: 10.1371/journal.pone.0248439 - Weizhong Z, Shuohui G, Hanjiao Q, Yuhong M, Xiaohua Y, Jian C et al. Inhibition of cytohesin-1 by siRNA leads to reduced IGFR signaling in prostate cancer. Braz J Med Biol Res. 2011 44(7): 642-6. doi: 10.1590/s0100-879x2011007500072 - Hosokawa K, Kajigaya S, Keyvanfar K, Qiao W, Xie Y, Townsley DM et al. T Cell Transcriptomes from Paroxysmal Nocturnal Hemoglobinuria Patients Reveal Novel Signaling Pathways. J Immunol. 2017 199(2): 477-488. doi: 10.4049/jimmunol.1601299 - raulsen A, Pacheco JM, Dingli D. On the origin of multiple mutant clones in paroxysmal nocturnal hemoglobinuria. Stem Cells. 2007 25(12): 3081-4. doi: 10.1634/stemcells.2007-0427 - Perez-Ilzarbe M, Agbulut O, Pelacho B, Ciorba C, San Jose-Eneriz E, Desnos M et al. Characterization of the paracrine effects of human skeletal myoblasts transplanted in infarcted myocardium. Eur J Heart Fail. 2008 10(11): 1065-72. doi: 10.1016/j.ejheart.2008.08.002 - Schellevis RL, Breukink MB, Gilissen C, Boon CJF, Hoyng CB, de Jong EK et al. Exome sequencing in patients with chronic central serous chorioretinopathy. Sci Rep. 2019 9(1): 6598. doi: 10.1038/s41598-019-43152-3 - Lynch AM, Murphy JR, Gibbs RS, Levine RJ, Giclas PC, Salmon JE et al. The interrelationship of complement-activation fragments and angiogenesis-related factors in early pregnancy and their association with pre-eclampsia. BJOG. 2010 117(4): 456-62. doi: 10.1111/j.1471-0528.2009.02473.x - Bhalli JA, Shaddock JG, Pearce MG, Dobrovolsky VN. Sensitivity of the Pig-a assay for detecting gene mutation in rats exposed acutely to strong clastogens. Mutagenesis. 2013 28(4): 447-55. doi: 10.1093/mutage/get022 - Reljič M, Porović A. Maternal serum levels of angiogenic markers and markers of placentation in pregnancies conceived with fresh and vitrified-warmed blastocyst transfer. J Assist Reprod Genet. 2019 36(7): 1489-1495. doi: 10.1007/s10815-019-01484-z - Robak E, Kulczycka-Siennicka L, Gerlicz Z, Kierstan M, Korycka-Wolowiec A, Sysa-Jedrzejowska A. Correlations between concentrations of interleukin (IL)-17A, IL-17B and IL-17F, and endothelial cells and proangiogenic cytokines in systemic lupus erythematosus patients. Eur Cytokine Netw. 2013 24(1): 60-8. doi: 10.1684/ecn.2013.0330 - Colombel JF, Vaerman JP, Hällgren R, Dehennin JP, Wain E, Modigliani R, Cortot A. Effect of intrajejunal elemental diet perfusion on jejunal secretion of immunoglobulins, albumin, and hyaluronan in man. Gut. 1992 33(1): 44-7. doi: 10.1136/gut.33.1.44 - Piga A. Impact of bone disease and pain in thalassemia. Hematology Am Soc Hematol Educ Program. 2017 2017(1): 272-277. doi: 10.1182/asheducation-2017.1.272 - Mathew P, Wen S, Morita S, Thall PF. Placental growth factor and soluble c-kit receptor dynamics characterize the cytokine signature of imatinib in prostate cancer and bone metastases. J Interferon Cytokine Res. 2011 31(7): 539-44. doi: 10.1089/jir.2010.0142 - Stefanaki I, Panagiotou OA, Kodela E, Gogas H, Kypreou KP, Chatzinasiou F et al. Replication and predictive value of SNPs associated with melanoma and pigmentation traits in a Southern European case-control study. PLoS One. 2013 8(2): e55712. doi: 10.1371/journal.pone.0055712 - Martin TM, Plautz SA, Pannier AK. Network analysis of endogenous gene expression profiles after polyethyleneimine-mediated DNA delivery. J Gene Med. 2013 15(3-4): 142-54. doi: 10.1002/jgm.2704 - Fasching CE, Grossman T, Corthésy B, Plaut AG, Weiser JN, Janoff EN. Impact of the molecular form of immunoglobulin A on functional activity in defense against Streptococcus pneumoniae. Infect Immun. 2007 75(4): 1801-10. doi: 10.1128/IAI.01758-06 - Gristwood T, Fineran PC, Everson L, Williamson NR, Salmond GP. The PhoBR two-component system regulates antibiotic biosynthesis in Serratia in response to phosphate. BMC Microbiol. 2009 9: 112. doi: 10.1186/1471-2180-9-112 - Piga I, Verza M, Montenegro F, Nardo G, Zulato E, Zanin T et al. In situ Metabolic Profiling of Ovarian Cancer Tumor Xenografts: A Digital Pathology Approach. Front Oncol. 2020 10: 1277. doi: 10.3389/fonc.2020.01277 - Yuan X, Gavriilaki E, Thanassi JA, Yang G, Baines AC, Podos SD et al. Small-molecule factor D inhibitors selectively block the alternative pathway of complement in paroxysmal nocturnal hemoglobinuria and atypical hemolytic uremic syndrome. Haematologica. 2017 102(3): 466-475. doi: 10.3324/haematol.2016.153312 - Di Antonio L, Toto L, Mastropasqua A, Brescia L, Erroi E, Lamolinara A et al. Retinal vascular changes and aqueous humor cytokines changes after aflibercept intravitreal injection in treatment-naïve myopic choroidal neovascularization. Sci Rep. 2018 8(1): 15631. doi: 10.1038/s41598-018-33926-6 - Torry RJ, Tomanek RJ, Zheng W, Miller SJ, Labarrere CA, Torry DS. Hypoxia increases placenta growth factor expression in human myocardium and cultured neonatal rat cardiomyocytes. J Heart Lung Transplant. 2009 28(2): 183-90. doi: 10.1016/j.healun.2008 - Mochizuki K, Sugimori C, Qi Z, Lu X, Takami A, Ishiyama K et al. Expansion of donor-derived hematopoietic stem cells with PIGA mutation associated with late graft failure after allogeneic stem cell transplantation. Blood. 2008 112(5): 2160-2. doi: 10.1182/blood-2008-02-141325 - Shi Y, Zhuang Y, Zhang J, Chen M, Wu S. Identification of Tumorigenic and Prognostic Biomarkers in Colorectal Cancer Based on microRNA Expression Profiles. Biomed Res Int. 2020 2020: 7136049. doi: 10.1155/2020/7136049 - Ortega MA, Saez MA, Fraile-Martínez O, Asúnsolo Á, Pekarek L, Bravo C et al. Increased Angiogenesis and Lymphangiogenesis in the Placental Villi of Women with Chronic Venous Disease during Pregnancy. Int J Mol Sci. 2020 21(7): 2487. doi: 10.3390/ijms21072487 - Jayasinghe C, Simiantonaki N, Kirkpatrick CJ. Cell type- and tumor zone-specific expression of pVEGFR-1 and its ligands influence colon cancer metastasis. BMC Cancer. 2015 15: 104. doi: 10.1186/s12885-015-1130-3 - Pei N, Wan R, Chen X, Li A, Zhang Y, Li J et al. Angiotensin-(1-7) Decreases Cell Growth and Angiogenesis of Human Nasopharyngeal Carcinoma Xenografts. Mol Cancer Ther. 2016 15(1): 37-47. doi: 10.1158/1535-7163.MCT-14-0981 - Wang Z, Chakravarty G, Kim S, Yazici YD, Younes MN, Jasser SA et al. Growth-inhibitory effects of human anti-insulin-like growth factor-I receptor antibody (A12) in an orthotopic nude mouse model of anaplastic thyroid carcinoma. Clin Cancer Res. 2006 12(15): 4755-65. doi: 10.1158/1078-0432.CCR-05-2691 - Grebe T, Paik J, Hakenbeck R. A novel resistance mechanism against beta-lactams in Streptococcus pneumoniae involves CpoA, a putative glycosyltransferase. J Bacteriol. 1997 179(10): 3342-9. doi: 10.1128/jb.179.10.3342-3349.1997 - Rondelli T, Berardi M, Peruzzi B, Boni L, Caporale R, Dolara P et al. The frequency of granulocytes with spontaneous somatic mutations: a wide distribution in a normal human population. PLoS One. 2013 8(1): e54046. doi: 10.1371/journal.pone.0054046 - Inoue N, Izui-Sarumaru T, Murakami Y, Endo Y, Nishimura J, Kurokawa K et al. Molecular basis of clonal expansion of hematopoiesis in 2 patients with paroxysmal nocturnal hemoglobinuria (PNH). Blood. 2006 108(13): 4232-6. doi: 10.1182/blood-2006-05-025148 - Lai KN, Chan LY, Tang SC, Tsang AW, Li FF, Lam MF et al. Mesangial expression of angiotensin II receptor in IgA nephropathy and its regulation by polymeric IgA1. Kidney Int. 2004 66(4): 1403-16. doi: 10.1111/j.1523-1755.2004.00874.x - Young NS, Maciejewski JP. Genetic and environmental effects in paroxysmal nocturnal hemoglobinuria: this little PIG-A goes "Why? Why? Why?". J Clin Invest. 2000 106(5): 637-41. doi: 10.1172/JCI11002 - Yamashita T, Jinnin M, Makino K, Kajihara I, Aoi J, Masuguchi S et al. Serum cytokine profiles are altered in patients with progressive infantile hemangioma. Biosci Trends. 2018 12(4): 438-441. doi: 10.5582/bst.2018.01118 - Mongrain V, Hernandez SA, Pradervand S, Dorsaz S, Curie T, Hagiwara G et al. Separating the contribution of glucocorticoids and wakefulness to the molecular and electrophysiological correlates of sleep homeostasis. Sleep. 2010 33(9): 1147-57. doi: 10.1093/sleep/33.9.1147 - Bolasco P, Serra A, Loi M, Galfré A, Piga M. Failed Switching off in the MIBI-Parathyroid Scintigraphy in a Dialyzed Patient with Secondary Hyperparathyroidism Responsive to Cinacalcet Therapy. Int J Endocrinol. 2010 2010: 206801. doi: 10.1155/2010/206801 - Hillebrand AC, Pizzolato LS, Neto BS, Branchini G, Brum IS. Androgen receptor isoforms expression in benign prostatic hyperplasia and primary prostate cancer. PLoS One. 2018 13(7): e0200613. doi: 10.1371/journal.pone.0200613 - Haboubi HN, Lawrence RL, Rees B, Williams L, Manson JM, Al-Mossawi N et al. Developing a blood-based gene mutation assay as a novel biomarker for oesophageal adenocarcinoma. Sci Rep. 2019 9(1): 5168. doi: 10.1038/s41598-019-41490-w - Mestan KK, Gotteiner N, Porta N, Grobman W, Su EJ, Ernst LM. Cord Blood Biomarkers of Placental Maternal Vascular Underperfusion Predict Bronchopulmonary Dysplasia-Associated Pulmonary Hypertension. J Pediatr. 2017 185: 33-41. doi: 10.1016/j.jpeds.2017.01.015 - Long AS, Wills JW, Krolak D, Guo M, Dertinger SD, Arlt VM et al. Benchmark dose analyses of multiple genetic toxicity endpoints permit robust, cross-tissue comparisons of MutaMouse responses to orally delivered benzo[a]pyrene. Arch Toxicol. 2018 92(2): 967-982. doi: 10.1007/s00204-017-2099-2 - Hörnberg E, Ylitalo EB, Crnalic S, Antti H, Stattin P, Widmark A et al. Expression of androgen receptor splice variants in prostate cancer bone metastases is associated with castration-resistance and short survival. PLoS One. 2011 6(4): e19059. doi: 10.1371/journal.pone.0019059 - Rechoum Y, Rovito D, Iacopetta D, Barone I, Andò S, Weigel NL et al. AR collaborates with ERα in aromatase inhibitor-resistant breast cancer. Breast Cancer Res Treat. 2014 147(3): 473-85. doi: 10.1007/s10549-014-3082-8 - Cheng Y, Zheng S, Pan CT, Yuan M, Chang L, Yao Y et al. Analysis of aqueous humor concentrations of cytokines in retinoblastoma. PLoS One. 2017 12(5): e0177337. doi: 10.1371/journal.pone.0177337 - Gobble RM, Groesch KA, Chang M, Torry RJ, Torry DS. Differential regulation of human PlGF gene expression in trophoblast and nontrophoblast cells by oxygen tension. Placenta. 2009 30(10): 869-75. doi: 10.1016/j.placenta.2009.08.003 - Li L, Liu H, Wang H, Liu Z, Chen Y, Li L et al. Abnormal expression and mutation of the RBPJ gene may be involved in CD59- clonal proliferation in paroxysmal nocturnal hemoglobinuria. Exp Ther Med. 2019 17(6): 4536-4546. doi: 10.3892/etm.2019.7475 - Nevo Y, Ben-Zeev B, Tabib A, Straussberg R, Anikster Y, Shorer Z et al. CD59 deficiency is associated with chronic hemolysis and childhood relapsing immune-mediated polyneuropathy. Blood. 2013 121(1): 129-35. doi: 10.1182/blood-2012-07-441857 - Mon Père N, Lenaerts T, Pacheco JM, Dingli D. Evolutionary dynamics of paroxysmal nocturnal hemoglobinuria. PLoS Comput Biol. 2018 14(6): e1006133. doi: 10.1371/journal.pcbi.1006133 - Gąsiorowska A, Pietryga M, Zawiejska A, Dydowicz P, Ziółkowska K, Wolski H et al. Chorionic thickness and PlGF concentrations as early predictors of small-for-gestational age birth weight in a low risk population. Ginekol Pol. 2017 88(2): 87-95 - Podkowinski D, Orlowski-Wimmer E, Zlabinger G, Pollreisz A, Mursch-Edlmayr AS, Mariacher S et al. Aqueous humour cytokine changes during a loading phase of intravitreal ranibizumab or dexamethasone implant in diabetic macular oedema. Acta Ophthalmol. 2020 98(4): e407-e415. doi: 10.1111/aos.14297 - Gao Y, Li Y, Niu X, Wu Y, Guan X, Hong Y et al. Identification and Validation of Prognostically Relevant Gene Signature in Melanoma. Biomed Res Int. 2020 2020: 5323614. doi: 10.1155/2020/5323614 - Hertenstein B, Wagner B, Bunjes D, Duncker C, Raghavachar A, Arnold R et al. Emergence of CD52-, phosphatidylinositolglycan-anchor-deficient T lymphocytes after in vivo application of Campath-1H for refractory B-cell non-Hodgkin lymphoma. Blood. 1995 86(4): 1487-92 - Mansouri S, Suppiah S, Mamatjan Y, Paganini I, Liu JC, Karimi S et al. Epigenomic, genomic, and transcriptomic landscape of schwannomatosis. Acta Neuropathol. 2021 141(1): 101-116. doi: 10.1007/s00401-020-02230-x - Dias-Junior CA, Chen J, Cui N, Chiang CL, Zhu M, Ren Z et al. Angiogenic imbalance and diminished matrix metalloproteinase-2 and -9 underlie regional decreases in uteroplacental vascularization and feto-placental growth in hypertensive pregnancy. Biochem Pharmacol. 2017 146: 101-116. doi: 10.1016/j.bcp.2017.09.005 - Yue Y, Jiang H, Liu R, Yin Y, Zhang Y, Liang J et al. Towards a multi protein and mRNA expression of biological predictive and distinguish model for post stroke depression. Oncotarget. 2016 7(34): 54329-54338. doi: 10.18632/oncotarget.11105 - Wang HF, Zheng SF, Chen Y, Zhou ZY, Xu J. Correlations between claudin-1 and PIGF expressions in retinoblastoma. Eur Rev Med Pharmacol Sci. 2018 22(13): 4196-4203. doi: 10.26355/eurrev_201807_15413 - Brzóska K, Kruszewski M. Toward the development of transcriptional biodosimetry for the identification of irradiated individuals and assessment of absorbed radiation dose. Radiat Environ Biophys. 2015 54(3): 353-63. doi: 10.1007/s00411-015-0603-8 - Hebbar P, Nizam R, Melhem M, Alkayal F, Elkum N, John SE et al. Genome-wide association study identifies novel recessive genetic variants for high TGs in an Arab population. J Lipid Res. 2018 59(10): 1951-1966. doi: 10.1194/jlr.P080218 - Kauraniemi P, Kuukasjärvi T, Sauter G, Kallioniemi A. Amplification of a 280-kilobase core region at the ERBB2 locus leads to activation of two hypothetical proteins in breast cancer. Am J Pathol. 2003 163(5): 1979-84. doi: 10.1016/S0002-9440(10)63556-0 - Bruno A, Focaccetti C, Pagani A, Imperatori AS, Spagnoletti M, Rotolo N et al. The proangiogenic phenotype of natural killer cells in patients with non-small cell lung cancer. Neoplasia. 2013 15(2): 133-42. doi: 10.1593/neo.121758 - Dalrymple A, Ordoñez P, Thorne D, Walker D, Camacho OM, Büttner A et al. Cigarette smoke induced genotoxicity and respiratory tract pathology: evidence to support reduced exposure time and animal numbers in tobacco product testing. Inhal Toxicol. 2016 28(7): 324-38. doi: 10.3109/08958378.2016.1170911 - Calvo M, Pol A, Lu A, Ortega D, Pons M, Blasi J et al. Cellubrevin is present in the basolateral endocytic compartment of hepatocytes and follows the transcytotic pathway after IgA internalization. J Biol Chem. 2000 275(11): 7910-7. doi: 10.1074/jbc.275.11.7910 - Yang X, Liu Q, Zou J, Li YK, Xie X. Identification of a Prognostic Index Based on a Metabolic-Genomic Landscape Analysis of Hepatocellular Carcinoma (HCC). Cancer Manag Res. 2021 13: 5683-5698. doi: 10.2147/CMAR.S316588 - Demissei BG, Freedman G, Feigenberg SJ, Plastaras JP, Maity A, Smith AM et al. Early Changes in Cardiovascular Biomarkers with Contemporary Thoracic Radiation Therapy for Breast Cancer, Lung Cancer, and Lymphoma. Int J Radiat Oncol Biol Phys. 2019 103(4): 851-860. doi: 10.1016/j.ijrobp.2018.11.013 - Krishnan S, Szabo E, Burghardt I, Frei K, Tabatabai G, Weller M. Modulation of cerebral endothelial cell function by TGF-β in glioblastoma: VEGF-dependent angiogenesis versus endothelial mesenchymal transition. Oncotarget. 2015 6(26): 22480-95. doi: 10.18632/oncotarget.4310 - Vishwamitra D, Shi P, Wilson D, Manshouri R, Vega F, Schlette EJ et al. Expression and effects of inhibition of type I insulin-like growth factor receptor tyrosine kinase in mantle cell lymphoma. Haematologica. 2011 96(6): 871-80. doi: 10.3324/haematol.2010.031567 - Dahlqvist J, Klar J, Hausser I, Anton-Lamprecht I, Pigg MH, Gedde-Dahl T Jr et al. Congenital ichthyosis: mutations in ichthyin are associated with specific structural abnormalities in the granular layer of epidermis. J Med Genet. 2007 44(10): 615-20. doi: 10.1136/jmg.2007.050542 - Canavese C, Bergamo D, Ciccone G, Longo F, Fop F, Thea A et al. Validation of serum ferritin values by magnetic susceptometry in predicting iron overload in dialysis patients. Kidney Int. 2004 65(3): 1091-8. doi: 10.1111/j.1523-1755.2004.00480.x - Ma Y, Kong LR, Ge Q, Lu YY, Hong MN, Zhang Y et al. Complement 5a-mediated trophoblasts dysfunction is involved in the development of pre-eclampsia. J Cell Mol Med. 2018 22(2): 1034-1046. doi: 10.1111/jcmm.13466 - Gurnadi JI, Mose J, Handono B, Satari MH, Anwar AD, Fauziah PN et al. Difference of concentration of placental soluble fms-like tyrosine kinase-1(sFlt-1), placental growth factor (PlGF), and sFlt-1/PlGF ratio in severe preeclampsia and normal pregnancy. BMC Res Notes. 2015 8: 534. doi: 10.1186/s13104-015-1506-0 - Halkes CJM, Zoutman WH, van der Fits L, Jedema I, Vermeer MH. Mutation in PIGA results in a CD52-negative escape variant in a Sézary syndrome patient during alemtuzumab treatment. J Invest Dermatol. 2015 135(4): 1199-1202. doi: 10.1038/jid.2014.501 - Aydinok Y, Piga A, Origa R, Mufti N, Erickson A, North A et al. Amustaline-glutathione pathogen-reduced red blood cell concentrates for transfusion-dependent thalassaemia. Br J Haematol. 2019 186(4): 625-636. doi: 10.1111/bjh.15963 - Asahina H, Tamura Y, Nokihara H, Yamamoto N, Seki Y, Shibata T et al. An open-label, phase 1 study evaluating safety, tolerability, and pharmacokinetics of linifanib (ABT-869) in Japanese patients with solid tumors. Cancer Chemother Pharmacol. 2012 69(6): 1477-86. doi: 10.1007/s00280-012-1846-6 - Tran M, Latifoltojar A, Neves JB, Papoutsaki MV, Gong F, Comment A et al. First-in-human in vivo non-invasive assessment of intra-tumoral metabolic heterogeneity in renal cell carcinoma. BJR Case Rep. 2019 5(3): 20190003. doi: 10.1259/bjrcr.20190003 - de Brito Junior LC, Cardoso Mdo S, Rocha EG, Anijar H, Cunha M, Saraiva JC. Frequency of paroxysmal nocturnal hemoglobinuria in patients attended in Belém, Pará, Brazil. Rev Bras Hematol Hemoter. 2011 33(1): 35-7. doi: 10.5581/1516-8484.20110012. Erratum in: Rev Bras Hematol Hemoter. 2011 33(2): 167 - Vishwamitra D, Curry CV, Alkan S, Song YH, Gallick GE, Kaseb AO et al. The transcription factors Ik-1 and MZF1 downregulate IGF-IR expression in NPM-ALK⁺ T-cell lymphoma. Mol Cancer. 2015 14: 53. doi: 10.1186/s12943-015-0324-2 - Erez O, Romero R, Maymon E, Chaemsaithong P, Done B, Pacora P et al. The prediction of late-onset preeclampsia: Results from a longitudinal proteomics study. PLoS One. 2017 12(7): e0181468. doi: 10.1371/journal.pone.0181468 - Roggero S, Quarello P, Vinciguerra T, Longo F, Piga A, Ramenghi U. Severe iron overload in Blackfan-Diamond anemia: a case-control study. Am J Hematol. 2009 84(11): 729-32. doi: 10.1002/ajh.21541 - Angelucci E, Barosi G, Camaschella C, Cappellini MD, Cazzola M, Galanello R et al. Italian Society of Hematology practice guidelines for the management of iron overload in thalassemia major and related disorders. Haematologica. 2008 93(5): 741-52. doi: 10.3324/haematol.12413 - Rahbari NN, Schmidt T, Falk CS, Hinz U, Herber M, Bork U et al. Expression and prognostic value of circulating angiogenic cytokines in pancreatic cancer. BMC Cancer. 2011 11: 286. doi: 10.1186/1471-2407-11-286 - Busca A, Falda M, Manzini P, D'Antico S, Valfrè A, Locatelli F et al. Iron overload in patients receiving allogeneic hematopoietic stem cell transplantation: quantification of iron burden by a superconducting quantum interference device (SQUID) and therapeutic effectiveness of phlebotomy. Biol Blood Marrow Transplant. 2010 16(1): 115-22. doi: 10.1016/j.bbmt.2009.09.011 - Pérez-Valencia JA, Prosdocimi F, Cesari IM, da Costa IR, Furtado C, Agostini M et al. Angiogenesis and evading immune destruction are the main related transcriptomic characteristics to the invasive process of oral tongue cancer. Sci Rep. 2018 8(1): 2007. doi: 10.1038/s41598-017-19010-5 - Kim WY, Kim MJ, Moon H, Yuan P, Kim JS, Woo JK et al. Differential impacts of insulin-like growth factor-binding protein-3 (IGFBP-3) in epithelial IGF-induced lung cancer development. Endocrinology. 2011 152(6): 2164-73. doi: 10.1210/en.2010-0693 - Korkama ES, Armstrong AE, Jarva H, Meri S. Spontaneous Remission in Paroxysmal Nocturnal Hemoglobinuria-Return to Health or Transition Into Malignancy? Front Immunol. 2018 9: 1749. doi: 10.3389/fimmu.2018.01749 - Govender N, Naicker T, Moodley J. Maternal imbalance between pro-angiogenic and anti-angiogenic factors in HIV-infected women with pre-eclampsia. Cardiovasc J Afr. 2013 24(5): 174-9. doi: 10.5830/CVJA-2013-029 - Ghosh A, Freestone NS, Anim-Nyame N, Arrigoni FIF. Microvascular function in pre-eclampsia is influenced by insulin resistance and an imbalance of angiogenic mediators. Physiol Rep. 2017 5(8): e13185. doi: 10.14814/phy2.13185 - Falk DJ, Todd AG, Lee S, Soustek MS, ElMallah MK, Fuller DD et al. Peripheral nerve and neuromuscular junction pathology in Pompe disease. Hum Mol Genet. 2015 24(3): 625-36. doi: 10.1093/hmg/ddu476 - Lassig AAD, Joseph AM, Lindgren BR, Yueh B. Association of Oral Cavity and Oropharyngeal Cancer Biomarkers in Surgical Drain Fluid With Patient Outcomes. JAMA Otolaryngol Head Neck Surg. 2017 143(7): 670-678. doi: 10.1001/jamaoto.2016.3595 - Fracchiolla NS, Barcellini W, Bianchi P, Motta M, Fermo E, Cortelezzi A. Biological and molecular characterization of PNH-like lymphocytes emerging after Campath-1H therapy. Br J Haematol. 2001 112(4): 969-71. doi: 10.1046/j.1365-2141.2001.02677.x - Joutel A, Andreux F, Gaulis S, Domenga V, Cecillon M, Battail N et al. The ectodomain of the Notch3 receptor accumulates within the cerebrovasculature of CADASIL patients. J Clin Invest. 2000 105(5): 597-605. doi: 10.1172/JCI8047 - Ayi K, Turrini F, Piga A, Arese P. Enhanced phagocytosis of ring-parasitized mutant erythrocytes: a common mechanism that may explain protection against falciparum malaria in sickle trait and beta-thalassemia trait. Blood. 2004 104(10): 3364-71. doi: 10.1182/blood-2003-11-3820 - Vatish M, Strunz-McKendry T, Hund M, Allegranza D, Wolf C, Smare C. sFlt-1/PlGF ratio test for pre-eclampsia: an economic assessment for the UK. Ultrasound Obstet Gynecol. 2016 48(6): 765-771. doi: 10.1002/uog.15997 - Vilsmaier T, Rack B, Janni W, Jeschke U, Weissenbacher T; SUCCESS Study Group. Angiogenic cytokines and their influence on circulating tumour cells in sera of patients with the primary diagnosis of breast cancer before treatment. BMC Cancer. 2016 16: 547. doi: 10.1186/s12885-016-2612-7 - Piolatto A, Berchialla P, Allegra S, De Francia S, Ferrero GB, Piga A. Pharmacological and clinical evaluation of deferasirox formulations for treatment tailoring. Sci Rep. 2021 11(1): 12581. doi: 10.1038/s41598-021-91983-w - Pennell DJ, Berdoukas V, Karagiorga M, Ladis V, Piga A, Aessopos A et al. Randomized controlled trial of deferiprone or deferoxamine in beta-thalassemia major patients with asymptomatic myocardial siderosis. Blood. 2006 107(9): 3738-44. doi: 10.1182/blood-2005-07-2948 - Quao ZC, Tong M, Bryce E, Guller S, Chamley LW, Abrahams VM. Low molecular weight heparin and aspirin exacerbate human endometrial endothelial cell responses to antiphospholipid antibodies. Am J Reprod Immunol. 2018 79(1): 10.1111/aji.12785. doi: 10.1111/aji.12785 - Newell LF, Holtan SG, Yates JE, Pereira L, Tyner JW, Burd I et al. PlGF enhances TLR-dependent inflammatory responses in human mononuclear phagocytes. Am J Reprod Immunol. 2017 78(4): 10.1111/aji.12709. doi: 10.1111/aji.12709 - Cappellini MD, Cohen A, Piga A, Bejaoui M, Perrotta S, Agaoglu L. A phase 3 study of deferasirox (ICL670), a once-daily oral iron chelator, in patients with beta-thalassemia. Blood. 2006 107(9): 3455-62. doi: 10.1182/blood-2005-08-3430 |
| Studies on GPIBD cases describing aspects other than symptomatology | - Lefeber DJ, Schönberger J, Morava E, Guillard M, Huyben KM, Verrijp K et al. Deficiency of Dol-P-Man synthase subunit DPM3 bridges the congenital disorders of glycosylation with the dystroglycanopathies. Am J Hum Genet. 2009 85(1): 76-86. doi: 10.1016/j.ajhg.2009.06.006 - Yuan X, Li Z, Baines AC, Gavriilaki E, Ye Z, Wen Z et al. A hypomorphic PIGA gene mutation causes severe defects in neuron development and susceptibility to complement-mediated toxicity in a human iPSC model. PLoS One. 2017 12(4): e0174074. doi: 10.1371/journal.pone.0174074 - Kawamoto M, Murakami Y, Kinoshita T, Kohara N. Recurrent aseptic meningitis with PIGT mutations: a novel pathogenesis of recurrent meningitis successfully treated by eculizumab. BMJ Case Rep. 2018 2018: bcr2018225910. doi: 10.1136/bcr-2018-225910 - Almeida AM, Murakami Y, Baker A, Maeda Y, Roberts IA, Kinoshita T et al. Targeted therapy for inherited GPI deficiency. N Engl J Med. 2007 356(16): 1641-7. doi: 10.1056/NEJMoa063369 - Ratliff M, Zhu W, Deshmukh R, Wilks A, Stojiljkovic I. Homologues of neisserial heme oxygenase in gram-negative bacteria: degradation of heme by the product of the pigA gene of Pseudomonas aeruginosa. J Bacteriol. 2001 183(21): 6394-403. doi: 10.1128/JB.183.21.6394-6403.2001 - Chen G, Ye Z, Yu X, Zou J, Mali P, Brodsky RA et al. Trophoblast differentiation defect in human embryonic stem cells lacking PIG-A and GPI-anchored cell-surface proteins. Cell Stem Cell. 2008 2(4): 345-55. doi: 10.1016/j.stem.2008.02.004 - Dagan R, Cleper R, Davidovits M, Sinai-Trieman L, Krause I. Post-Infectious Glomerulonephritis in Pediatric Patients over Two Decades: Severity-Associated Features. Isr Med Assoc J. 2016 18(6): 336-40 - de la Morena-Barrio ME, Hernández-Caselles T, Corral J, García-López R, Martínez-Martínez I, Pérez-Dueñas B et al. GPI-anchor and GPI-anchored protein expression in PMM2-CDG patients. Orphanet J Rare Dis. 2013 8: 170. doi: 10.1186/1750-1172-8-170 - Carmody LC, Blau H, Danis D, Zhang XA, Gourdine JP, Vasilevsky N et al. Significantly different clinical phenotypes associated with mutations in synthesis and transamidase+remodeling glycosylphosphatidylinositol (GPI)-anchor biosynthesis genes. Orphanet J Rare Dis. 2020 15(1): 40. doi: 10.1186/s13023-020-1313-0 - Cacciapuoti C, Terrazzano G, Barone L, Sica M, Becchimanzi C, Rotoli B et al. Glycosyl-phosphatidyl-inositol-defective granulocytes from paroxysmal nocturnal haemoglobinuria patients show increased bacterial ingestion but reduced respiratory burst induction. Am J Hematol. 2007 82(2): 98-107. doi: 10.1002/ajh.20779 - Norris J, Hall S, Ware RE, Kamitani T, Chang HM, Yeh E et al. Glycosyl-phosphatidylinositol anchor synthesis in paroxysmal nocturnal hemoglobinuria: partial or complete defect in an early step. Blood. 1994 83(3): 816-21 - Schubert J, Schmidt RE, Medof ME. Regulation of glycoinositol phospholipid anchor assembly in human lymphocytes. Absent mannolipid synthesis in affected T and natural killer cell lines from paroxysmal nocturnal hemoglobinuria patients. J Biol Chem. 1993 268(9): 6281-7 - Hillmen P, Bessler M, Mason PJ, Watkins WM, Luzzatto L. Specific defect in N-acetylglucosamine incorporation in the biosynthesis of the glycosylphosphatidylinositol anchor in cloned cell lines from patients with paroxysmal nocturnal hemoglobinuria. Proc Natl Acad Sci U S A. 1993 90(11): 5272-6. doi: 10.1073/pnas.90.11.5272 |
